# Remote Monitoring Approaches to Reduce Readmissions After Infection and Sepsis: A Randomized Clinical Trial

**DOI:** 10.1001/jamanetworkopen.2026.16641

**Published:** 2026-06-11

**Authors:** Sachin Yende, Victor B. Talisa, Kristin Mayes, Kelly Williams, Adelina Malito, Florian B. Mayr, Derek C. Angus, Rana Awdish, Qingfeng Liang, Kimberly J. Rak, Jacqueline Barnes, Elizabeth Lorenzi, Kert Viele, Chung-Chou H. Chang, Casey McCauley, Melanie Quintana, Anna McGlothlin, Farah Khandwala, Jatin Dave

**Affiliations:** 1 The Clinical Research, Investigation, and Systems Modeling of Acute Illness Center, Department of Critical Care Medicine, University of Pittsburgh, Pittsburgh, Pennsylvania; 2 Integrated Veterans Care, Veterans Health Administration, Washington, DC; 3 UPMC (University of Pittsburgh Medical Center) Center for High-Value Health Care, Pittsburgh, Pennsylvania; 4 Veteran Affairs Pittsburgh Healthcare System, Pittsburgh, Pennsylvania; 5 Henry Ford Hospital, Detroit, Michigan; 6 Michigan State College of Human Medicine, Grand Rapids; 7 Berry Consultants LLC, Austin, Texas; 8 Department of Medicine, University of Pittsburgh, Pittsburgh, Pennsylvania; 9 UPMC Health Plan, Pittsburgh, Pennsylvania

## Abstract

**Importance:**

The Centers for Medicare & Medicaid Services reimburses remote monitoring to reduce hospital readmissions, yet its effectiveness remains uncertain.

**Objective:**

To evaluate effectiveness of remote monitoring in reducing readmissions following hospitalizations for serious infections overall and across prespecified subgroups.

**Design, Setting, and Participants:**

This randomized clinical trial was conducted from March 25, 2021, to December 9, 2024, using response adaptive randomization among postdischarge patients with sepsis or lower respiratory tract infection across 19 hospitals. Eligible patients were 21 years or older, lived in western Pennsylvania, were insured through the UPMC Health Plan or traditional fee-for-service Medicare, had a smartphone or other internet-connected device, had no cognitive impairment, and were at moderate or high risk for readmission at index hospitalization admission based on an internal predictive model. Analyses were based on intention to treat; data were analyzed from April to June 2025.

**Intervention:**

Four remote monitoring strategies combining questionnaire intensity (low vs high) and clinical response teams (standard vs enhanced) compared with usual care.

**Main Outcomes and Measures:**

The primary end point consisted of days at home at 90 days after discharge, assessed by posterior probability distribution for the cumulative odds ratios (CORs) in each study arm compared with usual care. Secondary end points included mortality, readmission, functional status, quality of life, and use of emergency department and hospice services.

**Results:**

Among the 1286 patients included in the analysis, the median age was 63 (IQR, 54-71) years; 665 patients (51.7%) were female. The median Charlson Comorbidity Index was 6 (IQR, 3-9), and 386 patients (32.6%) received intensive care. Patients were randomized to usual care (n = 399) or remote patient monitoring (RPM) with low- or high-intensity questionnaires and standard or enhanced clinical response team combinations: RPM-low standard response (n = 204), RPM-high standard response (n = 129), RPM-low enhanced response (n = 383), and RPM-high enhanced response (n = 171). Of 887 patients assigned to remote monitoring arms, 529 (59.6%) enrolled in the remote monitoring program. The median home days were similar across all study arms: 90 (IQR, 83-90) for usual care, 90 (IQR, 84-90) for RPM-low standard response, 90 (IQR, 85-90) for RPM-high standard response, 90 (IQR, 83-90) for RPM-low enhanced response, and 90 (IQR, 84-90) for RPM-high enhanced response. Compared with usual care, the CORs were 0.96 (credible interval [CrI], 0.70-1.32) for RPM-low standard response, 0.86 (95% CrI, 0.60-1.23) for RPM-high standard response, 1.01 (95% CrI, 0.76-1.33) for RPM-low enhanced response, and 0.96 (95% CrI, 0.69-1.36) for RPM-high enhanced response, and superiority probability was less than 55% for all comparisons. At least 1 readmission occurred in 151 of 399 patients (37.8%) in the usual care arm, 81 of 204 (39.7%) in the RPM-low standard response arm, 57 of 129 (44.2%) in the RPM-high standard response arm, 143 of 383 (37.3%) in the RPM-low enhanced response arm, and 62 of 171 (36.3%) in the RPM-high enhanced response arm. Among patients 65 years and older, standard and enhanced response arms had fewer home days compared with usual care (COR, 0.56 [95% CrI, 0.36-0.85] and 0.67 [95% CrI, 0.45-0.98], respectively; inferiority probability, 99.6% and 97.9%, respectively).

**Conclusions and Relevance:**

Among trial patients discharged after hospitalization for serious infections, remote monitoring did not increase time spent alive at home but reduced it in those 65 years and older. These findings support reevaluating and tailoring remote monitoring after acute care for sepsis and lower respiratory tract infection to support further alignment with patients’ needs and desire for personalized monitoring.

**Trial Registration:**

ClinicalTrials.gov Identifier: NCT04829188

## Key Points

**Question:**

Does remote monitoring help individuals remain at home following hospitalization for serious infections?

**Findings:**

In this randomized clinical trial of 1286 adults recovering at home after hospitalization for sepsis or lower respiratory tract infection, remote monitoring programs with high- or low-intensity question sets combined with a nurse response team or a nurse practitioner–led response team did not increase time spent at home. Conversely, the remote monitoring programs led to a reduction in time spent at home among Medicare-eligible patients 65 years or older.

**Meaning:**

Remote monitoring, as implemented in this study, did not support Medicare’s goals of reducing readmissions, particularly among older adults.

## Teaser

This randomized clinical trial evaluates the effectiveness of remote monitoring programs led by nurses or nurse practitioners to reduce readmissions among patients after hospitalization for sepsis or lower respiratory tract infections.

## Introduction

More than 3 million people in the US are hospitalized annually for serious infections such as influenza, sepsis, and COVID-19.^1,2,3^ These hospitalizations are a leading cause of readmissions,^4^ which are regarded by the Centers for Medicare & Medicaid Services (CMS) as an indicator of poor care quality.^5^ Readmissions also negatively impact patient and caregiver health and accompanying costs.^6^ Efforts have aimed to reduce readmissions, but few have been rigorously evaluated by randomized clinical trials (RCTs), and none have identified effective interventions.^7,8^

Remote monitoring, a CMS-reimbursed telehealth strategy, has been shown to reduce readmissions for heart failure, but its effectiveness is mixed or understudied for other conditions,^9,10^ including serious infections.^11^ Remote monitoring may improve access to care to underserved populations, including rural and low-income residents, many of whom own smartphones.^12,13^ Between 2019 and 2022, remote monitoring use among Medicare beneficiaries increased 10-fold, with the largest growth among Black and low-income enrollees.^14^ Remote monitoring enables the collection of health data through smartphone application–based questionnaires and condition-specific text messages, often referred to as remote therapeutic monitoring, or through physiologic measurements, known as remote physiologic monitoring. These data help identify signs of clinical deterioration and trigger alerts, prompting nurse-led interventions that function as initial clinical response strategies and can be escalated to other clinicians as needed.^15^ However, evaluating the effectiveness of different monitoring approaches and associated response strategies using traditional trial designs often requires large sample sizes. We used an adaptive design allowing for real-time adjustments in intervention assignments based on emerging data to efficiently identify the most effective intervention. We compared 4 experimental arms combining different therapeutic monitoring and response team approaches with usual care. We nested this trial in an integrated delivery and finance system that enabled patient tracking through electronic health records and payer claims data to minimize loss to follow-up, a common limitation in post–acute care trials.

## Methods

### Study Design and Oversight

We conducted a mixed-methods RCT designed to evaluate comparative effectiveness of interventions in a clinical setting using staff who routinely treat a broad patient population in standard (nonresearch) clinical practice to maximize applicability of results to everyday clinical practice. Recruitment occurred from March 25, 2021, to September 3, 2024, and the intervention ended December 8, 2024. The full design and methods are described elsewhere,^16^ and the trial protocol and statistical analysis plan are found in Supplement 1. We had 3 objectives: (1) to evaluate the effectiveness of remote therapeutic monitoring approaches for patients hospitalized for sepsis or lower respiratory tract infection compared with usual care; (2) to examine whether effectiveness varied across patient subgroups; and (3) to qualitatively explore implementation barriers and facilitators.

The University of Pittsburgh Institutional Review Board approved the study, and written informed consent was obtained from patients by the research team. Independent data and safety monitoring and stakeholder advisory boards performed oversight. The trial followed the Consolidated Standards of Reporting Trials (CONSORT) reporting guideline to ensure complete reporting of trial design, participant flow, outcomes, and analyses. Qualitative data were collected and analyzed in accordance with the Patient-Centered Outcomes Research Institute (PCORI) methodology standards for qualitative research^17^ and reported according to the Standard for Reporting Qualitative Research (SRQR) reporting guideline.

### Patients

We enrolled patients 21 years or older who lived in western Pennsylvania, were insured through the UPMC Health Plan (which offers commercial, Medicare Advantage, Medicaid, and dual eligible special needs plans) or traditional fee-for-service Medicare, were hospitalized at 1 of 13 UPMC or 6 independently owned hospitals for sepsis or a lower respiratory tract infection (eTable 1 in Supplement 2), had a smartphone or other internet-connected device, had no cognitive impairment,^18^ and were at moderate or high risk for readmission at index hospitalization admission based on an internal predictive model (eTable 2 in Supplement 2). The model is calculated for all UPMC patients; considers prior use of health care services, comorbidities, medication use, and risk factors; and was trained to estimate 7- and 30-day readmissions.

We excluded patients admitted from settings other than home or independent living, who were enrolled in hospice at hospital admission, or who were discharged to a long-term acute care or skilled nursing facility for more than 28 days, as these circumstances may lead to readmissions unrelated to sepsis or infection (eTable 3 in Supplement 2). Race was self-reported as Black, White, or other (including American Indian or Alaska Native, Asian, Native Hawaiian or Other Pacific Islander, multiracial, or unspecified); ethnicity was self-reported as Hispanic or non-Hispanic. Race and ethnicity data were collected because of documented differences between various use of mobile health technology by different demographic groups and how those differences may impact the effect of the intervention studied here.^19,20,21,22^

### Randomization and Blinding

Following consent, we reconfirmed eligibility after discharge to home and randomized on the first weekday at home. For patients sent to a skilled nursing facility after discharge, we ensured they returned home within 28 days before randomization. Because of the nature of the intervention, the trial was open-label, and patients, the clinical implementation teams, and study coordinators were aware of intervention assignments, but most study investigators and clinical teams who made readmission decisions were blinded.

### Interventions

Patients who survive sepsis or lower respiratory tract infection experience a 1-year mortality rate of approximately 40%,^23^ and more than 60% of hospital readmissions are driven by infections and exacerbations of heart and lung disease.^24,25^ Therefore, our interventions targeted early detection and management of recurrent infections, chronic obstructive pulmonary disease, and heart failure, and timely palliative care and hospice transitions.

Our goal was to evaluate remote therapeutic monitoring of patient-reported problems, rather than physiologic data, on the premise that the patient-reported symptoms and issues, such as medication nonadherence, may better capture early precursors to readmission.^26^ Monitoring can vary both in scope of problems tracked, from a narrow to a broader inventory of potential problems, which may increase patient burden, and how the clinical response is executed, from reliance on a standard nurse call center team to an enhanced multidisciplinary team that offers broader expertise and timely interventions but requires greater resources and may fragment care. We generated 4 intervention arms, combining narrow (low-intensity) vs broad (high-intensity) questionnaires with either standard or enhanced response teams and closely followed CMS guidance: remote patient monitoring–low intensity (RPM-low) with the standard response team, RPM-low with the enhanced response team, remote patient monitoring–high intensity (RPM-high) with the standard response team, and RPM-high with the enhanced response team.^15^ We also included a fifth control (usual care) arm. The low-intensity arms received questions to detect repeated infections, the most common cause of readmission, while the high-intensity arms involved broader monitoring for infection, heart failure, and chronic obstructive pulmonary disease (eTable 4 in Supplement 2 provides details).^25^ Regardless of the type of questions administered, patients received questions twice weekly, their responses triggered alerts categorized as medium or high, and the standard or enhanced team responded to these alerts. To support enrollment and sustain engagement, we implemented several strategies, including patient education, frequent reminders, real-time feedback, and ongoing technology support.

The standard team consisted of 4 remote monitoring nurses who responded to alerts and coordinated care with patients’ primary or specialty clinicians. Nurses had access to the shared electronic health record used by many patients’ primary care teams and could communicate with those teams directly. The enhanced team included 2 different nurses and 2 certified registered nurse practitioners with palliative care expertise and access to social workers. These nurse practitioners could diagnose medical conditions, develop and implement a treatment plan, order and perform diagnostic tests, and deliver other health care services, pursuant to a written collaborative agreement with a licensed physician. We incorporated palliative care expertise because prior studies demonstrate high 1-year mortality after pneumonia and sepsis and frequent delays in hospice referral.^27^ Early palliative care involvement has been associated with improved quality of life, more timely hospice use, and reductions in hospital readmissions.^28,29^ Prior to the trial, both teams received training from a critical care physician on important signs of worsening sepsis or pneumonia infection and management in the postdischarge population. Team members were supervised by physicians specializing in geriatric and palliative care. In addition to responding to alerts, the enhanced team conducted virtual visits during the first week after discharge, completed assessments including medication reviews, and initiated completion of an advance directive, including documentation of cardiopulmonary resuscitation status.^30^

Usual care included structured telephone support, based on the Agency for Healthcare Research and Quality Re-engineered Discharge Toolkit,^31^ and involved nurses or care managers calling patients during the first week after discharge to assess their condition, review discharge instructions and home-care needs, reconcile medications, and ensure that follow-up appointments were scheduled. An intervention started when the patient returned home after the index admission and was paused while the patient was readmitted.

### Outcomes

The primary end point was postdischarge home days, a composite of survival and the number of days the patient was at home within the 90 days following hospital discharge and excluded time spent in the hospital and skilled nursing, rehabilitation, and long-term ventilatory care facilities. This end point has been used previously^32,33,34,35,36^; it captures survival and quality of life and is a practical, patient-centered measure of outcomes that are meaningful to both patients and health care delivery systems. Participants who died during the 90-day period were assigned home days of −1, consistent with established composite outcome frameworks, such as organ support–free days and ventilator-free days, in which death is defined as the worst outcome and is assigned a value lower than that of any survivor. This approach avoids paradoxical interpretations in which early death appears more favorable than prolonged hospitalization, maintains the ordinal structure of the outcome, and permits death to be incorporated into a single interpretable measure. For patients discharged from skilled nursing facilities, the 90-day period began on discharge from the facility. Functional status,^37,38^ quality of life,^39^ use of the emergency department and hospice services, readmissions, and mortality were secondary end points (eTable 5 in Supplement 2).

### Statistical Analysis

We used responsive adaptive randomization^40^ in the intervention arms to increase the likelihood that patients were assigned to the most effective intervention, based on postdischarge home days, as data accrued, while keeping usual care allocation fixed to improve trial efficiency and ethical equipoise. Following a 250-patient burn-in period, during which randomization was fixed at 30%^40^ for usual care and 70% distributed evenly across the 4 intervention arms on a 1:1:1:1 basis, the allocation sequence was updated quarterly after each interim analysis and determined based on the posterior probability that each intervention was most effective at the time of analysis.

We analyzed all data according to the intention-to-treat principle; data were analyzed from April to June 2025. We used a bayesian proportional odds model^41^ for the primary analysis, calculating the posterior probability distribution for the cumulative odds ratios (CORs) to compare postdischarge home days for each intervention arm with usual care, which ranged from –1 to 90. Patients who died during follow-up were assigned a value of –1. Thus, the CORs capture the cumulative probability of experiencing the discrete outcomes of death or readmission at each time point from 0 to 90 days. A COR greater than 1.00 implies that the intervention increased home days while a COR less than 1.00 implies that the intervention reduced home days compared with usual care. The bayesian model included terms for the study interventions and their interaction and controlled for calendar time, illness severity, and discharge location. All terms were assigned weakly informative priors, and the interaction was given a dynamic prior to flexibly accommodate a range of potential interactions. We used a Markov Chain Monte Carlo algorithm^42^ to draw 100 000 samples from the joint posterior distribution for all model parameters and generated posterior distributions for CORs, 95% credible intervals (CrIs), posterior probabilities of superiority for each intervention arm compared with usual care, and the probability that each intervention arm was optimal. Stopping criteria for superiority (posterior probability of >99.1% of a COR >1.00) or futility (posterior probability <40% of a COR >1.00) were assessed quarterly.

We modeled secondary end points using appropriate bayesian linear or generalized linear models, which were similar in structure and used similar priors as the primary model. We conducted sensitivity analyses to assess the assumptions of the proportional odds model, impact of patients receiving fee-for-service Medicare, maintenance of health insurance through UPMC Health Plan membership, and marginal effects of monitoring intensity and response teams. We also estimated the subdistribution hazard ratio for readmission using a frequentist Fine-Gray model accounting for death as a competing risk and baseline covariates as in the primary analysis, as well as unadjusted subdistribution cumulative incidence rates.^43^

We assessed differential effects of the interventions across prespecified subgroups, including age, sex, race and ethnicity, health literacy, comfort with technology, Charlson Comorbidity Index, area deprivation index, discharge location, severity of illness, and diagnosis at index admission. In post hoc analyses, we examined a subgroup defined by enrollment in remote monitoring and patients enrolled when hospital capacity and admissions were limited due to COVID-19 surges (eMethods in Supplement 2). Analyses were conducted using bayesian cumulative logistic regression models that included interaction terms between intervention and subgroup.

We estimated a sample size of 1500 using a 10% attrition rate, based on simulations indicating adequate power to identify a range of effectiveness scenarios for 1 or more interventions (eTable 6 in Supplement 2). For example, we had greater than 85% power to detect a COR of 1.50 if at least 1 intervention was effective, equivalent to a 9% increase in patients surviving 90 days without a readmission. In November 2023, the trial was resized to 1281 patients in consultation with the data safety monitoring board and the funding agency due to the lower than expected observed attrition rate of 2.5% and recruitment challenges due to COVID-19, including social distancing restrictions in hospitals and lower than expected rates of respiratory tract infection. Power was maintained above 80% for most effectiveness scenarios and for similar absolute differences in patients surviving 90 days at home due to a lower attrition rate in the trial population compared with the rates used in the simulations at the onset of the trial (eTable 7 in Supplement 2). Analyses were conducted using R, version 4.4.3 (R Project for Statistical Computing)^44^ and Stan, version 2.32.2, with the rstan package (R Project for Statistical Computing).^45^

We conducted semistructured telephone interviews in a subset of patients across the 4 intervention arms and analyzed themes around engagement and intervention impact. Interviews were analyzed using a hybrid deductive-inductive approach to thematic analysis guided by an implementation outcomes approach.^46^ Interview facilitation and analytic details are provided in the eMethods in Supplement 2.

## Results

### Patients

**Figure 1.**
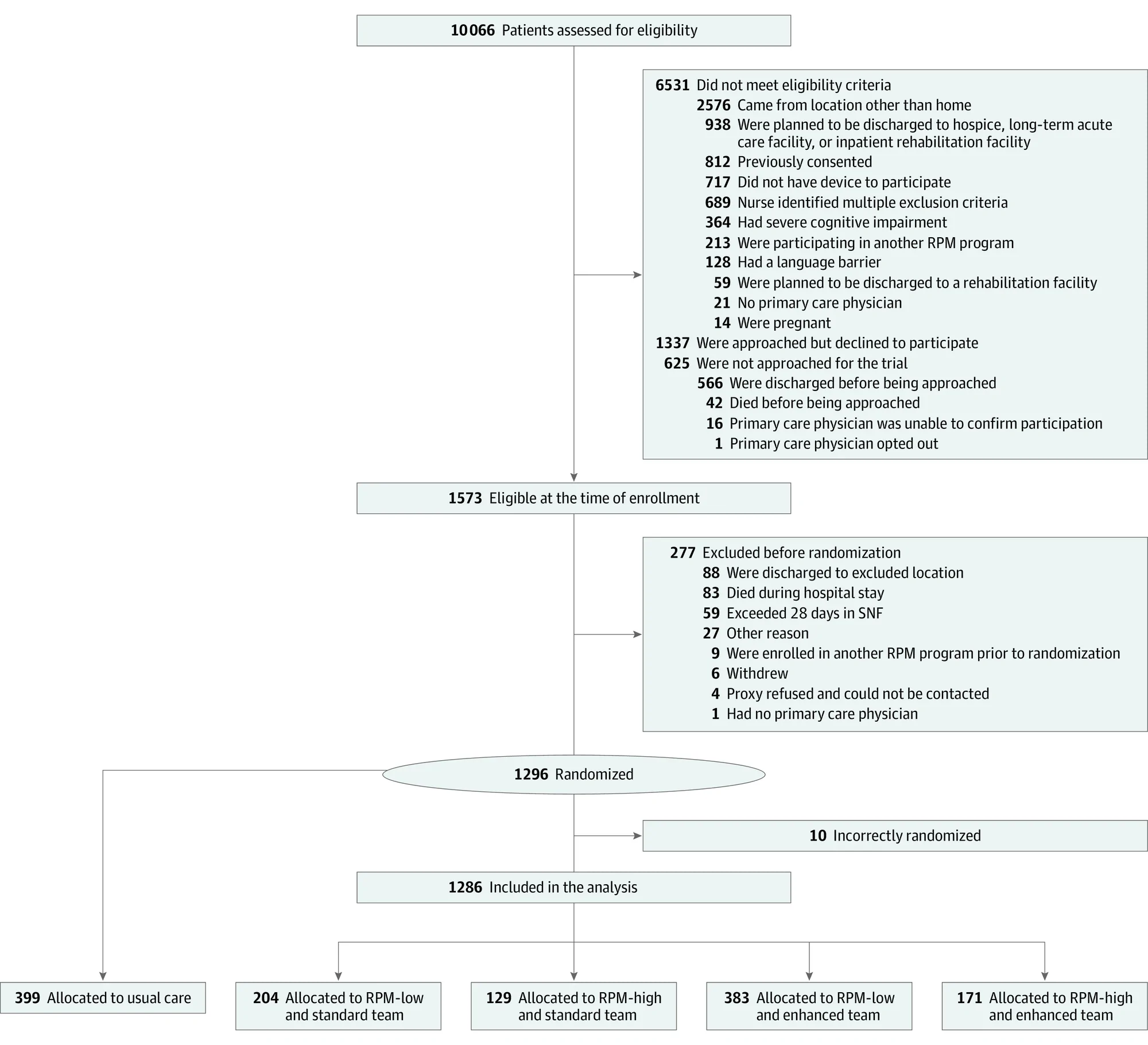
Flow Diagram of Screening, Randomization, and Follow-Up of Participants RPM indicates remote patient monitoring; SNF, skilled nursing facility.

Among 10 066 screened patients, 8493 were ineligible, 1337 of whom declined participation (Figure 1). Of the 1573 patients who consented, 277 were ineligible at hospital discharge and 10 were incorrectly randomized, yielding a final analysis cohort of 1286. Of these, 399 were assigned to usual care, 204 to RPM-low standard response, 129 to RPM-high standard response, 383 to RPM-low enhanced response, and 171 to RPM-high enhanced response. The median age was 63 (IQR, 54-71) years; 665 patients (51.7%) were female, 595 (46.3%) were male, and 26 (2.0%) were agender, gender queer, or transgender. In terms of race, 199 patients (15.5%) were Black, 1029 (80.0%) were White, and 58 (4.5%) were of other race; in terms of ethnicity, 11 patients (1.0%) were Hispanic, 1211 (94.2%) were non-Hispanic, and 64 (5.0%) were of unknown ethnicity. The median Charlson Comorbidity Index was 6 (IQR, 3-9), indicating a significant comorbidity burden per patient. Charlson is a weighted sum of comorbidities where 0 represents no comorbidity and scores greater than 10 are uncommon.^47^ A total of 606 patients (47.1%) were hospitalized for sepsis, 367 (28.5%) for lower respiratory tract infection, and 313 (24.3%) for COVID-19 (Table 1). Almost one-third of patients (386 [32.6%]) were in the intensive care unit, with a median hospital stay of 7 (IQR, 4-11) days. Intervention arms were well matched at baseline (Table 1 and eTable 8 in Supplement 2). A total of 10 interim analyses were conducted, and the number of patients randomized and randomization probabilities over time are included in eFigures 1 and 2 in Supplement 2.

**Table 1.** Baseline Patient Characteristics

Characteristic	Patients, No. (%)						
	Total (N = 1286)	Usual care (n = 399)	Any care model (n = 887)	Individual care model			
				RPM-low standard response (n = 204)	RPM-high standard response (n = 129)	RPM-low enhanced response (n = 383)	RPM-high enhanced response (n = 171)
**Demographic**								
Age, median (IQR), y	63 (54-71)	64 (54-71)	63 (53-71)	63 (53-71)	63 (55-72)	62 (52-71)	64 (55-71)
Sex							
Female	665 (51.7)	194 (48.6)	471 (53.1)	108 (52.9)	60 (46.5)	209 (54.6)	94 (55.0)
Male	595 (46.3)	196 (49.1)	399 (45.0)	92 (45.1)	64 (49.6)	169 (44.1)	74 (43.3)
Other^a^	26 (2.0)	9 (2.3)	17 (1.9)	4 (2.0)	5 (3.9)	5 (1.3)	3 (1.8)
Race							
Black	199 (15.5)	66 (16.5)	133 (15.0)	32 (15.7)	22 (17.1)	61 (15.9)	18 (10.5)
White	1029 (80.0)	316 (79.2)	713 (80.4)	167 (81.9)	97 (75.2)	305 (79.6)	144 (84.2)
Other^b^	58 (4.5)	17 (4.3)	41 (4.6)	5 (2.5)	10 (7.8)	17 (4.4)	9 (5.3)
Ethnicity							
Hispanic	11 (1.0)	4 (1.0)	7 (0.8)	0	1 (0.8)	4 (1.0)	2 (1.2)
Non-Hispanic	1211 (94.2)	377 (94.5)	834 (94.0)	195 (95.6)	114 (88.4)	364 (95.0)	161 (94.2)
Unknown	64 (5.0)	18 (4.5)	46 (5.2)	9 (4.4)	14 (10.9)	15 (3.9)	8 (4.7)
**Health, educational attainment, and socioeconomic status before admission**								
Charlson Comorbidity Index, median (IQR)^c^	6 (3-9)	6 (4-9)	6 (3-8)	6 (3-8)	6 (3-8)	6 (3-9)	6 (3-9)
Prior inpatient admissions^d^							
0	941 (79.5)	291 (78.9)	650 (79.9)	148 (78.7)	98 (80.3)	283 (80.4)	121 (79.6)
1	96 (8.1)	32 (8.7)	64 (7.9)	19 (10.1)	10 (8.2)	26 (7.4)	9 (5.9)
2	69 (5.8)	24 (6.5)	45 (5.5)	11 (5.9)	5 (4.1)	17 (4.8)	12 (7.9)
≥3	77 (6.5)	22 (6.0)	55 (6.8)	10 (5.3)	9 (7.4)	26 (7.4)	10 (6.6)
Area deprivation index, median (IQR)^e^	7 (5-9)	8 (5-9)	7 (5-9)	8 (6-10)	7 (4-9)	7 (5-9)	7 (4-9)
Insurance payer							
UPMC Health Plan	1183 (92.0)	369 (92.5)	814 (91.8)	188 (92.2)	122 (94.6)	352 (91.9)	152 (88.9)
Fee-for-service Medicare	103 (8.0)	30 (8.1)	73 (8.2)	16 (7.8)	7 (5.4)	31 (8.1)	19 (11.1)
Marital status							
Married	349 (27.1)	115 (28.8)	234 (26.4)	41 (20.1)	42 (32.6)	103 (26.9)	48 (28.1)
Never married	214 (16.6)	69 (17.3)	145 (16.3)	29 (14.2)	22 (17.1)	62 (16.2)	32 (18.7)
Separated or divorced	161 (12.5)	51 (12.8)	110 (12.4)	26 (12.7)	16 (12.4)	46 (12.0)	22 (12.9)
Widowed	106 (8.2)	22 (5.5)	84 (9.5)	25 (12.3)	9 (7.0)	38 (9.9)	12 (7.0)
Unknown	456 (35.5)	142 (35.6)	314 (35.4)	83 (40.7)	40 (31.0)	134 (35.0)	57 (33.3)
Comfort with technology							
Comfortable	581 (45.2)	183 (45.9)	398 (44.9)	79 (38.7)	62 (48.1)	173 (45.2)	84 (49.1)
Uncomfortable	242 (18.8)	75 (18.8)	167 (18.8)	39 (19.1)	29 (22.5)	69 (18.0)	30 (17.5)
Unknown	463 (36.0)	141 (35.3)	322 (36.3)	86 (42.2)	38 (29.5)	141 (36.8)	57 (33.3)
Health literacy							
Limited	183 (14.2)	45 (11.3)	138 (15.6)	32 (15.7)	24 (18.6)	59 (15.4)	23 (13.5)
Marginal	268 (20.8)	85 (21.3)	183 (20.6)	33 (16.2)	26 (20.2)	95 (24.8)	29 (17.0)
Adequate	378 (29.4)	129 (32.3)	249 (28.1)	55 (27.0)	41 (31.8)	91 (23.8)	62 (36.3)
Unknown	457 (35.5)	140 (35.1)	317 (35.7)	84 (41.2)	38 (29.5)	138 (36.0)	57 (33.3)
**Index hospitalization**								
Hospital							
Bed size, median (IQR)	336 (145-1120)	336 (163-1120)	336 (145-1120)	336 (145-1120)	336 (145-1120)	336 (145-1120)	336 (179-1120)
Teaching status	1274 (99.1)	398 (99.7)	876 (98.8)	203 (99.5)	126 (97.7)	378 (98.7)	169 (98.8)
Admission diagnosis							
Lower respiratory tract infection	367 (28.5)	113 (28.3)	254 (28.6)	69 (33.8)	42 (32.6)	89 (23.2)	54 (31.6)
Sepsis	606 (47.1)	188 (47.1)	418 (47.1)	101 (49.5)	60 (46.5)	187 (48.8)	70 (40.9)
COVID-19	313 (24.3)	98 (24.6)	215 (24.2)	34 (16.7)	27 (20.9)	107 (27.9)	47 (27.5)
Mechanical ventilation^d^	62 (4.8)	23 (5.8)	39 (4.4)	15 (7.4)	5 (3.9)	12 (3.1)	7 (4.1)
Vasopressor use^d^	144 (12.2)	47 (12.7)	97 (11.9)	24 (12.8)	18 (14.8)	41 (11.6)	14 (9.2)
Dialysis^d^	100 (8.5)	37 (10.0)	63 (7.7)	16 (8.5)	9 (7.4)	30 (8.5)	8 (5.3)
Intensive care unit stay^d^	386 (32.6)	113 (30.6)	273 (33.5)	67 (35.6)	43 (35.2)	119 (33.8)	44 (28.9)
Length of stay in the hospital, median (IQR), d	7 (4-11)	7 (4-11)	7 (4-11)	7 (5-12)	7 (5-11)	7.0 (4-11)	7 (4-10)
Discharged to skilled nursing facility	110 (8.6)	36 (9.0)	74 (8.3)	21 (10.3)	7 (5.4)	32 (8.4)	14 (8.2)

Abbreviations: RPM, remote patient monitoring; UPMC, University of Pittsburgh Medical Center.

^a^ Includes agender, gender queer, and transgender.

^b^ Includes American Indian or Alaska Native, Asian, Native Hawaiian or Other Pacific Islander, multiracial, and unspecified.

^c^ Data were missing for 77 patients.

^d^ Includes patients in UPMC Health Plan only. Data were missing for 103 non–UPMC Health Plan members.

^e^ Calculated using state rankings. Scores range from 1 to 10, with higher scores indicating more disadvantaged neighborhoods in that state. Data were missing for 102 patients.

### Fidelity and Engagement

In the usual care arm, 257 of 399 patients (64.4%) received a follow-up call during the first week after discharge, with 201 of 257 (78.2%) answering. Among the 369 patients enrolled in the UPMC Health Plan, 157 (42.5%) completed a primary care visit within 7 days of discharge and 291 (78.9%) within 30 days. The corresponding 7- and 30-day primary care visit rates included 87 of 188 patients (46.3%) and 153 of 188 (81.4%), respectively, in the RPM-low standard response arm; 147 of 352 (41.8%) and 276 of 352 (78.4%), respectively, in the RPM-low enhanced response arm; 56 of 122 (45.9%) and 94 of 122 (77.0%), respectively, in the RPM-high standard response arm; and 71 of 152 (46.7%) and 120 of 152 (78.9%), respectively, in the RPM-high enhanced response arm. Further details are provided in eTable 9 in Supplement 2.

Among the 887 patients in the 4 intervention arms, 529 (59.6%) enrolled in remote monitoring and a total of 10 561 questionnaires were sent (median, 22 [IQR, 16-25] per patient), with 5922 responses (56.1%) (median, 13 [IQR, 4-16] per patient), triggering 3166 yellow and 425 red alerts (median, 4 [IQR, 2-9] and 1 [IQR, 1-3], respectively). Nurses responded to more than 94% of alerts. Despite questionnaire differences in low- and high-intensity questionnaire arms, response rates and alert volumes were similar. Enhanced teams also proactively contacted patients within 7 days; additional details are provided in eTable 10 in Supplement 2.

### Outcomes

The 90-day mortality included 26 of 399 patients (6.5%) in the usual care arm, 18 of 204 (8.8%) in the RPM-low standard response arm, 7 of 129 (5.4%) in the RPM-high standard response arm, 24 of 383 (6.3%) in the RPM-low enhanced response arm, and 14 of 171 (8.2%) in the RPM-high enhanced response arm. At least 1 readmission occurred in 151 of 399 patients (37.8%) in the usual care arm, 81 of 204 (39.7%) in the RPM-low standard response arm, 57 of 129 (44.2%) in the RPM-high standard response arm, 143 of 383 (37.3%) in the RPM-low enhanced response arm, and 62 of 171 (36.3%) in the RPM-high enhanced response arm. Integrating survival and days without readmission, the median postdischarge home days were similar across arms: 90 (IQR, 83-90) for the usual care, 90 (IQR, 84-90) for the RPM-low standard response, 90 (IQR, 85-90) for the RPM-high standard response, 90 (IQR, 83-90) for the RPM-low enhanced response, and 90 (IQR, 84-90) for the RPM-high enhanced response arms (Table 2 and eFigures 3 and 4 in Supplement 2).

**Table 2.** Summary of Postdischarge Home Days, Mortality, and Readmissions

Outcome	Treatment arm, No. (%)					
	Total (N = 1286)	Usual care (n = 399)	Individual care model			
			RPM-low standard response (n = 204)^a^	RPM-high standard response (n = 129)	RPM-low enhanced response (n = 383)	RPM-high enhanced response (n = 171)
Postdischarge home days						
Median (IQR)	90 (84-90)	90 (83-90)	90 (84-90)	90 (85-90)	90 (83-90)	90 (84-90)
Value						
−1	89 (6.9)	26 (6.5)	18 (8.8)	7 (5.4)	24 (6.3)	14 (8.2)
0-29	6 (0.5)	0	0	0	4 (1.0)	2 (1.2)
30-59	38 (3.0)	16 (4.0)	7 (3.4)	5 (3.9)	10 (2.6)	0
60-89	388 (30.2)	116 (29.1)	59 (28.9)	49 (38.0)	113 (29.5)	51 (29.8)
90	765 (59.5)	241 (60.4)	120 (58.8)	68 (52.7)	232 (60.6)	104 (60.8)
Death						
Time to death, median (IQR), wk	5.9 (3.4-9.0)	4.6 (2.4-7.0)	5.1 (3.9-8.2)	6.9 (3.3-8.7)	7.3 (5.5-9.5)	5.9 (3.2-7.7)
Readmission						
None	792 (61.6)	248 (62.2)	123 (60.3)	72 (55.8)	240 (62.7)	109 (63.7)
1	289 (22.5)	84 (21.1)	52 (25.5)	40 (31.0)	77 (20.1)	36 (21.1)
2	117 (9.1)	35 (8.8)	18 (8.8)	14 (10.9)	36 (9.4)	14 (8.2)
≥ 3	88 (6.8)	32 (8.0)	11 (5.4)	3 (2.3)	30 (7.8)	12 (7.0)
Total readmission days, median (IQR)^a^	7 (3-17)	8 (4-21)	6 (4-14)	5 (2-17)	8 (4-20)	7 (3-13)

Abbreviations: RPM, remote patient monitoring.

^a^ Readmission days were calculated as the total number of days readmitted, among patients readmitted at least once, to day 90.

**Figure 2.**
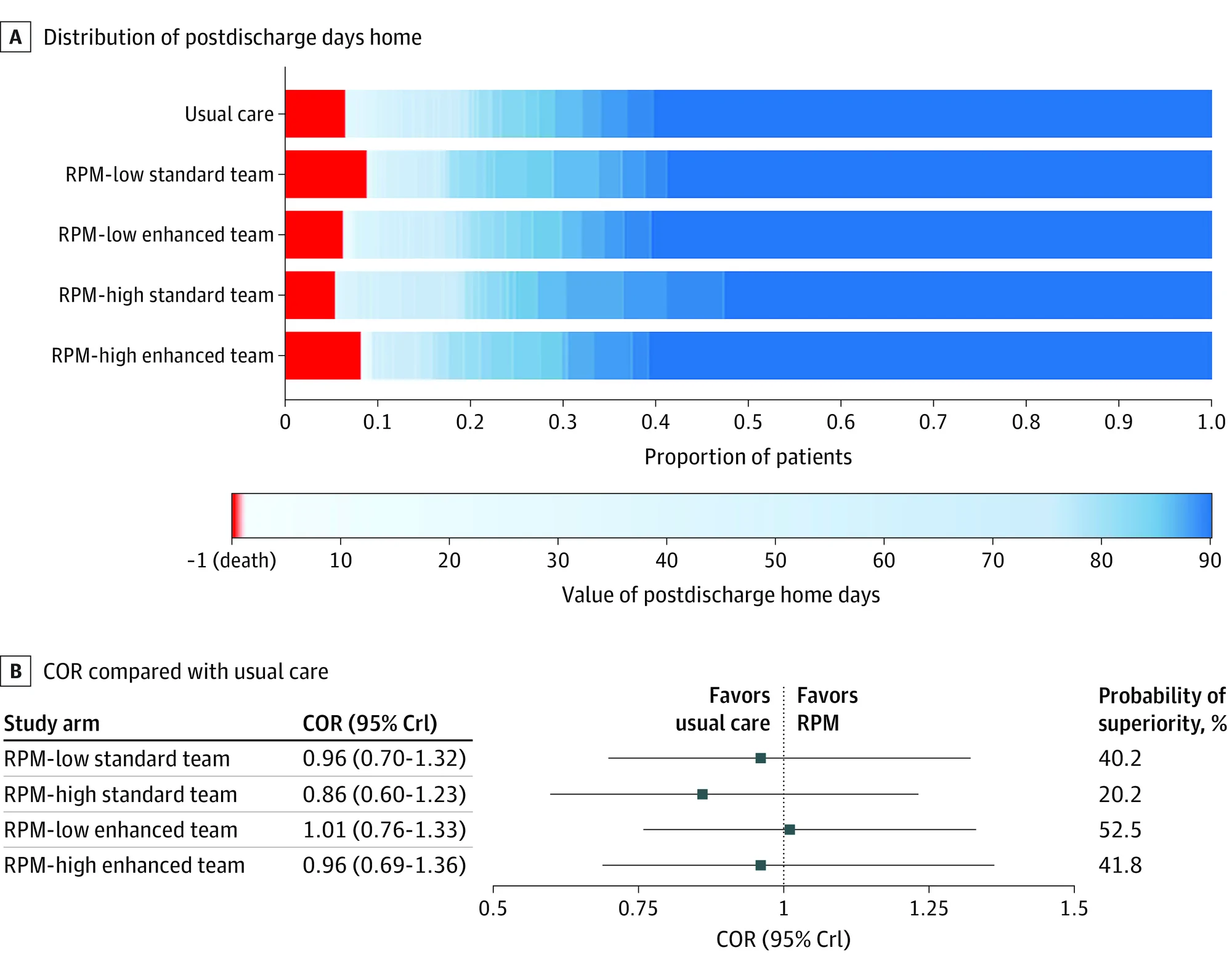
Bar Graph Showing Distributions of Postdischarge Home Days and Forest Plot Showing Cumulative Odds Ratios (CORs) for Each Intervention Arm Postdischarge home days distributions are represented as a horizontally stacked cumulative component bar graph by study group. CORs and the probability of superiority for each study arm compared with usual care are shown as a forest plot. RPM indicates remote patient monitoring.

Compared with usual care, the COR for postdischarge home days was 0.96 (95% CrI, 0.70-1.32) for the RPM-low standard response arm, 0.86 (95% CrI, 0.60-1.23) for the RPM-high standard response arm, 1.01 (95% CrI, 0.76-1.33) for the RPM-low enhanced response arm, and 0.96 (95% CrI, 0.69-1.36) for the RPM-high enhanced response arm, yielding probabilities of superiority ranging from 20.2% to 52.5% (Figure 2). The relationship between CORs and absolute reduction in the proportion who stayed at home and were not readmitted over 90 days is shown in eFigure 5 in Supplement 2. There were no deviations from the assumption of proportional effects across the home-days scale, and each intervention showed effectiveness across the full range (eFigure 6 in Supplement 2). In prespecified analyses combining the RPM intensity and clinical response groups, the CORs were 0.99 (95% CrI, 0.77-1.27) in the RPM-low arms, 0.91 (95% CrI, 0.68-1.23) in the RPM-high arms, 0.92 (95% CrI, 0.69-1.23) in the standard response arms, and 0.99 (95% CrI, 0.77-1.28) in the enhanced response arms, yielding probabilities of superiority ranging from 27.8% to 47.9% (eFigure 7 in Supplement 2). No significant differences in secondary outcomes (eTable 11 and eFigures 8 and 9 in Supplement 2) and sensitivity analyses (eTable 12 in Supplement 2) were observed.

### Heterogeneity of Treatment Effects Across Subgroups

The probability of a differential treatment response by subgroup was high for age and discharge location, ranging from 97.1% to 99.9% (eTable 13 in Supplement 2), and below 95% for subgroups defined by sex, race, chronic conditions, comfort with technology, health literacy, area deprivation index, diagnoses, illness severity, estimated likelihood of enrollment in remote monitoring, and enrollment during COVID-19 surges for both the standard and enhanced response teams.

**Figure 3.**
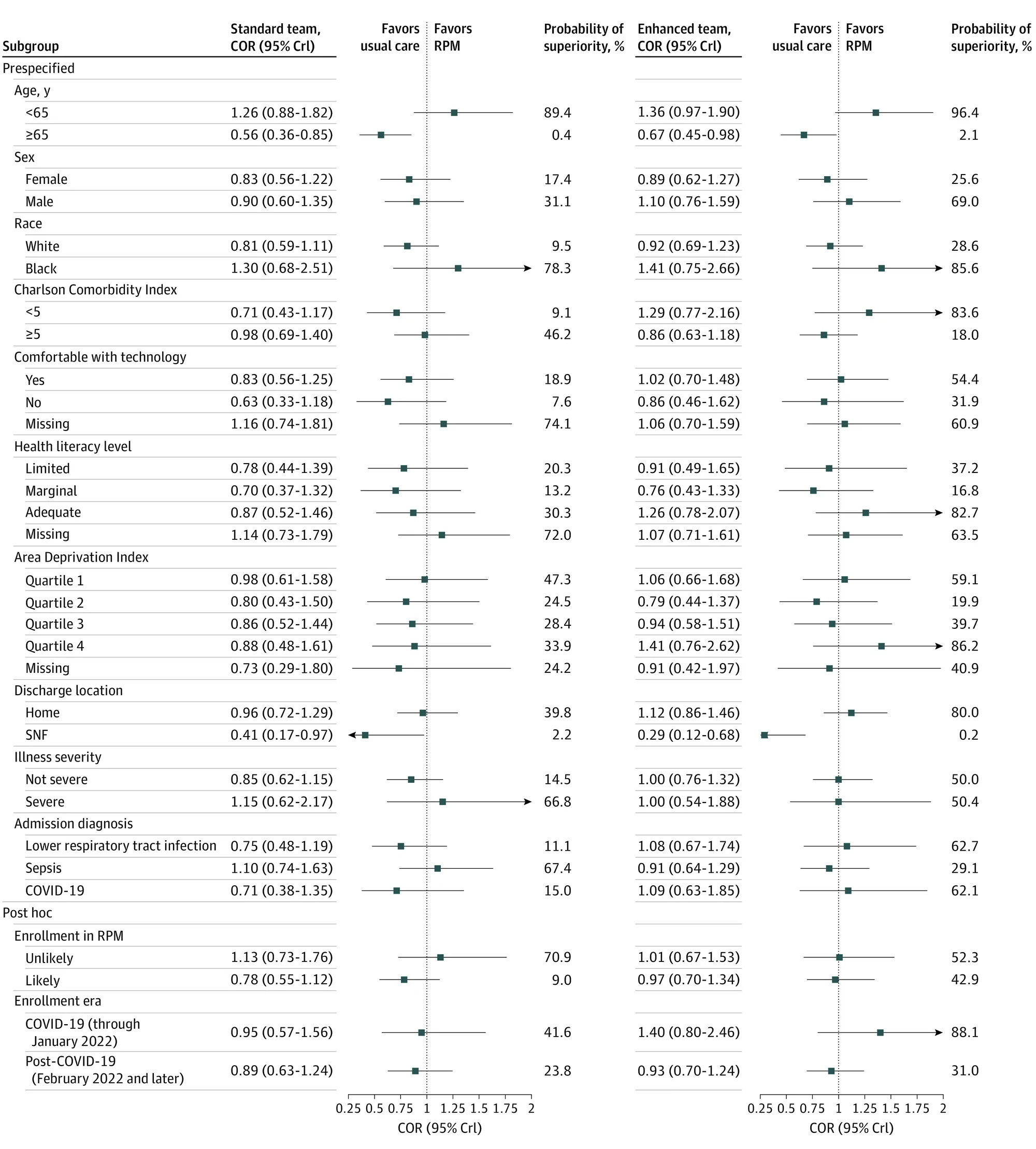
Forest Plot Showing Cumulative Odds Ratios (CORs) and Posterior Probabilities of Superiority for Each Intervention Arm Compared With Usual Care Estimated From the Primary Analysis Model for Subgroups RPM indicates remote patient monitoring.

Among patients 65 years and older (572 [44.5%]), arms with the standard and enhanced response teams had fewer home days compared with usual care, with CORs of 0.56 (95% CrI, 0.36-0.85) and 0.67 (95% CrI, 0.45-0.98), respectively, yielding 99.6% and 97.9% probabilities of inferiority, respectively (Figure 3). At least 1 readmission occurred in 69 patients (47.9%) among the standard response arms and 95 (38.6%) among the enhanced response arms, while only 54 (29.7%) patients in the usual care arm who were 65 years and older were readmitted (eTable 14 in Supplement 2). In patients younger than 65 (714 [55.5%]), no differences were observed, and the CORs were 1.26 (95% CrI, 0.88-1.82) for the standard response arms and 1.36 (95% CrI, 0.97-1.90) for the enhanced response arms, yielding 89.4% and 96.4% probabilities of superiority, respectively. Readmission rates at 90 days included 69 (36.5%) among the standard response arms, 110 (35.7%) among the enhanced response arms, and 97 (44.7%) in the usual care arm. Similarly, among patients discharged to a skilled nursing facility before randomization, both standard and enhanced response arms had fewer home days compared with the usual care arm. The CORs were 0.41 (95% CrI, 0.17-0.97) for the standard response arms and 0.29 (95% CrI, 0.12-0.68) for the enhanced response arms, yielding 97.8% and 99.8% probabilities of inferiority. Readmission rates in this group included 12 patients (42.9%) in the standard response arms, 19 (41.3%) in the enhanced response arms, and 9 (25.0%) in the usual care arm (eTable 15 in Supplement 2). In contrast, no differences were observed for the standard and enhanced response arms among patients who were discharged home from the hospital, with CORs of 0.96 (95% CrI, 0.72-1.29) and 1.12 (95% CrI, 0.86-1.46), respectively, yielding 39.8% and 80.0% probabilities of superiority. Readmission rates were similar across intervention arms: 126 patients (41.3%) in the standard response arms, 186 (36.6%) in the enhanced response arms, and 142 (39.1%) in the usual care arm. Age and discharge location were weakly correlated (Pearson correlation, 0.17). Additional outcomes, including mortality, emergency department visits, and use of hospice services, are reported by intervention arm in eTables 14 and 15 in Supplement 2.

### Qualitative Results

In the qualitative analysis, interviewee demographic characteristics (n = 84) were similar to those in the analysis cohort (eTable 16 in Supplement 2). Related to engagement, most patients believed that remote monitoring technology was easy to use; however, that belief did not necessarily result in engagement as patients noted multiple issues, including remembering to complete their questionnaires and being frustrated by the standardized nature of the monitoring and call center responses. Related to impact, some respondents appreciated the reassurance knowing they were being monitored by a qualified health professional, even though they did not directly interact with the monitoring team. For those who triggered an alert, follow-up was reported to be limited to referrals to primary care or the emergency department, with some suggestions for symptom management. Several patients reported a perceived lack of interpersonal connection and impact, as they had limited or no interaction with the call center. Quotations are provided in eTable 17 in Supplement 2.

## Discussion

In this RCT in patients hospitalized for sepsis and lower respiratory tract infection, our remote monitoring approaches did not increase the days spent at home after discharge. Although none of the interventions were effective, findings suggest heterogeneity in treatment response in prespecified patient subgroups. In patients 65 years and older, remote monitoring reduced days spent at home and increased readmission rates compared with usual care. These findings suggest that the CMS should reassess the role of remote therapeutic monitoring in reducing readmissions and underscore the value of tailoring remote monitoring in post–acute care for serious infections.

Although protocol fidelity was high, patient engagement varied, consistent with the findings of a recent trial^8^ that tested the effectiveness of a navigator-led telehealth intervention as well those of prior remote monitoring studies,^48^ despite multiple strategies to enhance participation. The effectiveness of remote monitoring was similar among the subgroups that had high and low probability of engagement in remote monitoring, indicating that lack of engagement did not explain the negative results of the trial.^7,8,48^ Our findings highlight the challenges of implementing and scaling remote monitoring across health systems and among patients recovering from serious illness who are managing complex care needs, including medications, follow-up appointments, and ongoing symptoms.

The lack of effectiveness was consistent for primary and secondary outcomes and was echoed in qualitative interviews: Patients generally found remote monitoring reassuring but questioned its ability to resolve their problems. We were powered to detect an effect size corresponding to a 9% increase in the probability of surviving to 90 days without readmission. Despite a reduced sample size and lower-than-expected readmission and mortality rates, statistical power was largely preserved to detect differences of this magnitude. While the median number of home days was high, one-third of participants were readmitted. The CORs captured the overall probability of experiencing the discrete outcomes of death or readmission at each time point during 90 days, allowing us to assess differences across the full distribution of home days rather than at a single median value.

Among patients 65 years and older and those discharged to a skilled nursing facility, remote monitoring reduced home days, whereas younger patients and those discharged directly home showed a trend toward more home days, underscoring the need for tailoring remote monitoring. Our interventions closely followed most of the CMS guidance for remote monitoring, with an important goal of reducing readmissions, a long-standing priority at CMS. However, in older adults, remote monitoring may have increased readmissions, potentially by generating alerts that led clinical teams to send patients back to the hospital. Alternatively, it may have provided reassurance, as evidenced by the qualitative interviews, and improved access. Thus, our findings highlight the need for CMS to reevaluate the role of remote monitoring in post–acute care for serious infections.

Usual care included structured telephone support based on the Agency for Healthcare Research and Quality Re-engineered Discharge Toolkit, with calls during the first week after discharge to assess status, review instructions, reconcile medications, and confirm follow-up. In addition to this enhanced discharge support, primary care follow-up was high in the usual care arm, with 42.5% of patients seen within 7 days and 78.9% within 30 days.^49^ This level of timely outpatient engagement likely exceeded routine practice and addressed many issues targeted by remote monitoring, making additional benefit difficult to detect. Thus, the null findings should not be interpreted to mean that patients recovering from sepsis or lower respiratory tract infection do not require post–acute care support.

### Strengths and Limitations

This study has several strengths. To our knowledge, it is among the largest RCTs of remote monitoring in patients with serious infections and was implemented in alignment with CMS guidance. We used an adaptive design, an efficient approach to simultaneously evaluate multiple complex interventions. Randomization and the inclusion of all eligible patients in the intention-to-treat analysis, regardless of level of engagement, helped minimize confounding and selection bias. Last, the use of a patient-centered primary end point,^32,33,34,35^ combining survival and time at home, provided a meaningful measure of recovery and the integration of qualitative data helped contextualize quantitative findings.

The study also has some limitations. First, the trial was conducted in a single health system, which may limit generalizability, although a small subset outside the UPMC network yielded similar results. Second, due to the nature of RPM, an internet-connected device was required for participation, and 717 potentially eligible patients were excluded due to not owning a suitable device. However, smartphone ownership continues to rise across all demographic groups, including older adults, low-income individuals, and rural populations,^13^ supporting the future value of smartphone-enabled interventions. Third, we assessed symptom-based monitoring and not remote physiologic monitoring, and we did not conduct in-person home visits or evaluate the hospital-at-home model, which may limit our understanding of care delivery and patient experience in home-based settings. Fourth, engagement with the intervention was moderate (59.6%). Although consistent with engagement rates in comparable trials,^7,8,48^ this may have limited our ability to detect the intervention’s full effect. However, in a post hoc analysis restricted to participants who engaged in remote monitoring, treatment estimates remained similar.

## Conclusions

In this RCT of patients hospitalized with sepsis or lower respiratory tract infection, remote therapeutic monitoring did not increase days at home after discharge. Subgroup analyses showed heterogeneity of effect; among adults 65 years or older, remote monitoring reduced days at home and increased readmissions. These findings support reevaluating and tailoring remote monitoring in post–acute care following sepsis and lower respiratory tract infection to support further alignment with patients’ needs and desire for personalized monitoring.

## Notes

Supplement 1.
Trial Protocol and Statistical Analysis Plan

Supplement 2. **eMethods.** Remote Patient Monitoring, Qualitative Analyses, and Post Hoc Subgroup Analyses **eFigure 1.** Number of Patients Randomized to Each Arm **eFigure 2.** Number of Patients Randomized Over Time **eFigure 3.** Cumulative Probability Density Plots for Postdischarge Home Days by Intervention Arm **eFigure 4.** Histograms of Postdischarge Home Days Outcomes by Intervention Arm **eFigure 5.** Relationship Between Cumulative Odds Ratios and Absolute Differences in PDHDs **eFigure 6.** Sensitivity Analysis of the Dichotomized Categories of PDHD **eFigure 7.** Marginal Effects of RPM Intensity and Care Team Compared With Usual Care (UC) on Postdischarge Home Days **eFigure 8.** Adjusted Hazard Ratios for Readmission Accounting for Competing Risk of Death by Intervention Arm **eFigure 9.** Cumulative Incidence of Readmission Accounting for Competing Risk of Death by Intervention Arm **eTable 1.** Criteria to Identify Sepsis, Respiratory Tract Infection, and COVID-19 **eTable 2.** Variables and Their Characteristics Used in the Predictive Model to Estimate the Risk of Readmission **eTable 3.** Exclusion Criteria **eTable 4.** Questionnaires Used for Remote Monitoring **eTable 5.** Secondary End Points **eTable 6.** Power Calculations at the Onset of the Trial for Different Scenarios **eTable 7.** Revised Power Calculation Prior to Resizing in November 2023 **eTable 8.** Baseline Characteristics by RPM Intensity and Team Composition **eTable 9.** Fidelity and Engagement of Patients in the Remote Monitoring Pathways or Questionnaires **eTable 10.** Fidelity and Engagement for the 328 Patients Assigned to Enhanced Care Team **eTable 11.** Effects of Study Interventions Compared With Usual Care on Secondary Outcomes **eTable 12.** Results of Subgroup and Sensitivity Analyses **eTable 13.** Probability of Differential Treatment Response Across Prespecified Subgroups **eTable 14.** Mortality, Hospital Readmission, and Hospice Admission Outcomes Stratified by Age Group **eTable 15.** Mortality, Hospital Readmission, and Hospice Admission Outcomes Stratified by Discharge Location **eTable 16.** Characteristics of the Cohort Participating in Qualitative Analyses **eTable 17.** Illustrative Quotes From Qualitative Analyses

Supplement 3.
Data Sharing Statement
