# Errors in Figures, Table, and Supplement

**DOI:** 10.1001/jamanetworkopen.2026.43550

**Published:** 2026-10-16

In the Original Investigation titled “Remote Monitoring Approaches to Reduce Readmissions After Infection and Sepsis: A Randomized Clinical Trial,” published June 11, 2026, the exclusion data were incorrect in Figure 1; the age category and Charlson Comorbidity Index headings were incorrect in Figure 3; the percentages for intensive care unit stay were incorrect in Table 1; the final 5 columns of data were incorrect in eTable 8 in Supplement 2; and footnote “f” required consolidation with footnote “d” in Table 1 and in eTable 8 in Supplement 2. This article has been corrected.^1^
