# Supplementary material for: Remote Monitoring Approaches to Reduce Readmissions After Infection and Sepsis: A Randomized Clinical Trial
Source: JAMA Netw Open. 2026 Jun 11;9(6):e2616641. doi: 10.1001/jamanetworkopen.2026.16641 (PMC13261490; doi:10.1001/jamanetworkopen.2026.16641)
Supplement: Supplement 1. — Trial Protocol and Statistical Analysis Plan [file jamanetwopen-e2616641-s001.pdf]

UPMC Center for High-Value Health Care

Study Title: Adaptive trial to test comparative effectiveness of readmission reduction approaches following infection and sepsis hospitalizations (ACCOMPLISH)

Contract Number: IHS-2019C1-16055

ClinicalTrials.gov Registration: NCT04829188

Date: July 30, 2025 (Version 9)

Funder:

Patient-Centered Outcomes Research Institute (PCORI)
1828 L St. NW, Washington DC 20036
This funding source is not responsible for study design, implementation, interpretation, or dissemination.

Principal Investigators, Research Team, and Study Site: All investigators contributed to the development of the study protocol.

Jatin Dave, MD
Co-Principal Investigator
UPMC Center for High-Value Health Care
Role: Interpretation and dissemination of study findings

Sachin Yende, MD, MS
Co-Principal Investigator
University of Pittsburgh
Role: Study conception, adaptive platform design, scientific oversight

Rana Awdish, MD
Patient Stakeholder Co-Investigator
Henry Ford Health System
Role: Study conception, interpretation and dissemination of study findings

Kelly Williams, MPH, PhD
Co-Investigator
UPMC Center for High-Value Health Care
Role: Operational and implementation oversight

Florian Mayr, MD, MPH
Co-Investigator
University of Pittsburgh
Role: Study conception, adaptive platform design, scientific oversight

Richard Wadas, MD
Co-Investigator
UPMC
Role: Operational and implementation oversight

Derek Angus, MD, MPH
Co-Investigator
University of Pittsburgh
Role: Trial design and execution, dissemination of research findings

Kimberly Rak, PhD, MPH
Co-Investigator
University of Pittsburgh
Role: Qualitative research design, execution, and analysis

Cara Nikolajski, MPH, PhD
Co-Investigator
UPMC Center for High-Value Health Care
Role: Qualitative research design and analysis

Joyce Chang, PhD
Co-Investigator
University of Pittsburgh
Role: Statistician

Primary Research Sites:

UPMC Center for High-Value Health Care
600 Grant Street, 40th Floor
Pittsburgh, PA 15219

UPMC Health Services Division
200 Lothrop Street
Pittsburgh, PA 15213

University of Pittsburgh
Department of Critical Care Medicine
3550 Terrace Street
Scaife Hall, Suite 600
Pittsburgh, PA 15213

Table of Contents:

Research Synopsis 1
Background 2
Significance 3
Objectives 5
Aims 5
Interventions 6
Study Design/Methodology 10
Study Setting 10
Randomization Procedure 10
Study Population 10
Inclusion/Exclusion Criteria 11
Potentially Eligible Patient Review Process 11
Measures 13
Study Duration/Study Timeline 17
Data Collection 17
Sample Size 18
Informed Consent Process 18
Privacy and Confidentiality 19
Risks and Benefits to Participants 19
Compensation for Participation 20
Study Oversight 20
Conflict of Interest 20
Dissemination 20
References 22

List of Common Abbreviations in Protocol:

RPM: Remote Patient Monitoring
SNF: Skilled Nursing Facility
RAR: Response Adaptive Randomization
CER: Comparative Effectiveness Research
STS: Structured Telephone Support

Research Synopsis

Study Population: Adults hospitalized for sepsis or lower respiratory tract infection age 21 and older with medium to high risk of readmission.

Design: A comparative effectiveness study using individual-level response adaptive randomization design along with a pragmatic, mixed-methods approach to compare combinations of five remote patient monitoring (RPM) strategies. Quantitative (e.g. self-report, electronic health record, process) and qualitative (e.g., interviews) data will be collected across multiple time points during the study period.

Sample Size: We expect to enroll 1,281 individuals.

Study Duration: The contract period begins August 3, 2020 and concludes August 31, 2026.

Background

Readmissions after sepsis or pneumonia exact an enormous toll on the health and quality of life of individuals and pose significant challenges for the health care system. Approximately one in five Medicare beneficiaries discharged from the hospital are readmitted within 30 days and a third in 90 days,[1] and older adults with multimorbidity are at higher risk.[2] Returning to the hospital after an index admission is shown to worsen patients' quality of life and increase their risk of healthcare-acquired infections,[3] despite the fact that many of these readmissions are avoidable.[4]

Individuals hospitalized for sepsis or pneumonia are particularly at risk for readmission and poor health outcomes. Among 14 million readmissions in the National Readmissions Database, sepsis and pneumonia (~3 million cases annually in US) were leading causes of readmissions.[5] Readmissions following sepsis and pneumonia are more common and associated with higher costs than readmissions due to heart failure, chronic obstructive lung disease (COPD), or myocardial infarction.[1,5-9] Survivors of both conditions experience reduced quality of life, increased mortality and morbidity,[10-12] and cognitive impairment and functional limitations.[13-15] Despite readmissions, one in three patients do not survive the year following sepsis or pneumonia, and those who do survive often experience persistent health deficits.[6-8,10,16] In a subset of hospitals owned by UPMC, a large integrated delivery and finance system in Pennsylvania, all-cause 30-day readmission rates for individuals with sepsis or pneumonia (23%) exceed the overall average (12%).

Further, readmissions following sepsis or pneumonia are often preventable. Up to nearly half of readmissions after sepsis can be attributed to preventable causes[17] and the preventability of readmission after pneumonia is recognized by the Centers for Medicare and Medicaid Services' (CMS) Hospital Readmission Reduction Program as an indicator of quality of care.[18] Most patients hospitalized with sepsis or pneumonia are >65 years of age and have multimorbidity.[19,20] Two-thirds of readmissions following sepsis or pneumonia were due to infection (~40%) and cardiovascular (15%) and respiratory disease (10%).[21] In theory, adequately monitoring patients, detecting early signs of deterioration, and triaging them to appropriate care would decrease unnecessary care utilization and increase patient stability. This is the premise of several widespread interventions such as in-hospital rapid response teams, post-discharge remote patient monitoring (RPM) for heart failure, and primary care-led monitoring in the community.[22,23]

However, there are critical gaps in knowledge regarding how comprehensively we should remotely monitor patient symptoms and types of health care teams best suited for responding to alerts to maximize patient time at home. The standard of care for patients with multimorbidity is to provide some level of care coordination or care provision after discharge, as we know it works. There is a large body of evidence demonstrating that post-acute care models as a whole reduce the risk of an individual returning to the hospital after discharge.[24] Among these, more complex interventions that leverage multiple components and multiple care providers have greater effect on maintaining patients at home longer after discharge.[24] Specific successful model components include care coordination/care management activities and patient self-management education.[25,26]

A key priority for the health care system is understanding how to effectively deploy the post-acute workforce in the community.[27,28] Structured Telephone Support (STS), or telephonically connecting with patients post-discharge to assess health status, reconcile medications, and provide patient education, is a well-studied, effective strategy. Within UPMC, deployment of STS for several years across a wide population has successfully reduced readmissions. However, STS may be insufficient for patients at higher risk of readmission.[29] For more complex patients, RPM platforms have extended the reach of

providers by regularly collecting patient-reported data on vital signs and symptoms, allowing early recognition of worsening disease and deploying interventions promptly.[30] Provider monitoring of symptom exacerbation is important because patients alone are unreliable in reporting worsening symptoms.[26,31] RPM platforms have received FDA approval, reduced readmissions in multiple observational and randomized controlled studies, and are routinely used in clinical care.[26,32] While patient acceptability of RPM is high, RPM platforms typically monitor one disease or condition (e.g., heart failure exacerbation in patients hospitalized for heart failure). Given that multimorbidity is associated with hospitalization for serious infection and readmissions are often due to worsening of underlying chronic conditions, utilizing RPM to assess multiple disease states could be more successful in early identification and intervention. However, the optimal post-acute care strategy for patients with multimorbidity is unknown.

Additionally, more information about the spectrum of services delivered within and alongside RPM is critical to effectively meeting patients' needs. There is conflicting evidence about whether models that couple RPM with an alert-triggered provider response (Standard Team) or a more intensive patient engagement model (Enhanced Team) are more effective,[33-35] the reason for which may be that studies often fail to define specific intervention components.[36] Several studies show that multidisciplinary teams that periodically conduct in-person or virtual home visits and specialize in chronic disease management, care coordination, and patient education about self-management may increase time spent at home.[37,38] However, this approach is resource intense and may be difficult to scale across large geographic areas.[39] Noting that RPM findings are often confounded by additional in-home care received by both intervention and control groups, one study measured the effect of RPM versus usual outpatient care in a sample of patients not receiving concurrent home-based care and showed decreased emergency department (ED) utilization and hospital length of stay and increased quality of life among the group receiving RPM.[40] The study of RPM without explicit investigation of the model of care into which RPM is built is a critical gap in the comparative effectiveness literature.[41]

Significance

UPMC's integrated delivery and finance system offers a unique environment for conducting a patient-centered comparative effectiveness research (CER) study that will rapidly increase the ability of patients, caregivers, and other stakeholders to make informed decisions about post-acute care after sepsis or pneumonia.[30] Building upon years of collaborative work involving care models for individuals with multimorbidity, stakeholder engagement, pilot testing, and existing research, this study will provide much-needed information about how best to support individuals in their homes after discharge.

UPMC's existing technology infrastructure is well-suited to support the proposed work. Our RPM platform allows for synchronous and asynchronous communication between patients and providers using a variety of devices including patient-owned smart phones, tablets, and computers to monitor symptom exacerbations and avoid the potentially preventable ED visits and hospitalizations.[42] This technology, which is currently used to engage with and monitor patients via modules that target individuals with conditions such as COPD, heart failure, cancer, infectious disease, and frailty, can easily be adapted for study purposes. Currently, over 500 hospitals have contracts for using this same technology to provide services for improving the health of a variety of patient populations. In addition, care management databases, smart phone applications, and mobile health products developed through the UPMC Insurance Services Division (ISD) Consumer Innovation Department are all designed to expand our ability to provide, more efficiently, comprehensive services to some of our most vulnerable patients.

RPM supports multiple care models within UPMC. The standard of care when using RPM is to code patient-reported data into alert levels (low, medium, high). Medium and high alerts are sent to a nurse-led call center, where staff contact the member or provider and recommend appropriate action such as scheduling a follow-up appointment or visiting the ED. RPM is also used to support an enhanced care team led by a certified registered nurse practitioner (CRNP) with prescribing authority. This team delivers home-based or virtual care management, which includes advanced care planning and goals of care discussions and triages and manages symptoms reported via RPM. Internal evaluation data show that members touched by this team have reduced unplanned care and increased days at home.

From a technological standpoint, the use of telemedicine, including RPM, is rapidly expanding across the US. Several recently released industry reports predict high growth in telemedicine reimbursement and system investments.[43-45] Medicare reimbursement policies are quickly evolving to support models of care that use remote monitoring to manage chronic conditions.[46] Major insurers across the U.S. offer RPM and all have telehealth reimbursement policies, with varying conditions about where it needs to be delivered.[47,48] Nearly 1800 hospitals across the US currently use apps to support RPM[49] and 2 in 3 providers plan to use RPM in the future.[50] Finally, RPM is the standard of care for several chronic conditions.[51]

Nurse practitioners (NPs) already play a major role in providing telemedicine care in the US. In an analysis of the Minnesota All Payer Claims Database nearly half (46%) of all 2015 telemedicine visits were delivered by NPs.[52] Multidisciplinary palliative care teams that include NPs are used throughout the US health care system,[47,53] and 67% of US hospitals with 50+ beds have palliative care teams, an increase from 53% in 2008.[54] Among providers conducting home-based care, NPs form the largest provider group and provide care across the largest geographic area.[55]

Training for NPs in advanced illness care using telemedicine is scalable. NPs can obtain academic certificates in palliative care through accredited higher education institutions (generally averaging 150 hours of study[56,57]) and through in-person or web-based courses and trainings offered through various organizations, such as Vital Talk[58] and Defining Hope.[59] Thus, if palliative care NP-led programs are effective, this program can be generalized to other healthcare systems. As noted by Lazur et al., the rapid increase in use of telehealth technology "has outpaced the release of research about this model of care."[60] This research is optimally timed to provide relevant research that health systems can leverage as they implement telehealth technology to support critically ill populations.

Our research will help to efficiently and effectively answer questions that are critical to patients, caregivers, providers, and payers regarding how to ensure individuals remain in their homes after discharge. Studies consistently show that patients prefer to maximize the time spent at home instead of a healthcare facility, especially during the last six months of life.[27,61-63] Stakeholder feedback during the study design process clearly articulated a need to increase the efficiency of traditional research, ensuring that the health care system has access to quality evidence to learn quickly what works best for whom. Therefore, we propose to use an adaptive platform trial, which uses a common platform to enroll all patients with the condition of interest (e.g., sepsis or pneumonia) and tests multiple interventions simultaneously.[64,65] The adaptive design incorporates rules to modify treatment arm randomization ratios over time by updating our Bayesian statistical model as new patients are accrued and preferentially enrolling to arms for which evidence of benefit is the strongest. This approach requires a smaller sample size (and potentially shorter study timelines) compared to a traditional trial and reduces the likelihood of exposing patients to therapies that are ineffective or harmful. The rules to change randomization probabilities to individual arms are written prior to start of the trial. The I-SPY 2[66] trial is the best known example of an adaptive platform trial and this design is also being used for other

conditions, including glioblastoma multiforme,[67] pancreatic cancer,[68] Parkinson's disease,[69] and cystic fibrosis.[70] Therefore, we propose to test multiple combinations of RPM and care team interventions to determine if any of them are superior to STS (**Aim 1**).

Ensuring that the most resource-intensive interventions are directed toward patients most likely to benefit is a critical challenge for the health care system as it strives to care for increasingly complex populations with limited resources.[28,71,72] Although precision medicine has traditionally focused on biomarkers (e.g., genomic markers), we will focus on patient-level clinical, social, and environmental factors to tailor service delivery (precision population health) to patients who are most likely to benefit (**Aim 2**). In addition to conducting traditional exploratory subgroup analyses testing one variable (or risk prediction score) at a time using a regression-based approach, we propose a novel machine learning-based algorithm for several reasons. First, we can model many factors simultaneously, leaving the door open for complex interactions to emerge from the data in a flexible manner. Second, the machine-learning models (e.g., causal forest) do not require assumptions regarding the relationship between a covariate and treatment effectiveness. Third, we can predict treatment effectiveness at the patient-level, allowing us to develop personalized strategies.

Objectives

This goal of this study is to answer critical questions about how to best design RPM programs to maximize home-days and minimize readmissions for patients discharged after sepsis or lower respiratory tract infection. Moreover, this study seeks to provide valuable information about how RPM programs may benefit patients discharged after other diseases. This information will be beneficial to patients, providers, payers, health systems, and community organizations seeking to maximize days spent at home.

Aims

There is uncertainty regarding how comprehensively we should remotely monitor patient symptoms and which health care team is best suited to triage care in the post-acute setting. While STS is an effective strategy, telemedicine-based care models might be a better option for patients at higher risk of readmission. These models allow at-home surveillance using a RPM platform, early detection of worsening of health, and deployment of an intervention once an alert is generated. RPM is typically used to monitor and prevent readmissions for a single disease (e.g., heart failure). This low-intensity RPM (**RPM-Low**) approach may not be effective in patients discharged after sepsis and pneumonia where readmissions are frequently due to worsening of sub-clinical disease, and surveillance for multiple conditions (**RPM-High**) may be necessary. An important element of RPM is deploying interventions once an alert has been received. Typically, a physician or a nurse responds to alerts (**Standard Team**), but dedicated nurse-practitioner (NP)-led multidisciplinary teams may be more effective (**Enhanced Team**).

We will randomize patients to five arms: 1. STS; 2. single-condition RPM with standard provider-led responses to alerts (RPM-Low + Standard Team); 3. single-condition RPM with NP-led multidisciplinary team patient engagement (RPM-Low + Enhanced Team); 4. multi-condition RPM with standard provider-led responses to alerts (RPM-High + Standard Team); and, 5. multi-condition RPM with NP-led multidisciplinary team patient engagement (RPM-High + Enhanced Team). The RPM care models tested in this study may not be more effective than STS or more than one care model may be effective in the broad group of patients. In either scenario, identifying subgroups for whom these care models are most effective is consistent with "precision population health," which targets interventions based on clinical, social, and environmental determinants of disease.

Aim 1: To compare the effectiveness of care models that vary based on intensity of RPM and team composition to increase the number of days spent at home among patients discharged home after sepsis or pneumonia.

Aim 1a. To determine if patients randomized to RPM-Low + Standard Team, RPM-Low + Enhanced Team, RPM-High + Standard Team, and RPM-High + Enhanced Team interventions have higher home days compared to STS.

Aim 1b. To determine the optimal care model (listed in Aim 1a) in terms of the number of days spent at home.

Aim 2: To determine the patient subgroups for whom individual RPM care models are most effective.

Aim 3: To understand contextual factors related to intervention adoption, satisfaction, effectiveness, and sustainability.

Aim 3a. To understand patients' and intervention providers' perceptions of contributors to intervention acceptability, value, and satisfaction.

Aim 3b. To determine in what ways the interventions impact the primary outcomes of post-discharge home days as well as secondary outcomes, including functional status and quality of life.

Aim 3c. To assess facilitators and barriers to intervention acceptability, feasibility, fidelity and sustainability.

Aim 3d. To explore considerations and strategies that can be used to support scaling of the interventions for other patient populations and health care settings.

Interventions

All intervention components are currently in use by patients with complex care needs and were originally designed to address ongoing care access issues while not compromising the core, evidence-based components of each intervention. The technology components of these interventions will be delivered through and/or supported by UPMC's existing RPM platform, which can enhance symptom monitoring and improve consistency of patient and provider contact.[73] Each intervention is designed to facilitate symptom monitoring, identification of worsening disease, and adherence to follow up care recommendations. RPM interventions will start when the participant returns home, regardless of whether they had a temporary SNF stay between hospital and home or not. Participants will only answer RPM questions while at home. Moreover, the interventions are designed to proactively address these issues through actions of the care teams. Participants and physicians are free to discontinue RPM programs per existing programmatic and clinical protocols if doing so is in the best interest of the patient. Study comparators (1. STS, 2. RPM-Low + Standard Team, 3. RPM-Low + Enhanced Team, 4. RPM-High + Standard Team, and 5. RPM-High + Enhanced Team) are a combination of the evidence-based components described below:

- Structured Telephone Support (STS):[29] Structured telephone support (STS) consists of post-discharge assessment, education, and medication reconciliation delivered telephonically by a health plan case manager, home care as needed, and follow-up with the primary care within seven days post-discharge.
- Low-intensity Remote Patient Monitoring (RPM-Low):[74] Questions are pushed to members multiple times per week for up to 90 days post-discharge. Questions are limited to those checking vital signs that indicate worsening of infection. Patient answers to RPM questions trigger High or Medium alerts, which trigger a response by members of the intervention care team (see below).

- High-intensity Remote Patient Monitoring (RPM-High):[22] Questions are pushed to members multiple times per week for up to 90 days post-discharge. Questions include monitoring vital signs for worsening infection but also ask about factors that would indicate worsening of underlying heart or lung conditions, such as weight gain or shortness of breath. Patient answers to RPM questions trigger High or Medium alerts, which trigger a response by members of the intervention care team.
- Standard Team:[40,75] RPM alerts are screened by a nurse-staffed call center. Nurses determine whether emergency care is needed. If not, nurses contact the patient and/or the patients' PCP or specialist to coordinate care and ensure timely follow-up.
- Enhanced Team:[25,37,76] RPM alerts are screened by a nurse-staffed call center. Nurses determine whether emergency care is needed. If not, the call center alerts a multidisciplinary care team that is led by a certified registered nurse practitioner (CRNP). CRNPs, who operate in a palliative care role, have prescribing authority and can modify care plans. In addition to reacting to RPM triggers, team members (e.g., CRNP, social workers, nurses) meet with the patient in-person or virtually in the week after discharge and at least twice more in the next 90 days, conduct assessments and a pharmacy review, develop care plans, and complete a POLST (physician orders for life-sustaining treatment) or a PA Advance Directive.

RPM pathway questions

	Category	Question	Audience	Frequency
1	Thermometer	Based on the thermometer, how are you currently feeling (0-10)?<br>0, 1, 2: **<u>SKIP</u> ALL SYMPTOM QUESTIONS**<br>3, 4, 5, 6: **<u>ASK</u> ALL APPLICABLE SYMPTOM QUESTIONS**<br>7-10 *(HIGH ALERT)* **<u>ASK</u> ALL APPLICABLE SYMPTOM QUESTIONS**	All	Twice per week
2	Symptom	Have you had swelling in your legs and ankles in the past 3 days?<br>Yes *(MEDIUM ALERT)*<br>No	RPM High only	Twice per week
3	Symptom	Have you had a worsening cough or chest pain in the past three days?<br>Yes *(MEDIUM ALERT)*<br>No	RPM High only	Twice per week
4	Symptom	Are you having pain?<br>Yes<br>No<br>**If yes**: Is your pain worse than usual?<br>Yes *(MEDIUM ALERT)*<br>No<br>Pain Health Tip	All	Twice per week
5	Symptom	Are you having problems breathing?<br>Yes<br>No<br>**If yes**: Is your breathing worse than usual?<br>Yes *(MEDIUM ALERT)*<br>No<br>Shortness of Breath Health Tip	All	Twice per week
6	Symptom	Do you have nausea or vomiting?<br>Yes<br>No<br>**If yes:** Are you unable to eat or drink because nausea and/or vomiting?<br>Yes *(MEDIUM ALERT)*<br>No	All	Twice per week
7	Symptom	Do you have problems moving your bowels?<br>Yes<br>No<br>**If yes:** Has it been more than 3 days since you had a bowel movement<br>or is your stool hard to pass?<br>Yes *(MEDIUM ALERT)*<br>No	All	Twice per week
8	Symptom	Do you feel lightheaded or faint?<br>Yes *(MEDIUM ALERT)*<br>No	All	Twice per week
9	Symptom	Have you fallen in the past week?<br>Yes *(MEDIUM ALERT)*<br>No	All	Weekly

10	Symptom	Do you have fever or chills?<br>Yes *(MEDIUM ALERT)*<br>No	All	Twice per week
11	Symptom	Do you feel like you must go to your doctor’s office or emergency room in the next few days?<br>Yes *(MEDIUM ALERT)*<br>No	All	Twice per week
12	Medication	Do you have questions about your medicines or medical conditions?<br>Yes *(MEDIUM ALERT)*<br>No	All	Twice per week
13	Medication	Have you missed any doses of your antibiotic in the past three days?<br>Yes *(MEDIUM ALERT)*<br>No	All	Twice per week
14	Medication	Have you started a new medicine in the last month?<br>Yes *(MEDIUM ALERT)*<br>No	All	Monthly
15	Help	Do you have someone who can help you with transportation bill paying and shopping?<br>Yes<br>No *(MEDIUM ALERT)*	RPM High Enhanced<br>RPM Low Enhanced	Monthly
16	Help	Do you have problems caring for yourself at home?<br>Yes *(MEDIUM ALERT)*<br>No	RPM High Enhanced<br>RPM Low Enhanced	Monthly
17	PHQ	PHQ 2/9		Monthly
18	FAMCARE	FAMCARE P13		Monthly
	Video	Video about advanced care planning		One time
	Video	Video about choosing a health agent		One time

Study Design/Methodology

We have chosen an individual-level response adaptive randomized platform study design along with a pragmatic, mixed-methods approach to compare five RPM program configurations for delivering post-acute monitoring to patients with sepsis or lower respiratory tract infection across western and central Pennsylvania.

Study Setting

Participants will be recruited from the hospitals listed below. All hospitals are located in Pennsylvania. Historical data suggests that recruiting from these hospitals will allow us to reach our target sample size. For detailed recruitment methods, please refer to the Recruitment Plan

**Recruitment Sites**		
Armstrong County Memorial Hospital	UPMC Hamot	UPMC Northwest
Meadville Medical Center	UPMC Horizon	UPMC Passavant
Conemaugh Memorial Medical Center	UPMC Jameson	UPMC Presbyterian
St. Clair Hospital	UPMC Magee Women's Hospital	UPMC Shadyside
UPMC Altoona	UPMC McKeesport	UPMC St. Margaret
UPMC East	UPMC Mercy	Washington Hospital
Heritage Valley Beaver	Heritage Valley Sewickley	

Randomization Procedure

The study will use web-based randomization to one of the five care models for those individuals who consent to participate in the study. Once a research coordinator determines eligibility, obtains informed consent, and the patient is discharged to home while maintaining eligibility, he or she will enter in key identification information, and the system will then generate a Study ID (numeric identification number) along with assignment to an intervention arm. Randomization will initially be fixed with 30% of patients randomized to STS and the remaining 70% of patients randomized 1:1:1:1 between the four RPM care models. After 250 patients have been randomized and followed for 90 days, the first interim analyses will be conducted and RAR will begin and continue to be updated every three months. During the RAR phase, the randomization to STS will remain fixed to be 30% of patients and the remaining 70% of patients will be randomized to one of the four RPM models preferentially based on the probability that each RPM care model is optimal.

While the research coordinators will see each assigned intervention arm per the randomization process, they will be blinded to overall changes in treatment allocation rates. Other study team members, including Principal Investigators, Co-Investigators, statistical analysts, and database administrators will also be blinded to treatment allocation rates during the course of data collection.

Study Population

Our study population will consist of patients who are UPMC Health Plan members or have Medicare Fee-for-Service insurance age 21 and older hospitalized with a primary diagnosis of sepsis or lower respiratory tract infection, who are discharged to home (from the hospital or after a SNF stay of 28 days or fewer), and are at moderate- or high-risk for readmission.

Inclusion/Exclusion Criteria

1. Inclusion criteria
    a. UPMC Health Plan member or patient with Medicare Fee-for-Service insurance
    b. Treated at a prespecified hospital for sepsis or lower respiratory tract infection
    c. Age 21 or older
    d. Medium or high risk of readmission
2. Exclusion criteria
    a. Admitted from setting other than home, independent living, or personal care home
    b. Receiving hospice at time of admission
    c. For Health Plan members: being discharged on/to hospice, Inpatient Rehabilitation, long-term acute care hospital, or assisted living facility
    d. For Medicare Fee-for-Service enrollees: being discharged on/to hospice, Inpatient Rehabilitation, long-term acute care hospital, skilled nursing facility, or assisted living facility
    e. Currently participating in UPMC Advanced Illness Care program
    f. Unable to participate in RPM due to lack of technology/internet connection
    g. Known Pregnancy
    h. Severe, persistent cognitive impairment
    i. PCP disapproves of patient being in remote patient monitoring program
    j. No PCP available to participate
    k. Patients who are discharged from hospital to skilled nursing facility and stay at the skilled nursing facility for greater than 28 days

Unanticipated Enrollment in UPMC Advanced Illness Care Program

The UPMC Advanced Illness Care (AIC) Program is a 6-month palliative care program offered by UPMC Health Plan to members in need of palliative care. Referrals to this program are made both at hospital discharge and during the course of routine outpatient care.

It is rare but possible for a randomized ACCOMPLISH participant to be enrolled into the UPMC Advanced Illness Care (AIC) Program. The Co-Enrollment Workflow is a detailed process designed to prevent this issue; however, co-enrollments are still possible due to organizational complexity and care process variation.

In the event that a randomized ACCOMPLISH participant is also enrolled in the AIC program, the study team will follow a strict intent to treat study design and continue to collect outcomes data per the established timelines and processes. If the number of randomized participants is greater than 10 at the end of all recruitment and data collection, a sensitivity analysis will be performed to determine if the AIC participants have significantly different outcomes than the participants enrolled in the study as planned.

Potentially Eligible Patient Dashboard Identification and Chart Review Process:

Potential participants are initially identified through a dashboard populated daily with inpatient data pulled from an UPMC's electronic health record system. This dashboard shows the medical record number of inpatients who meet some ACCOMPLISH study inclusion criteria such as hospital, age, insurance type, readmission risk score (derived from a predictive model), and pre-specified diagnostic indicators suggestive of sepsis and lower respiratory tract infection (antibiotics ordered for at least two days and at least one culture ordered within the first three days of a hospital stay). The RCs examine the medical records of patients identified on the dashboard for further eligibility criteria including where a patient was admitted from, their planned discharge location (home, SNF, hospice, etc), pregnancy

status, and specific diagnostic indicators of sepsis or lower respiratory tract infection. The table below shows standard criteria found in medical records to use as a guideline determine if an RC can independently deem the patient clinically eligible for the study, or if a clinically-trained PI/Co-I needs to review the patient's chart to confirm clinical eligibility.

Condition	Criteria used by research coordinator
**Lower respiratory tract infection**	**All three criteria were met:**<br>• Received at least two days of antibiotics, which were started within 24 hours of hospital presentation<br>• At least one culture from blood or lower respiratory tract ordered within first three days of hospital presentation<br>• Pneumonia or chronic obstructive pulmonary disease (COPD) exacerbation considered as a potential diagnosis
**Sepsis**	**All three criteria were met:**<br>• Clear source of infection (e.g., urinary tract infection, catheter-related blood stream infection, abscess) OR at least one culture (e.g., blood, urine, lower respiratory tract) ordered within the first three days<br>• Received at least two days of antibiotics, which were started within 24 hours of hospital presentation<br>• At least one criterion for organ dysfunction or notes mentioned "sepsis" or "septic shock":<br>o Elevated serum creatinine and blood urea nitrogen levels (kidney function)<br>o Elevated serum lactate levels<br>o Received vasopressors (e.g., dobutamine, norepinephrine, epinephrine)
**COVID-19**	**All three criteria were met:**<br>• COVID-19 was the primary reason for hospital admission<br>• Presence of respiratory symptoms (e.g., shortness of breath, cough dyspnea)<br>• Prescribed/ordered one of the following during hospitalization:<br>o Dexamethasone (6 mg daily)<br>o One of the following: Remdesivir, Sotrovimab, Bebtelovimab, Paxlovid (nirmatrelvir tablets and ritonavir tablets, co-packaged for oral use), Tocilizumab, Baricitinib<br>o Received vasopressors, high-flow oxygen, invasive or non-invasive mechanical ventilation

Measures

Primary outcome

Post-discharge home days at 90 days: Home days will be calculated as days alive and at home, a composite of mortality and morbidity. We included mortality to ensure that competing risk of death will not affect this outcome. The proposed interventions are likely to have a small effect on time to death in these patients.

Secondary outcomes

Functional Status: will be measured at baseline and 90 days, using the PROMIS Physical Function – for Mobility Aid Users-SF. The Patient-Reported Outcomes Measurement Information System (PROMIS™) provides item banks that offer the potential for PRO measurement that is efficient (minimizes item number without compromising reliability) flexible (enables optional use of interchangeable items), and precise (has minimal error in estimate) measurement of commonly-studied PROs.[77] PROMIS item banks and their short forms provide evidence they are reliable and precise measures of generic symptoms and functional reports comparable to legacy instruments.[78]

Health-related Quality of Life: will be measured at baseline and 90 days, using the Quality of Life Enjoyment and Satisfaction Questionnaire-SF. The Quality of Life Enjoyment and Satisfaction Questionnaire (Q-LES-Q) is a self-report measure designed to enable investigators to easily obtain sensitive measures of the degree of enjoyment and satisfaction experienced by subjects in various areas of daily functioning.[79]

Transition to Hospice: will be assessed via claims data at 90 days to see if member transitioned to hospice care during the course of the study.

Emergent outpatient utilization: will be assessed via claims data at 90 days for a count of urgent outpatient visits for study participants.

Readmissions: will be assessed via claims data at day 7, 30, and 90 for any inpatient admissions after index admission, and whether readmission hospital was the same as index hospital.

Mortality: will be assessed via claims data at 90 days to see if and when the member/participant passes away.

Covariates

We will include variables likely to influence the strength of the relationship between the interventions and outcomes and included variables within domains (patient-level, socioecological, and care delivery and utilization) that have hypothesized associations with the primary and secondary outcomes. These covariates will be the basis for Aim 2 analyses. Finally, we will monitor adherence to pre-established intervention workflows and RPM response rates throughout the study to ensure that intervention fidelity is maintained and can be accounted for in the analyses.

Domain: Patient-Level:

Patient Demographics and clinical characteristics: We include in our baseline survey questions regarding age, race, ethnicity, marital status, and gender. Insurance type and the Charleston Comorbidity index will be pulled from administrative and claims data at baseline. We will ask questions about generalized anxiety disorder, depressed mood and anhedonia, health literacy, and comfort with technology.

Living at home status: We include in our baseline and 90-day survey questions regarding whether a participant lives at home alone or with others. If participants live with others, an additional question to identify their living companion(s) is asked. The participant can identify multiple living companions from a list.

Payer: Based on literature review and the experience of our study investigators, we do not expect large differences in the distributions of post-discharge home days between UPMC Health Plan and MC FFS study participants. However, in the event that participant payer (UPMC Health Plan or MC FFS) is predictive of home day distribution, randomization of study interventions ensures that effectiveness comparisons are guaranteed to remain unbiased toward any particular arm. Thus, our primary analysis model will remain as it is currently stated in the Statistical Analysis Plan.

To maximize oversight the proposed new subgroup (MC FFS), home days distributions of each payer type group will be reported to the DSMB in regular quarterly interim analysis reports.

In addition, we will conduct two types of sensitivity analyses to detect any differences in the two distributions after data collection:

1. We will fit two additional proportional logit models accounting for payer type:
    a. One logit model including a term for payer which will capture proportional shifts in home days across payer groups; and
    b. A second model that will include different intercept terms for the payer type groups. This model will capture non-proportional shifts in home days across both groups.
2. We will explore potential differential effects of RPM care models across payer types. One model will estimate interaction terms between RPM arm and payer type; and another will fit separate proportional logit models to UPMC Health Plan and MC FFS participants.

Domain: Socioecological:

Distance to primary care provider (PCP) office: Distance from participant's home address to the PCP office in miles will be pulled from administrative data at baseline.

Area Deprivation Index (ADI): The ADI will be a composite score of socioeconomic variables to indicate neighborhood socioeconomic status pulled from administrative data at baseline.

Domain: Care Delivery and Utilization:

Non-urgent outpatient care utilization: The count of non-urgent outpatient visits that occur per participant during the observation period (primary and specialty care visits) will be pulled from claims data at 90 days.

Prior admissions: The number of inpatient admissions in 90 days prior to index admission will be pulled at baseline from claims data.

ICU stay, mechanical ventilation, inpatient dialysis: Whether participant received each type of care during index hospitalization and if so, for how many days will be pulled at baseline from the electronic health record and claims data.

Skilled nursing facility stay: Whether a patient had a post-index hospitalization stay in a SNF for 28 days or fewer before being discharged home.

Index hospital: The location of the index hospitalization, hospital bed size, and hospital teaching status will be pulled from claims data at baseline.

A summary of measures is shown below:

STUDY VARIABLES	DATA SOURCE	WHEN MEASURED	HOW MEASURED
**Primary Outcome**			
Post-discharge home days	Claims data, EHR data, Health Information Exchange (HIE) data, Participant	90 days	Total number of days alive and out of acute care hospitals, inpatient rehabilitation, or skilled nursing facilities[80,81]
**Secondary Outcomes**			
Functional status	Participant	Baseline, 90 days	PROMIS Physical Function – for Mobility Aid Users[77,78]
Health-related Quality of Life	Participant	Baseline, 90 days	Quality of Life Enjoyment and Satisfaction Questionnaire-SF (QLESQ-SF)[79]
Transition to Hospice	Claims data	90 days	Whether member transitioned to hospice care
Emergent outpatient utilization	Claims data, EHR data, HIE data, Participant	90 days	Count of urgent outpatient visits (ED visits & urgent care visits)
Readmissions	Claims data, EHR data, HIE data, Participant	7, 30, and 90 days	Subsequent inpatient admission after index admission, and whether readmission hospital was same as index hospital[82]
Mortality	Claims data, EHR data	90 days	Whether member dies during observation period
**Covariates**			
Domain: Patient-Level			
Patient demographics & clinical characteristics	Administrative & claims data	Baseline	Age, race, gender, insurance type, Charlson Comorbidity Index[20,83]
Living at home status	Participant	Baseline	Whether participant lives alone or with others
Payer	EHR data	Baseline	Whether participant is insured by UPMC Health Plan or Medicare Fee-for-Service
Domain: Socioecological			
Distance to primary care provider (PCP) office	Administrative data	Baseline	Distance from participant's home address to PCP office (miles)
Area Deprivation Index (ADI)	Administrative data	Baseline	Composite score of socioeconomic variables to indicate neighborhood SES[84]
Domain: Care Delivery & Utilization			
Non-urgent outpatient care utilization	Claims data, EHR data, Participant	90 days	Count of non-urgent outpatient visits that occur during the observation period (primary and specialty care visits)
Prior admissions	Claims data, EHR data	Baseline	Number of inpatient admissions in 90 days prior to index admission
ICU stay, mechanical ventilation, inpatient dialysis	EHR/Claims data	Baseline	Whether participant received each type of care during index hospitalization and if so, for how many days
Skilled nursing facility stay	EHR/clinicians	Baseline	Whether the patient had a post-index hospitalization stay in a SNF for 28 days or fewer before being

			discharged home
Index hospital	EHR/Claims data	Baseline	Location of index hospitalization, hospital bed size, and teaching status
**Process**			
Integrity of care/fidelity	Research staff, clinicians	90 days	Checklist for RM and care approaches/processes
RPM compliance	Research staff, clinicians	90 days	RPM response rates

Process measures

The study team will monitor adherence to pre-established intervention workflows and RPM response rates throughout the study to ensure that intervention fidelity is maintained and can be accounted for in the analyses.

Integrity of care/fidelity: The research staff in conjunction with the remote monitoring supervisors following the RPM usual workflow, have developed an Interrater Reliability checklist to monitor fidelity to RPM intervention protocols and processes. This check will be conducted by either supervisors or peers every quarter to ensure established processes are being followed.

Fidelity to the STS arm protocol will be ensured by the telephonic case manager supervisor's routine monitoring of post-discharge phone calls. The research team will receive quarterly reports documenting that each study participant randomized to the STS arm received the STS usual care activities in accordance with usual care.

Participant RPM compliance: The research staff will review RPM response rate data at 90 days to characterize patient compliance to the RPM interventions.

> *No Response:* Participants who are enrolled in the RPM program, but do not begin the RPM program on their electronic device will be contacted by the RPM call center staff to address any technology issues or questions that have arisen during the first seven days of enrollment. If the patient still has not interacted with the RPM program after 14 days, the Research Team will attempt one final call to the participant to address issues. If the participant still does not participate after the call from the Research Team, the participant will remain able to join the program at any point during the 90-day post-discharge intervention period, but will no longer be contacted and encouraged to participate in the program by the research team or the RPM staff.

> *Non-Responder:* Patients who start the RPM program but fail to respond to RPM questions for 2 consecutive weeks are considered "non-responders". The remote monitoring call center staff will review non-responders' charts for hospital readmission, death, or admission to hospice. If the patient has not been readmitted, died, or went to hospice, they will be contacted by RPM staff to offer assistance and encouragement to respond. If there is still no participation after RPM staff outreach, the participant will continue to receive automated invitations to respond to pathways, but will no longer be encouraged to participate by the research team or RPM staff.

Study Duration/ Timeline

August 1, 2020 – December 31, 2020: Pre-Implementation Period
January 1, 2021 – October 31, 2024: Implementation Period
November 1, 2024 – July 31, 2026: Post-Implementation Period

Participants will be consented and enrolled in the hospital. Baseline surveys will be collected during participants' the first two weeks at home. The study observation period will be for 90 days post discharge to home (whether from hospital or SNF). Ninety-day surveys will be collected one week prior to and two weeks after the end of the observation period. This timeline is documented in the diagram below.

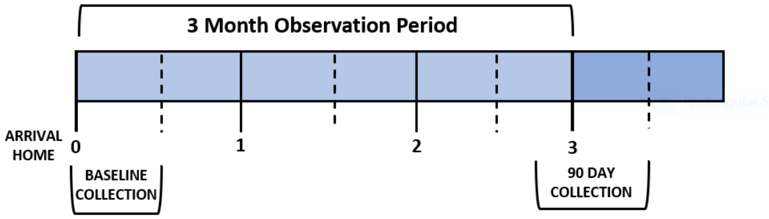

Data Collection

Quantitative data is collected from several sources. Self-report assessments are gathered at baseline and 90-days using REDCap Cloud, a secure data collection platform. Clinical data, additional patient demographic/descriptive information, and health care utilization data is extracted from electronic health records and payer claims at baseline and 90-days. For individuals who withdraw from the study, data will be retained up to the point of study withdrawal.

Qualitative telephone interviews are conducted to understand patient or caregiver intervention-related experiences. We will conduct interviews with 80 patients (spread approximately evenly across each of the 4 intervention arms). If a patient is unable to participate in an interview due to medical complexity, we attempt to interview his/her primary caregiver. All audio recordings of interviews are transcribed by experienced qualitative researchers and de-identified. We use a unique study ID to label all study materials related to participation with a tracking log as the only link to identifying information. Transcripts will be stored on a secure cloud-based environment approved by UPMC.

We use the NVivo 12 (QSR International, Australia) platform for data analysis. A constant comparative thematic content approach will be used to analyze the qualitative data. Our qualitative analysis is be iterative. A separate codebook is be developed for each interview type (i.e. patient, intervention providers, primary care physicians) with the input of the Co-Investigators and the Stakeholder Advisory Board. We assess inter-rater reliability using percent agreement and kappa scores. Upon acceptable interrater reliability, the qualitative research team thematically codes all qualitative documents.

Sample Size

The study planned to enroll 1,668 patients across the five different arms (STS, RPM-Low + Standard Team, RPM-Low + Enhanced Team, RPM-High + Standard Team, and RPM-High + Enhanced Team). From this sample 1168 patients were expected to be discharged directly to home and 500 patients were expected to be discharged to SNF and then home. Accounting for an estimated 10% attrition, the final analytic sample size was expected to be 1,500.
However, in November 2023 it was decided to reduce the sample size to 1,281 due to recruitment challenges.

Informed Consent Process

The informed consent process begins with a member of the hospital staff. The staff will ask pre-identified, potentially eligible patients if they are interested in learning more about a research study. If the patient is interested and agrees to learn more about the study, the hospital staff member will inform the Research Team members and outreach for consent will begin.

Electronic, verbal, or paper-based consent for the study will be obtained during a hospital admission at various UPMC and non-UPMC sites. Research Team members, who have completed institution-specific Human Research Protection Office (HRPO) CITI trainings, will assist the subject in navigating the consent process. The consent process will provide subjects with a clear explanation of the objectives, procedures, risks and benefits of the study and their rights as study participants. A Frequently Asked Questions (FAQ) page will also be available to the subjects. Members of the Research Team begin the consent process by reading through an IRB approved script explaining the study and conduct a cognitive screener. If the patient is interested and meets eligibility criteria, the Research Team can continue with obtaining consent. Written or verbal consent with a proxy may also be obtained through an IRB approved proxy consent process if the participant has an altered mental state (fails any of the screener) but still wishes to participant in the study.

Research Team members will be available in-person, over the phone, or via video to answer all questions related to the informed consent process and study procedures prior to the member consenting to participation. The FAQ sheet will be available to members to serve as a quick reference guide to the study. Consent for the qualitative interviews will also be obtained during this process.

Participants recruited from non-UPMC hospitals will be asked to sign a hospital-specific medical records release form after signing the informed consent form. In the unlikely event that a participant recruited from a non-UPMC hospital is randomized to a remote patient monitoring intervention arm and there is insufficient hospitalization clinical information available in the UPMC Health Plan’s member portal, the signed release form will be provided to the remote patient monitoring call center so they can request the records needed to provide post-discharge remote patient monitoring care to the participant.

Participants will retain their right to have their questions answered by Principal or Co-Investigators or a member of the Research Team via telephone or through any other preferred method of communication. The Research Team believes that informed consent is an ongoing process in any study and so will continue to educate participants about the nature of the research and will address any questions that arise throughout the course of the study. These efforts will comply fully with the Health Insurance Portability and Accountability Act (HIPAA) and the informed consent guidelines of the University of Pittsburgh IRB.

Participants who have a SNF stay prior to going home will be reminded of their study enrollment by a UPMC care coordinator (if available) while preparing to go home.

Privacy and Confidentiality

Self-report and qualitative data collected for this study will be used for research purposes only. Access to the data will be restricted to the Co-Principal Investigators, Co-Investigators, and other Research Team members. Research staff will sign confidentiality agreements as required. Identifying links to all data will be maintained at baseline and other data collection time points using a secure password-protected server, accessible only by authorized members of the study team. Once the data is collected, the file containing the link between identifying information and the participant's data will be destroyed at the earliest timepoint possible.

Qualitative data will be audio recorded using digital recording devices, transferred to a secure server immediately following recording and deleted as soon as it is fully transcribed. Interviews will be audio recorded, transcribed, and scrubbed of identifying information. Participants will be asked not to include any identifying information in their responses, and coders will not code any identifying information that may be provided inadvertently.

All data obtained over the course of the study will be confidential and secure. Paper study records will be secured using the “double-lock” method (i.e., in a locked cabinet within a locked office). Data stored on computers will be password protected and stored on a secure server behind each organization’s firewall. Each Research Team member has a unique network account and a secure password that complies with existing institutional policies and procedures. Only members of the team who are authorized by the site investigator leads will have access to secured files. Identities of participants will not be revealed in the publication or presentation of any results from this study.

Risks and Benefits to Participants

There is no known serious health or psychological risk of participating in this study. Some of the items in the self-report measures that ask respondents about their health, attitudes, and experiences could be considered sensitive. However, participants can refuse to answer questions at any time, which will be made clear during the consenting procedures and at the time of administration of all research measures and interviews. Minimal risks of study participation may still apply, such as participants feeling inconvenienced by the assessment requirements and/or uncomfortable or frustrated when responding to surveys or interviews. As with all research involving personal health information (PHI), there is also a potential risk of a breach of confidentiality for study participants. However, safeguards will be in place to decrease this risk to the fullest extent possible. Currently, there are no known risks associated with involvement in the study interventions, other than possible emotional discomfort associated with the discussion of issues related to complex health conditions. In instances where participants appear to be unduly distressed, they will be advised to speak directly with one of the study co-PIs.

All adverse events (e.g., mild distress following survey administration, breach of confidentiality) will be reported to the study co-PIs within 48 hours. The study team will ensure that each event is handled in the best interest of participants. If a serious adverse event (SAE) occurs, study team members will meet to discuss the event and its relevance to study participation. All SAEs will also be reviewed by the Data Safety and Monitoring Board (DSMB) as part of regularly scheduled meetings and procedures will be refined to prevent future events. Adverse events will be compiled and reported to the IRB as required.

There is a high likelihood that all study participants will benefit from the evidence-based components of care delivered through study interventions. Moreover, greater knowledge about the comparative effectiveness of the interventions and their impact on patient-centered outcomes will eventually benefit all patients hospitalized with sepsis, pneumonia or lower respiratory tract infection. For these reasons, we believe that the potential benefits of the knowledge to be gained from the proposed study outweigh the minimal risks posed to participants.

Compensation for Participation

Total possible compensation for the entire study: $65

- Time point 1: $20 for baseline self-report measures
- Time point 2: $20 for 3-month self-report measures
- Qualitative participant interview: $25

Study Oversight

Dr. Jatin Dave (Co-PI) and Dr. Sachin Yende (Co-PI) will be responsible for data and safety monitoring. Both will regularly meet with the entire study team to review all data protocols and policies, including informed consent and data confidentiality procedures. All key personnel will adhere to the National Institutes of Health (NIH) policy on education in the protection of human subject participants in the conduct of research. Additionally, we will convene biannual Data Safety and Monitoring Board (DSMB) meetings to ensure any issues related to participant or data safety are fully addressed. Interim data reports will be shared with the DSMB so they can provide feedback related to interim analyses, data quality and missingness. During these meetings, all unanticipated problems and adverse events will be discussed to obtain DSMB input.

The study protocol will be submitted to the University of Pittsburgh IRB. In addition, formal modifications will be submitted for any protocol changes to the University of Pittsburgh IRB for review and approval.

The Co-PIs and Stakeholder Co-I Dr. Rana Awdish will co-facilitate the stakeholder advisory board (SAB) meetings. The board is comprised of sepsis patients/caregivers, patient advocacy organizations, critical care providers, payers, and researchers/providers specializing critical care medicine. The board meets twice yearly to monitor study progress, provide feedback on study and intervention implementation and contributes to translation of study findings.

Conflict of Interest

The study investigators report having no conflicts of interest or financial interests related to the research conducted under this PCORI contract.

Dissemination

We will leverage the unique expertise of our Stakeholder Co-Investigator, SAB, and other partnering entities to support appropriate dissemination. Further, Erin Kelly, Senior Advisor, UPMC Health Plan, is supported by grant funds to produce relevant short-form content (e.g., blog posts, infographics, checklists, case studies) to share study results through appropriate audience-specific UPMC marketing channels. A comprehensive toolkit of strategies for optimal remote patient monitoring (RPM) and care team implementation will be developed to support dissemination. It will include lessons learned; implementation tools and resources; patient population-specific information; and strategies to

implement each intervention while considering variations such as delivery system/size, staffing requirements, payer support, and clinical workflows.

We will develop and maintain a plan that addresses data sharing of our research project's de-identified data with other researchers for future use in a manner that is appropriate for the nature of the research project data, and that is consistent with applicable privacy, confidentiality, and other legal requirements.

References

1. Torio C, Moore B. National inpatient hospital costs: the most expensive conditions by payer, 2013: statistical brief# 204. 2006;

2. Gijsen R, Hoeymans N, Schellevis FG, Ruwaard D, Satariano WA, van den Bos GA. Causes and consequences of comorbidity: a review. *Journal of clinical epidemiology*. 2001;54(7):661-674.

3. Jencks SF, Williams MV, Coleman EA. Rehospitalizations among patients in the Medicare fee-for-service program. *New England Journal of Medicine*. 2009;360(14):1418-1428.

4. Center for Health Information and Analysis. Performance of the Massachusetts Health Care System Series: A Focus on Provider Quality. http://www.chiamass.gov/assets/Uploads/A-Focus-on-Provider-Quality-Jan-2015.pdf

5. Mayr FB, Talisa VB, Balakumar V, Chang C-CH, Fine M, Yende S. Proportion and cost of unplanned 30-day readmissions after sepsis compared with other medical conditions. *Jama*. 2017;317(5):530-531.

6. Thomas CP, Ryan M, Chapman JD, et al. Incidence and Cost of Pneumonia in Medicare Beneficiaries. *Chest*. 2012/10/01/ 2012;142(4):973-981. doi:https://doi.org/10.1378/chest.11-1160

7. Bohannon RW, Maljanian RD. Hospital readmissions of elderly patients hospitalized with pneumonia. *Connecticut medicine*. 2003;67(10):599-603.

8. Dharmarajan K, Hsieh AF, Lin Z, et al. Diagnoses and Timing of 30-Day Readmissions After Hospitalization for Heart Failure, Acute Myocardial Infarction, or PneumoniaHeart Failure, Acute MI, or Pneumonia Readmissions. *JAMA*. 2013;309(4):355-363. doi:10.1001/jama.2012.216476

9. Raut M, Schein J, Mody S, Grant R, Benson C, Olson W. Estimating the economic impact of a half-day reduction in length of hospital stay among patients with community-acquired pneumonia in the US. *Current medical research and opinion*. 2009;25(9):2151-2157.

10. Mangen M-JJ, Huijts SM, Bonten MJM, de Wit GA. The impact of community-acquired pneumonia on the health-related quality-of-life in elderly. journal article. *BMC Infectious Diseases*. March 14 2017;17(1):208. doi:10.1186/s12879-017-2302-3

11. Holter JC, Ueland T, Jenum PA, et al. Risk factors for long-term mortality after hospitalization for community-acquired pneumonia: A 5-year prospective follow-up study. *PloS one*. 2016;11(2):e0148741.

12. Eurich DT, Marrie TJ, Minhas-Sandhu JK, Majumdar SR. Ten-year mortality after community-acquired pneumonia. A prospective cohort. *American journal of respiratory and critical care medicine*. 2015;192(5):597-604.

13. Iwashyna TJ, Ely EW, Smith DM, Langa KM. Long-term cognitive impairment and functional disability among survivors of severe sepsis. *Jama*. 2010;304(16):1787-1794.

14. Yende S, Angus DC. Long-term outcomes from sepsis. *Current infectious disease reports*. 2007;9(5):382-386.

15. El Solh A, Pineda L, Bouquin P, Mankowski C. Determinants of short and long term functional recovery after hospitalization for community-acquired pneumonia in the elderly: role of inflammatory markers. journal article. *BMC Geriatrics*. August 09 2006;6(1):12. doi:10.1186/1471-2318-6-12

16. Chang DW, Tseng C-H, Shapiro MF. Rehospitalizations following sepsis: common and costly. *Critical care medicine*. 2015;43(10):2085.

17. Prescott HC, Langa KM, Iwashyna TJ. Readmission diagnoses after hospitalization for severe sepsis and other acute medical conditions. *Jama*. 2015;313(10):1055-1057.

18. Axon RN, Williams MV. Hospital Readmission as an Accountability Measure. *JAMA*. 2011;305(5):504-505. doi:10.1001/jama.2011.72

19. Novosad SA. Vital signs: epidemiology of sepsis: prevalence of health care factors and opportunities for prevention. *MMWR Morbidity and mortality weekly report*. 2016;65

20. Gadre SK, Shah M, Mireles-Cabodevila E, Patel B, Duggal A. Epidemiology and Predictors of 30-Day Readmission in Patients With Sepsis. *Chest*. Mar 2019;155(3):483-490. doi:10.1016/j.chest.2018.12.008
21. Prout AJ, Talisa VB, Carcillo JA, et al. Children with Chronic Disease Bear the Highest Burden of Pediatric Sepsis. *The Journal of pediatrics*. Aug 2018;199:194-199.e1. doi:10.1016/j.jpeds.2018.03.056
22. DeVita MA, Smith GB, Adam SK, et al. "Identifying the hospitalised patient in crisis"—a consensus conference on the afferent limb of rapid response systems. *Resuscitation*. 2010;81(4):375-382.
23. Howell MD, Ngo L, Folcarelli P, et al. Sustained effectiveness of a primary-team-based rapid response system. *Critical care medicine*. 2012;40(9):2562-2568. doi:10.1097/CCM.0b013e318259007b
24. Leppin AL, Gionfriddo MR, Kessler M, et al. Preventing 30-day hospital readmissions: a systematic review and meta-analysis of randomized trials. *JAMA internal medicine*. 2014;174(7):1095-1107.
25. Gorodeski EZ. Home-based care for heart failure. *OPTIMIZING HOME HEALTH CARE: ENHANCED VALUE AND IMPROVED OUTCOMES*. 2013;54:20.
26. Koff P, Jones RH, Cashman JM, Voelkel NF, Vandivier R. Proactive integrated care improves quality of life in patients with COPD. *European Respiratory Journal*. 2009;33(5):1031-1038.
27. Bylander J. Community-Focused Health Care For The Seriously Ill. 2019.
28. Burke RE, Coleman EA. Interventions to decrease hospital readmissions: keys for cost-effectiveness. *JAMA internal medicine*. 2013;173(8):695-698.
29. Feltner C, Jones CD, Cené CW, et al. Transitional care interventions to prevent readmissions for persons with heart failure: a systematic review and meta-analysis. *Annals of internal medicine*. 2014;160(11):774-784.
30. Coye MJ, Haselkorn A, DeMello S. Remote patient management: technology-enabled innovation and evolving business models for chronic disease care. *Health Affairs*. 2009;28(1):126-135.
31. Langsetmo L, Platt RW, Ernst P, Bourbeau J. Underreporting exacerbation of chronic obstructive pulmonary disease in a longitudinal cohort. *American journal of respiratory and critical care medicine*. 2008;177(4):396-401.
32. Inglis SC, Clark RA, Dierckx R, Prieto-Merino D, Cleland JG. Structured telephone support or non-invasive telemonitoring for patients with heart failure. *Cochrane Database of Systematic Reviews*. 2015;2015(10)
33. Wong FKY, Chow SKY, Chan TMF, Tam SKF. Comparison of effects between home visits with telephone calls and telephone calls only for transitional discharge support: a randomised controlled trial. *Age and ageing*. 2013;43(1):91-97.
34. Ritchie CS, Houston TK, Richman JS, et al. The E-Coach technology-assisted care transition system: a pragmatic randomized trial. *Translational behavioral medicine*. 2016;6(3):428-437.
35. De San Miguel K, Smith J, Lewin G. Telehealth remote monitoring for community-dwelling older adults with chronic obstructive pulmonary disease. *Telemedicine and e-Health*. 2013;19(9):652-657.
36. Kitsiou S, Paré G, Jaana M. Effects of home telemonitoring interventions on patients with chronic heart failure: an overview of systematic reviews. *Journal of medical Internet research*. 2015;17(3)
37. Ranganathan A, Dougherty M, Waite D, Casarett D. Can palliative home care reduce 30-day readmissions? Results of a propensity score matched cohort study. *Journal of palliative medicine*. Oct 2013;16(10):1290-3. doi:10.1089/jpm.2013.0213
38. Brian Cassel J, Kerr KM, McClish DK, et al. Effect of a Home-Based Palliative Care Program on Healthcare Use and Costs. *Journal of the American Geriatrics Society*. Nov 2016;64(11):2288-2295. doi:10.1111/jgs.14354

39. Durie C, Tanksley-Bowe C. Rural Readmissions in the Palliative Care Vacuum. *Journal of hospice and palliative nursing : JHPN : the official journal of the Hospice and Palliative Nurses Association*. Apr 2018;20(2):160-165. doi:10.1097/njh.0000000000000421

40. Nouryan CN, Morahan S, Pecinka K, et al. Home telemonitoring of community-dwelling heart failure patients after home care discharge. *Telemedicine and e-Health*. 2018;

41. Piña IL. Transitions of Care in Heart Failure: The Case for Comparative Effectiveness Research. *Current heart failure reports*. 2011;8(2):81-83.

42. Killbourne AM, Prenovost KM, Liebrecht C, al. e. Randomized Controlled Trial of a Collaborative Care Intervention for Mood Disorders by a National Commercial Health Plan. *Psychiatric Services*. 2019;70(3):219-224. doi:10.1176/appi.ps.201800336

43. American Telemedicine Association. 2019 State of the States Report: Coverage and Reimbursement. Accessed August 20, 2019, https://www.americantelemed.org/initiatives/2019-state-of-the-states-report-coverage-and-reimbursement/

44. McLaughlin G. Why telemedicine and remote patient monitoring demand will skyrocket in 2019. Accessed August 20, 2019, https://www.redoxengine.com/blog/telemedicine-remote-patient-monitoring-demand-2019/

45. Wicklund E. CMS to Reimburse Providers for Remote Patient Monitoring Services. Accessed August 20, 2019, https://mhealthintelligence.com/news/cms-to-reimburse-providers-for-remote-patient-monitoring-services

46. American Telemedicine Association. American Telemedicine Association Advocacy Initiatives Result in New Medicare Coverage for Chronic Condition Monitoring. Accessed August 20, 2019, https://www.americantelemed.org/press-releases/american-telemedicine-association-advocacy-initiatives-result-in-new-medicare-coverage-for-chronic-condition-monitoring/

47. Brumley RD, Enguidanos S, Cherin DA. Effectiveness of a home-based palliative care program for end-of-life. *Journal of palliative medicine*. Oct 2003;6(5):715-24. doi:10.1089/109662103322515220

48. Wicklund E. Partners Launches Patient-Facing mHealth App to Gather RPM Data. Accessed August 20, 2019, https://mhealthintelligence.com/news/partners-launches-patient-facing-mhealth-app-to-gather-rpm-data

49. Moriarty A. The State of the Remote Patient Monitoring Market in 2019. Accessed August 20, 2019, https://blog.definitivehc.com/remote-patient-monitoring-market-2019

50. Pennic F. 2 in 3 Physicians Plan to Use Remote Patient Monitoring in the Future. Accessed August 20, 2019, https://hitconsultant.net/2019/04/15/cta-remote-patient-monitoring-survey/#.XVwVRehKiUl

51. American Heart Association. Using Remote Patient Monitoring Technologies for Better Cardiovascular Disease Outcomes Guidance. Accessed August 20, 2019, https://www.heart.org/-/media/files/about-us/policy-research/policy-positions/clinical-care/remote-patient-monitoring-guidance-2019.pdf?la=en&hash=A98793D5A043AB9940424B8FB91D2E8D5A5B6BEB

52. Yu J, Mink PJ, Huckfeldt PJ, Gildemeister S, Abraham JM. Population-Level Estimates Of Telemedicine Service Provision Using An All-Payer Claims Database. *Health affairs (Project Hope)*. Dec 2018;37(12):1931-1939. doi:10.1377/hlthaff.2018.05116

53. HealthCare Partners. Coordinated Care Model. Accessed August 20, 2019, https://healthcarepartners.com/PhysicianResources/caremodule.aspx?mid=63

54. Morrison R, Meier D. *America's Care of Serious Illness*. 2015.

55. Yao NA, Rose K, LeBaron V, Camacho F, Boling P. Increasing Role of Nurse Practitioners in House Call Programs. *Journal of the American Geriatrics Society*. Apr 2017;65(4):847-852. doi:10.1111/jgs.14698

56. The California State University Institute for Palliative Care. Advanced Practice RN Certificate in Palliative Care. Accessed August 20, 2019, https://csupalliativecare.org/programs/aprn/

57. University of Illinois at Chicago. Advanced Practice Palliative Care Nursing Certificate. Accessed August 20, 2019, https://nursing.uic.edu/programs/certificate-programs/advanced-practice-palliative-care/
58. Vital Talk. Courses. Accessed August 20, 2019, https://www.vitaltalk.org/courses/
59. Defining Hope. Accessed August 20, 2019, https://hope.film/study-guide-videos-full-version/
60. Lazur B, Bennett A, King V. The Evolving Policy Landscape of Telehealth Services Delivered in the Home and Other Nonclinical Settings. https://www.milbank.org/publications/the-evolving-policy-landscape-of-telehealth-services-delivered-in-the-home-and-other-nonclinical-settings/?utm_source=Milbank+Email+List&utm_campaign=fe0c5b9b3f-EMAIL_CAMPAIGN_2017_12_06_COPY_01&utm_medium=email&utm_term=0_dbce9df54c-fe0c5b9b3f-3865333
61. Mattimore TJ, Wenger NS, Desbiens NA, et al. Surrogate and Physician Understanding of Patients' Preferences for Living Permanently in a Nursing Home. *Journal of the American Geriatrics Society*. 1997;45(7):818-824. doi:10.1111/j.1532-5415.1997.tb01508.x
62. Ryan AA, McCann S, McKenna H. Impact of community care in enabling older people with complex needs to remain at home. *International Journal of Older People Nursing*. 2009;4(1):22-32.
63. Stepler R. *Smaller Share of Women Ages 65 and Older are Living Alone*. 2016.
64. Berry SM, Connor JT, Lewis RJ. The platform trial: an efficient strategy for evaluating multiple treatments. *Jama*. 2015;313(16):1619-1620.
65. Meurer WJ, Lewis RJ, Berry DA. Adaptive Clinical Trials: A Partial Remedy for the Therapeutic Misconception?Adaptive Clinical Trials. *JAMA*. 2012;307(22):2377-2378. doi:10.1001/jama.2012.4174
66. Barker A, Sigman C, Kelloff G, Hylton N, Berry D, Esserman L. I-SPY 2: an adaptive breast cancer trial design in the setting of neoadjuvant chemotherapy. *Clinical Pharmacology & Therapeutics*. 2009;86(1):97-100.
67. Alexander BM, Ba S, Berger MS, et al. Adaptive global innovative learning environment for glioblastoma: GBM AGILE. *Clinical Cancer Research*. 2018;24(4):737-743.
68. Berry DA. Adaptive clinical trials in oncology. *Nature reviews Clinical oncology*. 2012;9(4):199.
69. Bateman RJ, Benzinger TL, Berry S, et al. The DIAN-TU Next Generation Alzheimer's prevention trial: adaptive design and disease progression model. *Alzheimer's & Dementia*. 2017;13(1):8-19.
70. Schultz A, Marsh JA, Saville BR, et al. Trial Refresh: A Case for an Adaptive Platform Trial for Pulmonary Exacerbations of Cystic Fibrosis. *Frontiers in Pharmacology*. 2019;10
71. Khoury MJ, Galea S. Will precision medicine improve population health? *Jama*. 2016;316(13):1357-1358.
72. Health UDo, Services H. Multiple chronic conditions—a strategic framework: optimum health and quality of life for individuals with multiple chronic conditions. *Washington, DC: US Department of Health and Human Services*. 2010;2
73. Brunton L, Bower P, Sanders C. The Contradictions of Telehealth User Experience in Chronic Obstructive Pulmonary Disease (COPD): A Qualitative Meta-Synthesis. *PLoS One*. 2015;10(10):e0139561. doi:10.1371/journal.pone.0139561
74. Inglis SC, Conway A, Cleland JG, Clark RA. Is age a factor in the success or failure of remote monitoring in heart failure? Telemonitoring and structured telephone support in elderly heart failure patients. *European Journal of Cardiovascular Nursing*. 2015;14(3):248-255.
75. Graham J, Tomcavage J, Salek D, Sciandra J, Davis DE, Stewart WF. Postdischarge monitoring using interactive voice response system reduces 30-day readmission rates in a case-managed Medicare population. *Medical care*. 2012:50-57.
76. Gomes B, Calanzani N, Curiale V, McCrone P, Higginson IJ. Effectiveness and cost-effectiveness of home palliative care services for adults with advanced illness and their caregivers. *Cochrane Database of Systematic Reviews*. 2013;(6)

77. Cella D, Riley W, Stone A, et al. The Patient-Reported Outcomes Measurement Information System (PROMIS) developed and tested its first wave of adult self-reported health outcome item banks: 2005–2008. *Journal of clinical epidemiology*. 2010;63(11):1179-1194.
78. Cook KF, Jensen SE, Schalet BD, et al. PROMIS measures of pain, fatigue, negative affect, physical function, and social function demonstrated clinical validity across a range of chronic conditions. *J Clin Epidemiol*. May 2016;73:89-102. doi:10.1016/j.jclinepi.2015.08.038
79. Endicott J, Nee J, Harrison W, Blumenthal R. Quality of Life Enjoyment and Satisfaction Questionnaire: a new measure. *Psychopharmacology bulletin*. 1993;29(2):321-6.
80. Fonarow GC, Liang L, Thomas L, et al. Assessment of Home-Time After Acute Ischemic Stroke in Medicare Beneficiaries. *Stroke*. Mar 2016;47(3):836-42. doi:10.1161/strokeaha.115.011599
81. Greene SJ, O'Brien EC, Mentz RJ, et al. Home-Time After Discharge Among Patients Hospitalized With Heart Failure. *Journal of the American College of Cardiology*. Jun 12 2018;71(23):2643-2652. doi:10.1016/j.jacc.2018.03.517
82. Kothari AN, Loy VM, Brownlee SA, et al. Adverse Effect of Post-Discharge Care Fragmentation on Outcomes after Readmissions after Liver Transplantation. *Journal of the American College of Surgeons*. Jul 2017;225(1):62-67. doi:10.1016/j.jamcollsurg.2017.03.017
83. Charlson M, Szatrowski TP, Peterson J, Gold J. Validation of a combined comorbidity index. *Journal of clinical epidemiology*. 1994;47(11):1245-1251.
84. Singh GK. Area deprivation and widening inequalities in US mortality, 1969–1998. *American journal of public health*. 2003;93(7):1137-1143.

Domain-Specific Appendix:
ACCOMPLISH DOMAIN

Adaptive trial to test comparative effectiveness of readmission reduction approaches following infection and sepsis hospitalizations (ACCOMPLISH)

ACCOMPLISH Domain-Specific Appendix Version 1, January 18, 2021

Updated August 5, 2021

Updated March 3, 2022

Updated November 22, 2022

Updated November, 2023

Updated June 3, 2024

Updated April 9,2025

Summary

Study Population: Adults hospitalized for sepsis, lower respiratory tract or COVID-19 infection, age 21 and older with medium or high-risk of readmission.

Design: A comparative effectiveness study using individual-level response adaptive randomization design along with a pragmatic, mixed-methods approach to compare combinations of five strategies (e.g., structured telephone support, low intensity remote patient monitoring (RPM) questions, high intensity RPM questions, standard call center response team, enhanced call center response team). Quantitative (e.g., self-report, electronic health record, process) and qualitative (e.g., interviews) data will be collected across multiple time points during the study period.

Sample Size: Anticipated enrollment: 1,668 individuals.

**REMAP Post-Acute Care: ACCOMPLISH Domain Summary**	
Interventions	• Structured Telephone Support<br>• High Intensity Remote Patient Monitoring<br>• Low Intensity Remote Patient Monitoring<br>• Standard Team<br>• Enhanced Team
Prognostic Strata	Analyses and response adaptive randomization are adjusted for prognostic stratification variables (illness severity and discharge to skilled nursing facility [1])
Predictive Strata	None
Evaluable Interactions	ACCOMPLISH is designed as a 2x2 factorial design testing 2 levels of RPM intensity crossed with 2 types of nurse response team.
Timing of Reveal	Randomization with delayed reveal of allocation
Domain-Specific Inclusions	Inclusion criteria include:<br>• UPMC Health Plan member or patient with Medicare Fee-for-Service insurance<br>• Treated at one of 18 prespecified hospitals for sepsis or lower respiratory tract infection, including COVID-19<br>• Age 21 or older<br>• Medium or high-risk of readmission
Domain-Specific Exclusions	Patients will be excluded from this domain if they have any of the following:<br>• Admitted from setting other than home or independent living<br>• Receiving hospice at time of admission<br>• Being discharged on/to hospice or long-term acute care (LTAC)<br>• Currently participating in UPMC Advanced Illness Care program<br>• Unable to participate in RPM due to lack of technology/internet connection<br>• Known pregnancy<br>• Severe, persistent cognitive impairment<br>• Patients with no documented PCP<br>• Patients whose PCP disapproves of the patient potentially being enrolled in remote patient monitoring<br>• Patients who are discharged from hospital to SNF and stay at the SNF for greater than 28 days
Intervention-Specific Exclusions	There are no intervention-specific exclusions.
Outcome measures	Primary endpoint: Post-Discharge Home Days at 90 days<br>Secondary Domain-specific endpoints:<br>• Functional status<br>• Health-related quality of life<br>• Transition to hospice<br>• Emergent outpatient utilization<br>• Readmissions<br>• Mortality

TABLE OF CONTENTS

1. ABBREVIATIONS 7

2. DEFINITIONS 8

3. BACKGROUND AND RATIONALE 9

4. ENROLLMENT CRITERIA 11

4.1. Population 11

4.2. Eligibility criteria 11

4.2.1. Inclusion criteria for this domain 11

4.2.2. Exclusion criteria from this domain 11

5. INTERVENTIONS 12

5.1. Timing of initiation of interventions 13

5.2. Duration of remote interventions 13

5.3. Concomitant care 13

6. ENDPOINTS 14

6.1. Primary endpoint 14

6.2. Secondary endpoints 14

7. TRIAL CONDUCT 15

7.1. Data collection 15

7.2. Blinding 16

8. STATISTICAL ANALYSIS PLAN 16

8.1. Statistical Objectives 16

8.2. Intervention delivery 17

8.2.1. Intervention data 17

8.2.2. Intervention fidelity 18

8.3. Bayesian adaptive design elements 18

8.3.1. Statistical modeling 18

8.3.2. Bayesian ordinal logistic regression 18

8.3.3. Model priors 19

8.3.4. Model Stability 21

8.4. Posterior summaries 21

8.4.1. Evaluation of posterior estimates ... 21
8.4.2. Probability of arm superiority over other intervention arms ... 21
8.4.3. Probability of arm superiority over STS ... 22
8.4.4. Probability of harm ... 22
8.5. Proportional odds assumption ... 22
8.5.1. Assessing the proportional odds assumption ... 22
8.5.2. Extending the proportional odds model ... 22
8.6. Timing of interim analyses ... 23
8.7. Randomization ... 23
8.8. Futility stopping ... 24
8.9. Analytic approach ... 24
8.9.1. Final analysis of primary endpoint ... 24
8.9.2. Additional analysis of primary endpoint ... 25
8.9.3. Final analysis of mortality and PDHD among survivors ... 25
8.9.4. Final analysis of inpatient hospital readmissions ... 25
8.9.5. Final analysis of ordinal outcomes truncated by death ... 26
8.9.6. Final analysis of hospice care ... 27
8.9.7. Sensitivity analyses exploring the potential impact of payer type ... 27
8.9.8. Sensitivity analysis limited to UPMC Health Plan members with coverage for the duration of follow up 28
8.10. Analyses of heterogeneity of treatment effect ... 28
8.10.1. Comparison group determination for heterogeneity of effect analysis ... 28
8.10.2. Analysis of effect heterogeneity across pre-determined subgroups ... 28
8.10.3. Analysis of effect heterogeneity on dichotomized outcomes ... 29
8.11. Handling of missing data ... 30
8.11.1. Missing PDHD data ... 30
8.11.2. Missing data for secondary endpoints and baseline covariates ... 30
8.12. Type 1 error rate ... 31
9. ETHICAL CONSIDERATIONS ... 31
9.1. Data Safety and Monitoring Board ... 31
9.2. Potential domain-specific adverse events ... 31
9.3. Domain-specific consent issues ... 32
10. FUNDING ... 32

10.1. Funding of domain 32
10.2. Funding of domain interventions 32
10.3. Domain-specific declarations of interest 33
11. REFERENCES 34
12. Appendix 38
12.1. Sepsis data for simulations 38
12.2. Virtual patient simulation 39
12.3. Simulated treatment effect scenarios 40
12.4. Accrual 41
12.5. Operating characteristics when assumptions are met 41
12.6 Operating characteristics when assumptions are violated 48

1. ABBREVIATIONS

ACCOMPLISH	Adaptive trial to test comparative effectiveness of readmission reduction approaches following infection and sepsis hospitalizations
ADI	Area Deprivation Index
AE	Adverse Event
CI	Confidence Interval
CER	Comparative Effectiveness Research
CRNP	Certified Registered Nurse Practitioner
DSA	Domain-Specific Appendix
DSMB	Data Safety and Monitoring Board
DSWG	Domain-Specific Working Group
ED	Emergency Department
HQL	Health-related Quality of Life
ICU	Intensive Care Unit
ITT	Intent-To-Treat
LRTI	Lower respiratory tract infection
MC FFS	Medicare Fee-for-Service
MCMC	Markov Chain Monte Carlo
OR	Odds Ratio
PCP	Primary care practitioner
PDHD	Post-Discharge Home Days
RPM	Remote Patient Monitoring
RAR	Response Adaptive Randomization
RCT	Randomized Controlled Trial
REMAP	Randomized, Embedded, Multifactorial Adaptive Platform trial
RPM	Remote Patient Monitoring
SAE	Serious Adverse Event
SNF	Skilled Nursing Facility
STS	Structured Telephone Support
UPMC	University of Pittsburgh Medical Center

2. DEFINITIONS

Care team: The individual or group of medical professionals whose role it is to screen RPM alerts and follow up with the patient, referring to the patient's PCP or other specialists if necessary.

Home: Level of residence or health care facility where the patient was residing prior to hospital admission.

Heterogeneity of Treatment Effects (HTE): The hypothetical scenario in which the effect of intervention on outcomes depends on baseline covariates.

Index Hospitalization: Hospitalization during which patient was enrolled in the clinical trial.

Intention To Treat (ITT): All eligible and consented patients who undergo randomization will be included in the ITT cohort for the purposes of analyzing the primary and secondary study outcomes.

Intervention arms: RPM-Low + Standard team, RPM-Low + Enhanced team, RPM-High + Standard team, RPM-High + Enhanced team.

Remote Patient Monitoring (RPM): An automatic electronic messaging system designed to push questions to patients at fixed intervals. Questions vary depending on the intensity of the RPM program (Low or High), but patient answers can trigger alerts and responses by members of the intervention care team.

Study day: The day of discharge to home is study day 0. The next calendar day is study day 1, etc.

Study hospital: Defined as the hospital where the patient was randomized and enrolled.

Study withdrawal: Defined as permanent withdrawal from study before completion of study activities. If a patient or surrogate requests withdrawal from the study, the clinician should seek explicit permission to continue data collection.

Prognostic strata: patient groups with different PDHD distributions.

Predictive strata: patient groups in which intervention effects are estimated individually.

3. BACKGROUND AND RATIONALE

Readmissions after sepsis or pneumonia exact an enormous toll on the health and quality of life of individuals and pose significant challenges for the health care system. Approximately, one in five Medicare beneficiaries discharged from the hospital are readmitted within 30 days and a third in 90 days,[2] and older adults with multimorbidity are at higher risk.[3] Returning to the hospital after an index admission is shown to worsen patients' quality of life and increase their risk of healthcare-acquired infections,[4] despite the fact that many of these readmissions are avoidable.[5]

Individuals hospitalized for sepsis or pneumonia are particularly at risk for readmission and poor health outcomes. Among 14 million readmissions in the National Readmissions Database, sepsis and pneumonia (~3 million cases annually in US) were leading causes of readmissions.[6] Readmissions following sepsis and pneumonia are more common and associated with higher costs than readmissions due to heart failure, chronic obstructive pulmonary disease (COPD), or myocardial infarction.[2,6-10] Survivors of both conditions experience reduced quality of life, increased mortality and morbidity,[11-13] and cognitive impairment and functional limitations.[14-16] Despite readmissions, one in three patients do not survive the year following sepsis or pneumonia, and those who do survive often experience persistent health deficits.[7-9,11,17] In a subset of hospitals owned by UPMC, a large integrated delivery and finance system in Pennsylvania, all-cause 30-day readmission rates for individuals with sepsis or pneumonia (23%) exceed the overall average (12%).

Further, readmissions following sepsis or pneumonia are often preventable. Up to half of readmissions after sepsis can be attributed to preventable causes[18] and the preventability of readmission after pneumonia is recognized by the Centers for Medicare and Medicaid Services' (CMS) Hospital Readmission Reduction Program as an indicator of quality of care.[19] Most patients hospitalized with sepsis or pneumonia are >65 years of age and have multimorbidity.[20,21] Two-thirds of readmissions following sepsis or pneumonia were due to infection (~40%) and cardiovascular (15%) and respiratory disease (10%).[22] In theory, adequately monitoring patients, detecting early signs of deterioration, and triaging them to appropriate care would decrease unnecessary care utilization and increase patient stability. This is the premise of several widespread interventions, such as in-hospital rapid response teams, post-discharge remote patient monitoring (RPM) for heart failure, and primary care-led monitoring, in the community.[23,24]

However, there are critical gaps in knowledge regarding how comprehensively we should remotely monitor patient symptoms and types of health care teams best suited for responding to alerts to maximize patient time at home. The standard of care for patients with multimorbidity is to provide some level of care coordination or care provision after discharge, as we know it works. There is a large body of evidence demonstrating that post-acute care models as a whole reduce the risk of an individual returning to the hospital after discharge.[25] Among these, more complex interventions that leverage multiple components and multiple care providers have greater effect on maintaining patients at home longer after discharge.[25] Specific successful model components include care coordination/care management activities and patient self-management education.[26,27]

A key priority for the health care system is understanding how to effectively deploy the post-acute workforce in the community.[28,29] Structured Telephone Support (STS), or telephonically connecting with patients post-discharge to assess health status, reconcile medications, and provide patient education, is a well-studied and effective strategy. Within UPMC, deployment of STS for several years across a wide population has successfully reduced readmissions. However, STS may be insufficient for patients at higher risk of readmission.[30] For more complex patients, RPM platforms have extended the reach of providers by regularly collecting patient-reported data on vital signs and symptoms, allowing early recognition of worsening disease and deploying interventions promptly.[31] Provider monitoring of symptom exacerbation is important because patients alone are unreliable in reporting worsening symptoms.[26,32] RPM platforms have received FDA approval, reduced readmissions in multiple observational and randomized controlled studies, and are routinely used in clinical care.[26,33] While patient acceptability of RPM is high, RPM platforms typically monitor one disease or condition (e.g., heart failure exacerbation in patients hospitalized for heart failure). Given that multimorbidity is associated with hospitalization for serious infection and readmissions are often due to worsening of underlying chronic conditions, utilizing RPM to assess multiple disease states could be more successful in early identification and intervention. However, the optimal post-acute care strategy for patients with multimorbidity is unknown.

Additionally, more information about the spectrum of services delivered within and alongside RPM is critical to effectively meeting patients' needs. There is conflicting evidence about whether models that couple RPM with an alert-triggered provider response (Standard Team) or a more intensive patient engagement model (Enhanced Team) are more effective,[34-36] the reason for which may be that studies often fail to define specific intervention components.[37] Several studies show that multidisciplinary teams that periodically conduct in-person or virtual home visits and specialize in chronic disease management, care coordination, and patient education about self-management may increase time spent at home.[38,39] However, this approach is resource intense and may be difficult to scale across large geographic areas.[40] Noting that RPM findings are often confounded by additional in-home care received by both intervention and control groups, one study measured the effect of RPM versus usual outpatient care in a sample of patients not receiving concurrent home-based care and showed decreased emergency department (ED) utilization and hospital length of stay and increased quality of life among the group receiving RPM.[41] The study of RPM without explicit investigation of the model of care into which RPM is built is a critical gap in the comparative effectiveness literature.[42]

4. ENROLLMENT CRITERIA

4.1. *Population*

This domain enrolls adults hospitalized for sepsis or lower respiratory tract infection, including COVID-19, age 21 and older, medium or high-risk of readmission, and whose primary outpatient physician is willing to participate in RPM.

4.2. *Eligibility criteria*

Patients are eligible for this domain if they meet all of the REMAP-level inclusion and none of the REMAP-level exclusion criteria (see Core Protocol Section 6.4). Patients who may be eligible for the REMAP may have conditions that may exclude them from the ACCOMPLISH Domain.

4.2.1. Inclusion criteria for this domain

Patients will be included in this domain if they meet the following criteria:

- UPMC Health Plan member or patient with Medicare Fee-for-Service insurance
- Treated at one of 19 prespecified hospitals for sepsis or lower respiratory tract infection
- Age 21 or older
- Medium or high-risk of readmission

4.2.2. Exclusion criteria from this domain

Patients will be excluded from this domain if they have any of the following:

- Admitted from setting other than home or independent living
- Receiving hospice at the time of admission
- Being discharged on/to hospice or LTAC
- Currently participating in UPMC Advanced Illness Care Program
- Unable to participate in RPM due to lack of technology/internet connection
- Known pregnancy
- Severe, persistent cognitive impairment
- No documented primary care physician (PCP)
- PCP disapproves of the patient potentially being enrolled in remote patient monitoring

5. INTERVENTIONS

ACCOMPLISH tests the impact of a set of active arms compared with standardized telephone support (STS):

- Structured **Telephone Support (STS):**[30] Structured telephone support (STS) consists of post-discharge assessment, education, and medication reconciliation delivered telephonically by a case manager, home care as needed, and follow-up appointment with the primary care within seven days post-discharge.

The following types of interventions are used across the active arms:

- **Low-intensity Remote Patient Monitoring (RPM-Low):**[43] Questions are pushed to members three times per week for up to 90-days post-discharge. Questions are limited to those checking vital signs that indicate worsening of infection. Patient answers RPM questions, which trigger high or medium alerts and a response by members of the intervention care team (see below).
- **High-intensity** Remote **Patient Monitoring (RPM-High):**[23] Questions are pushed to members three times per week for up to 90-days post-discharge. Questions include monitoring vital signs for worsening infection but also ask about factors that would indicate worsening of underlying heart or lung conditions, such as weight gain or shortness of breath. Patient answers to RPM questions, which trigger high or medium alerts and a response by members of the intervention care team (see below).
- **Standard Team**: [41,44] RPM alerts are screened by a nurse-staffed call center. Nurses determine whether immediate outreach to the member is warranted and facilitate connection to the appropriate care (such as referring the member to their PCP, specialist, or instructing the patient to go the emergency department).
- **Enhanced Team**: [27,38,45] RPM alerts are screened by a nurse-staffed call center and alert a certified registered nurse practitioner (CRNP) to modify care plans. CRNPs, who operate in a palliative care role, have prescribing authority and will also facilitate connection to the appropriate care. In addition to reacting to RPM triggers, CRNPs and other members of an interdisciplinary palliative care team (e.g., social workers, nurses) meet with the patient in-person or virtually at least three times in 90 days, conduct assessments, develop care plans, and complete a physician order for life-sustaining treatment.

Patients are randomized to either STS, or active arm; if randomized to active arm, they are assigned to 1 level of RPM (RPM-Low or RPM-High) and 1 type of care team (standard or enhanced care team), leading to 4 active intervention arms:

- RPM-Low + Standard Team
- RPM-Low + Enhanced Team
- RPM-High + Standard Team
- RPM-High + Enhanced Team.

A graphical representation of the interventions is shown in Figure 1.

Figure 1: Study Design

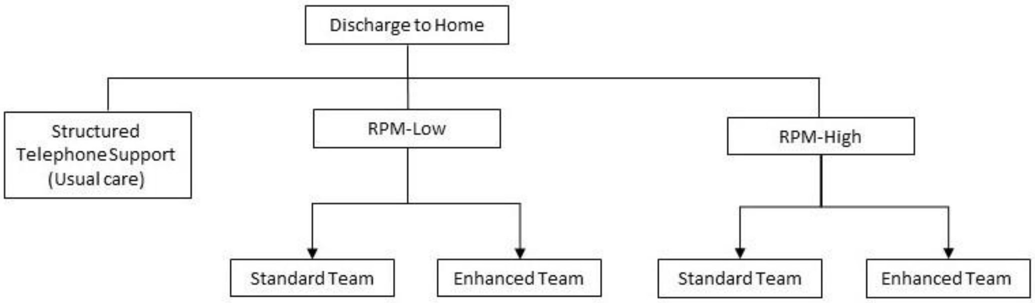

5.1. *Timing of initiation of interventions*

Participants will be enrolled in the appropriate intervention (as randomized) by their first full week day home from the hospital or skilled nursing facility (SNF). Participants enrolled in a RPM arm will be contacted by a research coordinator by telephone and encouraged to download the RPM application and initiate the program. If enrolled in structured telephone support, they will receive a post-discharge phone call per usual care timing.

5.2. *Duration of remote interventions*

The duration of standard telephone support will align with usual care, but is expected to be limited to one brief telephone conversation, in most cases. The duration of remote patient monitoring interventions is 90 days.

5.3. *Concomitant care*

The intent of this trial is to observe participants as they participate in real-world post-discharge health care. Therefore, there are very few concomitant care restrictions. Remote patient monitoring pathways will be paused during hospital readmissions and/or admissions to other inpatient care settings. The RPM program will be stopped if the study team learns that a patient entered hospice or expired. Most of other health care utilization will be observed and analyzed during analysis.

The UPMC Advanced Illness Care (AIC) Program is a 6 month palliative care program offered by UPMC Health Plan to members in need of palliative care. Participants who are known to be currently participating in the Advanced Illness Care program will be excluded from study recruitment. Referrals to this program are made both at hospital discharge and during the course of routine outpatient care. It is rare but possible for a randomized ACCOMPLISH participant to be enrolled into the UPMC Advanced Illness Care (AIC) Program during the three month study intervention period. In the event that a randomized ACCOMPLISH participant is also enrolled in the AIC program, the study team will follow a strict intent to treat study design and continue to collect outcomes data per the established timelines and processes. If the number of randomized participants is greater than 10 at the end of all recruitment and data collection, a sensitivity

analysis will be performed to determine if the AIC participants have significantly different outcomes than the participants enrolled in the study as planned.

6. ENDPOINTS

6.1. *Primary endpoint*

The primary efficacy endpoint will be post-discharge home-days (PDHD) recorded over the first 90 days after discharge to the patient's home. Patients will be assigned -1 days if they die before day 90 post-discharge home. Among patients who survive 90 days or more, PDHD is recorded as the total number of days alive and out of acute care hospitals or post-acute care facilities (e.g., inpatient rehabilitation, skilled nursing facilities, long-term care facility). If a patient is readmitted to one of these facilities during the 90 days after returning home, then those days not at home will be subtracted from their PDHD. The following rules will be used to calculate PDHD.

- Home is defined as any location that does not involve insurance claims charged to UPMC Health Plan. This can include the patient's own residence, someone else's residence (family or friend), independent living facility or personal care home. It is not restricted to being the same location at which the patient resided pre-admission, so long as it does not involve UPMC Health Plan claims.
- The index hospitalization and post-acute health care facilities (e.g., long-term acute care facility, ventilator-rehabilitation unit, rehabilitation facility, transitional care units, skilled nursing care, nursing homes) visited following discharge are counted as "hospitals" until the patient is discharged home.
- PDHD can take on integer values from -1 to 90. Any calendar day partially spent in acute care hospitals or post-acute care facilities is counted as a full day for the purposes of calculating PDHD, regardless of whether it was part of the initial admission or any readmission.

6.2. *Secondary endpoints*

The domain-specific secondary outcome measures are:

<u>*Functional Status:*</u> will be measured at baseline and 90 days, using the PROMIS Physical Function – for Mobility Aid Users-SF. The Patient-Reported Outcomes Measurement Information System (PROMIS™) provides item banks that offer the potential for PRO measurement that is efficient (minimizes item number without compromising reliability) flexible (enables optional use of interchangeable items), and precise (has minimal error in estimate) measurement of

commonly-studied PROs.[46] PROMIS item banks and their short forms provide evidence they are reliable and precise measures of generic symptoms and functional reports comparable to legacy instruments.[47]

Health-related Quality of Life: will be measured at baseline and 90 days, using the Quality of Life Enjoyment and Satisfaction Questionnaire-SF. The Quality of Life Enjoyment and Satisfaction Questionnaire (Q-LES-Q) is a self-report measure designed to enable investigators to easily obtain sensitive measures of the degree of enjoyment and satisfaction experienced by subjects in various areas of daily functioning.[48]

Transition to Hospice: will be assessed via claims data at 90 days to see if members transitioned to hospice care during the course of the study.

Emergent outpatient utilization: will be assessed via claims data at 90 days for a count of urgent outpatient visits for study participants.

Readmissions: will be assessed via claims data at day 7, 30, and 90 for any inpatient admissions after index admission, and whether readmission hospital was the same as index hospital.

Mortality: will be assessed via claims data at 90 days to see if and when the member/participant passes away.

7. TRIAL CONDUCT

7.1. *Data collection*

Quantitative data will be collected from several sources. Self-report assessments will be gathered at baseline and 90 days post discharge using REDCap Cloud, a secure data collection platform. Clinical data, additional patient demographic/descriptive information, and health care utilization data will be extracted from electronic health records and payer claims at baseline and 90-days. These data collection procedures have been used previously by our site teams (e.g., successfully retaining over 80% of participants in other large-scale studies). For individuals who withdraw from the study, data will be retained up to the point of study withdrawal.

Qualitative telephone interviews will be conducted to understand patient or caregiver intervention-related experiences. We will conduct the interviews as soon as possible following the 90-day follow-up to maximize participants recall related to involvement in the study. We will conduct interviews with 120 patients (30 patients from each of the 4 intervention arms). If a patient is unable to participate in an interview due to medical complexity, we will attempt to interview his/her primary caregiver. All audio recordings of interviews will be transcribed by experienced qualitative researchers and de-identified. We will use a unique study ID to label all study materials related to participation with a tracking log as

the only link to identifying information. Transcripts will be stored on a secure cloud-based environment approved by UPMC.

We will use the NVivo 12 (QSR International, Australia) platform for data analysis. A constant comparative thematic content approach will be used to analyze the qualitative data. Our qualitative analysis will be iterative and will take place over the course of the qualitative data collection period. For example, we will review transcripts from the first few patient interviews to begin developing a codebook and to make needed modifications to our interview guide for subsequent interviews. A separate codebook will be developed for each interview type (i.e., patient, intervention providers, primary care physicians) with the input of the Co-Investigators and the Stakeholder Advisory Board. We will assess inter-rater reliability using percent agreement and kappa scores. Upon acceptable interrater reliability, the qualitative research team will thematically code all qualitative documents.

7.2. *Blinding*

All study staff will be blinded to the adaptive intervention arm randomization probabilities. Study Co-Principal Investigators, Co-Investigators, and employed (non-consultant) statisticians will be blinded to the participant enrollment totals per named intervention arm.   Teams caring for the patients (e.g., primary care physicians) will be unblinded to the interventions. All study staff will be completely unblinded after data collection is complete.

8. STATISTICAL ANALYSIS PLAN

8.1. *Statistical Objectives*

The following objectives and endpoints are relevant to the final statistical analysis of the ACCOMPLISH trial.

Primary analyses

- For Aim #1, the primary analyses will be to compare PDHD between the 5 study arms (STS and the 4 experimental intervention arms):
    - **Aim 1a**: Efficacy will be evaluated by comparing PDHD between the STS arm and each of the 4 experimental arms, adjusting for illness severity (mechanical ventilation + vasopressors) and discharge location (whether patient went to SNF prior to home) and accounting for multiple comparisons
    - **Aim 1b**: Superiority of each experimental arm over the others will be evaluated by calculating the posterior probability that PDHD for each arm is greater than the others, adjusting for illness severity

(mechanical ventilation + vasopressors) and discharge location (whether patient went to SNF prior to home).

Secondary analyses

- To compare each intervention arm to STS, and to each other, with respect to mortality and PDHD among survivors.
- To determine the effects of each intervention arm and STS on secondary endpoints:
  - Functional status as measured by the PROMIS Physical Function—for Mobility Aid Users[46]
  - Health-related Quality of Life (HQL) as measured by the Quality of Life Enjoyment and Satisfaction Questionnaire-SF (QLESQ-SF) [48]
  - Transition to Hospice. Defined as whether the patient transitioned to hospice care.
  - Emergent outpatient care utilization. Defined as the count of urgent outpatient visits (ED visits and urgent visits).
  - Readmission. Defined as subsequent admission after index admission.
  - Mortality. Whether a patient dies during the first 90 days post discharge to home
- To explore potential heterogeneity of treatment effect

8.2. *Intervention delivery*

8.2.1. Intervention data

Data relating to the completion of intervention components will be summarized for each intervention arm separately. Intervention completion requires the cooperation of up to 3 components: automatic (computer generated) notifications, follow-up by the remote monitoring team (possibly in response to the automatic notification), and patient completion of follow-ups initiated automatically or by the response team.

The following RPM data will be collected:

- Number of RPM questionnaires sent to patients
- Number of RPM questionnaires completed by patients
- Number of yellow and red alerts sent to the monitoring team
- Number of yellow and red alerts addressed by the monitoring team within the appropriate time period
- Number of attempted in-person or virtual visits by the CRNP to the patient (enhanced team arms only)
- Number of medication reviews (enhanced team arms only)
- Number of documented advance directive/POLST discussions conducted (enhanced team arms only)

8.2.2. Intervention fidelity

Patients completing a minimum of intervention components (as outlined in section 8.2.1 above) will be considered as having followed the protocol for their intervention arm.

8.3. *Bayesian adaptive design elements*

8.3.1. Statistical modeling

The primary analysis is a Bayesian ordinal logistic regression model that accounts for differences in the 90-day PDHD depending on two patient strata that were significantly associated with outcomes in this population in a retrospective analysis of UPMC data (see Appendix): 1) illness severity (defined as patient needing ventilation or vasopressors), and 2) whether the patient was discharged to a SNF after they leave the hospital and prior to their discharge home. Treatment effects are modeled as a factorial where independent variables for RPM level (low or high) and type of team (standard or enhanced) are modeled with an interaction. The model assumes proportional treatment effects across the levels of the ordinal home days endpoint.

8.3.2. Bayesian ordinal logistic regression

The primary analysis employs a Bayesian cumulative logistic regression assuming proportional odds effects. Let $Y_i \in \{-1, 0, 1, \ldots, 89, 90\}$ be the primary ordinal 90-day home days endpoint for patient i. Let γ_{ij} denote the probability of patient *i* observing a home days endpoint of j or lower. For $j = -1, \ldots, 89$, the primary analysis model is formulated as:

$$\text{logit}(\gamma_{i,j}) = \alpha_j - (X_{i1}(\theta_1 + \theta_2 X_{i2} + \theta_3 X_{i3} + \delta X_{i2} X_{i3}) + \beta_1 Z_{i1} + \beta_2 Z_{i2} + W_{i,COV}\,\boldsymbol{\kappa}) \quad (1)$$

where $\text{logit}(\gamma_{ij}) = \log\left(\frac{\gamma_{ij}}{1-\gamma_{ij}}\right)$ are the log odds of the probability that Y_i is less than j. The variables, X_{i1}, X_{i2}, X_{i3}, are indicators for treatment assignment, where $X_{i1} = 1$ when randomized to one of the 4 care model interventions (interventions 2-5 above) and 0 when randomized to the STS arm, X_{i2} = 1 when randomized to an intervention with RPM high and $X_{i2} = 0$ for RPM low, X_{i3} =1 when randomized to an intervention with enhanced team and $X_{i3} = 0$ for standard team. The model is formulated such that STS is considered the referent treatment and all parameters learned in the model are in relation to STS. The four care models are modeled as a factorial comprised of the level of RPM and type of team deployed.

The parameters $\theta_1, \theta_2, \theta_3$ are log odds ratios for active interventions and the parameter, δ, is an interaction effect between RPM high and enhanced team. We assume that the effects of each intervention are constant across strata and can be explained by a proportional log-odds ratio shift in the home days distribution. Each intervention's treatment effect is made up of components of the parameters, such that the treatment effect for RPM low/standard is equal to θ_1, the effect for RPM high/standard is equal to $\theta_1 + \theta_2$, the effect for RPM low/enhanced is equal to $\theta_1 + \theta_3$, and the effect for RPM high/enhanced is equal to $\theta_1 + \theta_2 + \theta_3 + \delta$. We report these treatment effects as odds ratios, where $OR_1 = \exp(\theta_1)$ is the odds ratio for RPM low/standard, $OR_2 = \exp(\theta_1 + \theta_2)$ is the odds ratio for RPM high/standard, $OR_3 = \exp(\theta_1 + \theta_3)$ is the odds ratio for RPM low/enhanced, and $OR_4 = \exp(\theta_1 + \theta_2 + \theta_3 + \delta)$ is the odds ratio for RPM high/enhanced. The model is parameterized such that an odds ratio greater than 1 indicates that the care model is beneficial compared to STS.

The variables, Z_{i1} and Z_{i2}, are indicators of disease strata for patient i, where $Z_{i1} = 1$ when a patient has severe illness (spent time on mechanical ventilation or vasopressors while hospitalized) and $Z_{i1} = 0$ otherwise, and $Z_{i2} = 1$ if patient i has been discharged to a SNF and $Z_{i2} = 0$ if the patients is discharged immediately to home. With the above model, we assume that the underlying distribution of home days is different within each stratum, and that those differences can be explained by a proportional log-odds ratio shift in the home days distribution through the parameters, β_1 and β_2.

Finally, p additional covariates, $W_{i,COV}$, are included in the model with parameters, $\boldsymbol{\kappa}$. W_{COV} contain indicator variables for time at randomization, measured in buckets of 3-month intervals. The first time bucket (furthest in past) will be used as the referent in the model.

8.3.3. Model priors

The prior distribution of α_j is specified on the probability scale:

$$\alpha_j = \text{logit}\left(\sum_{k=-1}^{j} \pi_k\right), j = -1, \ldots, 89$$

where

$$\pi_{-1}, \ldots, \pi_{90} \sim \text{Dirichlet}(\phi_{-1}, \ldots, \phi_{90});$$

with hyperparameters, ϕ_j, specified based on the observed rates of home days across all states in pre-trial data (see Appendix) and providing 1 patient's worth of information so that $\sum_{h=-1}^{90} \phi_h = 1$. The vector $\boldsymbol{\phi}$ consists of concentration parameters with a weight of 0.133 on -1 (death), 0.005 on 0-19 (split evenly), 0.017 on 20-39 (split evenly), 0.017 on 40-49 (split evenly), 0.026 on 50-59 (split evenly), 0.042 on 60-69 (split evenly), 0.077 on 70-79 (split evenly), 0.228 on 80-89 (split evenly), and 0.454 on 90.

Because of the large ordinal scale, it is likely that some levels of the ordinal endpoint will be quite sparse. By using a prior centered on UPMC patient data, we help to guard against model instability issues on levels without observations early in the trial when fewer patients have been enrolled. The prior is parameterized to be weakly informative, such that as more patients are enrolled, the patient data quickly overwhelms the prior in the posterior estimation.

For each stratum-specific log odds ratio, we place a normal prior distribution with mean 0 and standard deviation of 1:

$$\beta_l \sim N(0, 1), l = 1, 2.$$

Additionally, for the covariates' log odds ratios, $\boldsymbol{\kappa}$, we specify the same normal distributed prior with mean 0 and standard deviation 1:

$$\kappa_k \sim N(0, 1), k = 1, 2, \dots, p - 1.$$

This standard normal prior on the log odds ratio induces a relatively non-informative prior on the odds ratio that is lognormally distributed with a prior median of 1 (no effect). The prior assumes there is a 95% probability that the odds ratio effect is between 0.14 and 7.10.

We specify independent, normally distributed priors on the log-odds treatment effects:

$$\theta_t \sim N(0, 1), t = 1, 2, 3.$$

This prior on the log odds ratio induces a relatively non-informative prior on the odds ratio that is lognormally distributed with a prior median of 1 (no effect). The prior assumes there is a 95% probability that the odds ratio effect is between 0.14 and 7.10.

For the interaction term, δ, we use the following prior:

$$\delta \sim N(0, \tau^2),$$

with the following prior on the variance term of the interaction:

$$\tau^2 \sim IG(0.8, 0.0525).$$

This inverse-gamma prior for τ^2 was chosen to encourage dynamic borrowing when learning the interaction term, δ. This prior allows the data to determine the amount of information that is borrowed from the two individual interventions (RPM High and Enhanced team). If τ^2 is close to zero, then there is an additive effect for the combination arm with RPM high and Enhanced team; if τ^2 is positive, then we assume a synergistic effect between RPM high and the Enhanced team (effect of the combination is greater than the sum of the two interventions); if τ^2 is negative, this assumes an antagonistic interaction (effect of the combination is less than the sum of the two interventions). The prior shape 0.8 and prior scale 0.0525 were chosen such that the prior for τ^2 has a median of 0.1 with 90% probability of being less than 1.

8.3.4. Model Stability

To assure model stability in the primary analysis model, the following modeling conventions may be taken at the statistical analysis committee's discretion.

- If there is an outcome in the ordinal scale that did not occur in the data, then that outcome will be combined with a neighboring outcome (the worse outcome). This is done for model stability. For example, if the outcome 11 never occurred, then a combined outcome of 10 & 11 will be modeled for the analysis.
- If a time bucket category has fewer than 5 patients observed in the category, it will be combined with the adjacent bucket (more recent bucket).

8.4. *Posterior summaries*

The following statistical quantities are used in the design of the trial. The posterior distribution of all model parameters is calculated using MCMC. The algorithm allows the generation of M (ex. 100,000) draws from the joint posterior distribution for all model parameters.

8.4.1. Evaluation of posterior estimates

The Bayesian model of the primary endpoint described above is fitted to the data at each interim analysis. The posterior is calculated as:

$$p(\varphi|Y) \propto \prod_{i=1}^{n} p\,(Y_i|\varphi)p(\varphi),$$

where φ is the set of parameters for the primary endpoint model, $p(\varphi)$ is the prior for those parameters, Y_i is the primary endpoint value for each patient, and n is the number of patients. The posterior is evaluated using MCMC using statistical software Stan.

8.4.2. Probability of arm superiority over other intervention arms

For each of the four care model arms, we calculate the probability an arm is superior to other active arms using the posterior probability that each arm is optimal. This quantity is calculated from the M samples of the posterior distribution of the effect of each arm by reporting the proportion of posterior samples in which the odds ratio for arm t is the maximum observed effect across all non-STS treatment arms, t=1,…,4:

$$O(t) = \frac{1}{M}\sum_{m=1}^{M} I\big[OR_t < OR_j, \quad for\ all\ j\ \neq t\big], \quad t = 1, \ldots.,4.$$

8.4.3. Probability of arm superiority over STS

To determine if the trial is successful at the final analysis, we summarize the posterior probability that each care model arm is superior to STS. The probability of superiority is calculated as the proportion of the M samples of an intervention's OR treatment effect that are greater than 1:

$$O(t) = \frac{1}{M}\sum_{m=1}^{M} I[OR_t > 1], \qquad t = 1\ \ldots.,4.$$

8.4.4. Probability of harm

To determine if an arm is futile at an interim analysis, we summarize the posterior probability that each care model arm is harmful compared to STS. The probability of harm is calculated as the proportion of the M samples of an intervention's OR treatment effect that are less than 1:

$$O(t) = \frac{1}{M}\sum_{m=1}^{M} I[OR_t < 1\}, \qquad t = 1\ \ldots.,4.$$

8.5. *Proportional odds assumption*

The ordinal logistic regression model assumes that the effect of a treatment on the odds of higher PDHD is the same for all levels of the outcome. For example, this assumption entails that the effect of an intervention on the odds of surviving through the 90-day post discharge period increases by an amount proportional to the odds of having greater than 80 PDHD. In the event that the interventions have no effect on survival, but a beneficial effect on PDHD among survivors, the proportionality assumption will be violated.

8.5.1. Assessing the proportional odds assumption

Assessment of the proportional odds assumption will occur once all patients are enrolled, or the study is ended for futility of one or more active arms over STS. We will dichotomize the endpoint at each level of the PDHD outcome, summarizing the odds ratio for each dichotomization and plotting for visual inspection of equality. Meaningful shifts in odds ratios across the ordinal scale will be interpreted as evidence of non-proportionality.

8.5.2. Extending the proportional odds model

To assess the proportional odds assumption around the lower end of the distribution, we propose the following non-proportional model in Equation (2):

$$\text{logit}\left(\gamma_{i,j}\right) = \alpha_j - \left(X_{i1}\left(\theta_{1,k} + \theta_{2,k}X_{i2} + \theta_{3,k}X_{i3} + \delta_k X_{i2}X_{i3}\right) + \beta_1 Z_{i1} + \beta_2 Z_{i2} + W_{i,COV}\,\boldsymbol{\kappa}\right), \qquad k = -1, \geq 0.\#(2)$$

where we allow the treatment effect to vary for death ($k = -1$) and stay constant across the home-days distribution (0-90). The effects for strata are constant across the full scale, -1:90.

This proposed model results in the probability of death for patients on STS equal to:

$$\Pr(Y_i = -1) = logit^{-1}(\alpha_{-1} - \left(\beta_1 Z_{i1} + \beta_2 Z_{i2} + W_{i,COV}\,\boldsymbol{\kappa}\right))$$

And the probability of death for patients on a treated arm:

$$\Pr(Y_i = -1) = logit^{-1}\left(\alpha_{-1} - \left(X_{i1}\left(\theta_{1,-1} + \theta_{2,-1}X_{i2} + \theta_{3,-1}X_{i3} + \delta_{-1}X_{i2}X_{i3}\right) + \beta_1 Z_{i1} + \beta_2 Z_{i2} + W_{i,COV}\,\boldsymbol{\kappa}\right)\right).$$

Then the remainder of the 90-day home days scale can be described using the usual proportional odds shift, for $j = 1, \ldots, 89$:

$$\Pr(Y_i \leq j) = logit^{-1}\left(\alpha_j - \left(X_{i1}\left(\theta_{1,\geq 0} + \theta_{2,\geq 0}X_{i2} + \theta_{3,\geq 0}X_{i3} + \delta_{\geq 0}X_{i2}X_{i3}\right) + \beta_1 Z_{i1} + \beta_2 Z_{i2} + W_{i,COV}\,\boldsymbol{\kappa}\right)\right).$$

The priors will be the same as specified in Section 8.3.3 for each level of the log-odds treatment effects, θ_k.

The qualitative interpretation of this model will be similar to the primary analysis model above; however, this formulation will allow us to assess how these interventions impact mortality versus the time at home.

Analyses of mortality and PDHD among survivors from the model in equation (2) will be considered separate secondary analyses. See Section 8.9.3 below.

8.6. *Timing of interim analyses*

The first interim analysis will occur when 250 patients have been followed for 90 days and have observed their 90-day PDHD. Subsequent interims will occur every 3 months until the total sample size has been enrolled, using data collected from all patients for whom days from randomization has elapsed and their 90-day PDHD has been recorded.

At each interim, response adaptative randomization (RAR) allocation probabilities will be updated according to the details described in Section 8.7

8.7. *Randomization*

Initial randomization is set so that 30% of patients are randomized to STS and 70% of patients are randomized equally to the remaining 4 arms. After a burn-in of 250 patients, RAR will begin. The allocation to STS will be maintained at 30% throughout the course of the trial. The remaining 70% of patients will be allocated to the 4 active arms preferentially based on the probability that the intervention is superior to the other active arms as defined in Section 8.4.2.

8.8. *Futility stopping*

The interventions selected for this proposal have been used in clinical care for other conditions (e.g., heart failure) and not shown to harm patients. Furthermore, RAR ensures that a higher proportion of patients randomized to one of the four intervention arms are randomized to the better-performing arms based on the results of the interim analyses. Thus, the likelihood of harm in our trial is low.

We considered the rare scenario that an arm performs worse than usual care, for which RAR will not change the allocation ratios. We therefore included a rule where an arm would be discontinued if the posterior probability of harm (odds ratio for home days <1) is >90%:

$$\text{Futility rule: } \Pr(OR < 1) > 0.9.$$

Based on preliminary simulations, we expect enrolling ~1200 patients before harm in this scenario is detected. Our simulations also ensured that our rule would prevent an arm to be dropped prematurely and likely to show benefit at the end of the trial.

Futility checking will occur at all interim analyses except the first to allow sufficient patient accrual into each treatment arm, preventing some probability of false declaration of futility. Futility is assessed separately for each individual care model arm. Declaration of futility for a particular arm during an interim analysis will trigger an early stopping of randomization to that arm, pending review by the DSMB. If all active arms are dropped for futility, then the entire trial would conclude early. The trial would conclude at the interim at which the final active arm was dropped. Our futility rule was selected by evaluating the operating characteristics of several rules via simulation (See Appendix, Section 12.5).

8.9. *Analytic approach*

Analyses described in this section pertain to the statistical analysis conducted at the conclusion of the ACCOMPLISH trial, after all data have been collected.

8.9.1. Final analysis of primary endpoint

The primary analysis of PDHD will consist of the posterior probability of superiority of each active arm over STS following posterior calculation from the model in Section 8.9.1. Success will be declared at the final analysis when all patients have observed their primary endpoint if the posterior probability of any care model relative to STS is greater than 0.991. This threshold was determined through simulation to assure the type I error is controlled at a 2.5% level. Specifically, we simulated 10,000 trials under the null scenario and find the threshold that controls overall 2.5% type I error (probability of declaring success under the null) across the simulated trial scenarios (Appendix Table 4).

We will also calculate the posterior probability that each active arm is superior to the other active arms (Section 8.4.2).

8.9.2. Additional analysis of primary endpoint

We will also estimate the marginalized effects of RPM low and RPM high compared to STS, and alternatively Standard team and Enhanced team compared to STS. These effects will be estimated in models blinded to one or the other intervention modality. For instance, to estimate the effects of RPM low and RPM high compared to STS, we will use the following modification of model (1):

$$\text{logit}(\gamma_{i,j}) = \alpha_j - (X_{i1}(\theta_1 + \theta_2 X_{i2}) + \beta_1 Z_{i1} + \beta_2 Z_{i2} + W_{i,COV}\,\boldsymbol{\kappa}) \quad (3)$$

The variables, X_{i1}, X_{i2} are indicators for treatment assignment, where $X_{i1} = 1$ when randomized to one of the 4 care model interventions and 0 when randomized to the STS arm, X_{i2} = 1 when randomized to an intervention with RPM high and $X_{i2} = 0$ for RPM low. The parameters θ_1, θ_2 are log odds ratios for active interventions; the treatment effect for RPM low/standard is equal to θ_1, the effect for RPM high/standard is equal to $\theta_1 + \theta_2$. An equivalent model will be used to estimate the effects of Standard team and Enhanced team type compared to STS.

From these models, we will estimate the prosterior probability that the intervention odds ratio is greater than one, and the appropriate posterior probability of superiority comparing interventions in the same model.

8.9.3. Final analysis of mortality and PDHD among survivors

Analysis of mortality and PDHD among survivors will be conducted jointly via the model in Section 8.5.2. This model relaxes the proportional odds assumption, allowing for the effect of interventions on mortality to be independent of the effect on PDHD among survivors. From this model, we will estimate the following posterior probabilities:

- Posterior probability of superiority of each active arm over STS with respect to probability of survival to 90 days
- Posterior probability that each active arm is superior to the other active arms with respect to probability of survival to 90 days
- Posterior probability of superiority of each active arm over STS with respect to PDHD among survivors
- Posterior probability that each active arm is superior to the other active arms with respect to PDHD among survivors

8.9.4. Final analysis of inpatient hospital readmissions

Analyses of inpatient hospital readmission will be performed using a Bayesian proportional odds model similar to the one used in section 8.3.3, but with ordinal outcome taking values of -1 for non-survivors to 90 days, 0 for survivors who were readmitted within 90 days, and 2 for survivors who were not readmitted within 90 days. We will first assume that the odds ratios of interventions are proportional across all levels of this outcome. Due to the similarity between

readmission and PDHD outcomes, we will use all model priors from the model in 8.3.3 in the analysis of readmissions. From this model, we will estimate the following posterior probabilities:

- Posterior probability of superiority of each active arm over STS with respect to probability of readmission or death to 90 days
- Posterior probability that each active arm is superior to the other active arms with respect to probability of readmission or death to 90 days

When mortality rates are low, the treatment effects estimated above will approximately equal to the effect on readmission prior to death. However, when mortality rates are high this may not be true[49]. We will therefore also estimate the effect of readmission prior to death on survival to 90 days directly using the (frequentist) Fine and Gray subdistribution hazard model. Coefficients in the linear predictor of this model will mimic the form of the linear predictor in the model of 8.3.3 (minus the intercept terms), including terms for each intervention, the interaction, calendar time if necessary, and relevant risk predictors. From this model, we will estimate the following quantities:

- Difference in cumulative incidence functions for readmission at 90 days and their 95% confidence intervals, for each intervention compared to STS
- Difference in cumulative incidence functions for readmission at 90 days and their 95% confidence intervals, for each intervention compared to other active intervention arms.

8.9.5. Final analysis of ordinal outcomes truncated by death

PROMIS and HRQoL are continuous outcomes collected around day 90 after discharge conditional on survival. Similarly, number of emergency visits by day 90 depends on having 90 days of follow up to be comparable between different treatment arms. Thus, to avoid selection bias we will analyze of these 3 outcomes using a similar composite outcome approach used for the primary outcome, wherein death before day 90 will be treated as the worst outcome on an ordinal scale otherwise consisting of the natural values taken by these variables. For PROMIS and HRQoL, which take positive values, deaths will be assigned a value of -1. For number of emergency visits by day 90, which also takes positive values up to the largest number of visits observed in the sample (K visits), deaths will be assigned a value of K+1. We will use a Bayesian proportional odds model to analyze these outcomes, similar to the approach of the primary analysis in 8.3.3. In our models, each unique value taken by these outcomes will receive its own intercept, a way of modeling continuous outcomes that is competitive with linear regression[50] but which allows us to account for death. The linear predictor of our model will otherwise mimic that of the primary analysis in 8.3.3. From these models, we will estimate the following quantities:

- Posterior probability of superiority of each active arm over STS in terms of surviving with better functional outcomes (PROMIS and HRQoL), or fewer emergency visits within 90 days.

- Posterior probability of superiority of each active arm over all other active arms in terms of surviving with better functional outcomes (PROMIS or HRQoL), or fewer emergency visits within 90 days.

In the event that mortality rates are very small, or that the effect of interventions on mortality rates are very small, we will perform a survivors-only analysis of these endpoints, which is likely to be minimally biased under these conditions[49]. In these cases, we will analyze these endpoints among survivors to 90 days post-discharge. When modeling these endpoints, we will use Bayesian linear (for PROMIS and HRQoL) or generalized linear (number of emergency visits by 90 days) regression with uninformative priors. The linear predictors of these models will mimic that of the primary analysis in 8.3.3. From these models, we will estimate the following quantities:

- Posterior probability of superiority of each active arm over STS in terms better functional outcomes (PROMIS and HRQoL), or fewer emergency visits within 90 days, among survivors.
- Posterior probability of superiority of each active arm over all other active arms in terms of better functional outcomes (PROMIS or HRQoL), or fewer emergency visits within 90 days, among survivors.

8.9.6. Final analysis of hospice care

Analysis of admission to hospice care will be performed using Bayesian Logistic regression with uninformative priors. The endpoint will be defined as any admission to hospice by day 90 post-discharge; any hospice care will be considered a successful outcome, while death within 90 days or survival without hospice care will be considered as non-successful outcome. The linear predictors the model will mimic that of the primary analysis in 8.3.3. From this model, we will estimate the following quantities:

- Posterior probability of superiority of each active arm over STS in terms larger odds of admission to hospice within 90 days.
- Posterior probability of superiority of each active arm over all other active arms in terms larger odds of admission to hospice within 90 days.

8.9.7. Sensitivity analyses exploring the potential impact of payer type

On August 10, 2022 the trial expanded its inclusion criteria to include Medicare Fee-For-Service (MC FFS) patients. We will conduct two types of sensitivity analyses to detect any differences in PDHD distribution between UPMC Health Plan and MC FFS patients:

- We will fit two additional proportional logit models accounting for payer type:
    - One logit model including a term for payer which will capture proportional shifts in home days across payer groups; and
    - A second model that will include different intercept terms for the payer type groups. This model will capture non-proportional shifts in home days across both groups.

- We will explore potential differential effects of RPM care models across payer types. One model will estimate interaction terms between RPM arm and payer type; and another will fit separate proportional logit models to UPMC Health Plan and MC FFS participants. We will also explore limiting these analyses to data collected after August 10, 2022 when MC FFS patients were eligible.

8.9.8. Sensitivity analysis limited to UPMC Health Plan members with coverage for the duration of follow up

We will conduct an analysis limited to patients with UPMC Health Plan coverage throughout the duration of the 90-day follow up period. FFS patients, as well as UPMC Health Plan members who lose or drop their coverage under the Plan before the end of the 90-day follow up period, will be excluded from this analysis. Outcomes ascertainment could be more accurate among patients included in this analysis. This analysis will be conducted using the model in 8.3.3.

8.10. *Analyses of heterogeneity of treatment effect*

8.10.1. Comparison group determination for heterogeneity of effect analysis

Because it is possible for our RAR design to reduce enrollment to under-performing arms, we may be at a power disadvantage for identifying heterogeneity of treatment effect (HTE) of each active intervention compared with STS as a function of baseline covariates: age, sex, race, chronic comorbidity index, comfort with technology, health literacy level, Area Deprivation Index, discharge location, illness severity and admitting diagnosis. Therefore, we may pool arms prior to HTE analysis based on arms' average effects. For instance, if both RPM-Low and RPM-High are on average effective, we will pool these patients. However, we may choose not to pool patients by care team component (Standard Team or Enhanced Team) since resource utilization for these components is different and stakeholders have expressed a strong interest in identifying subgroups that benefit, especially among those who receive the Enhanced Team component. Thus, all analyses described below will be repeated for comparisons between each active (and potentially pooled) intervention and STS arms.

8.10.2. Analysis of effect heterogeneity across pre-determined subgroups

We will conduct differential treatment effects analyses, comparing differences between RPM groups and STS across pre-specified subgroups of patient-level clinical, social, and environemntal factors that could be potentially used to tailor service delivery. All analyses will be performed using a cumulative logit model for the primary PDHD outcome, including terms for treatment arm, subgroup, treamtent by subgroup interactions, stratification variables, and calendar time of randomization. Subgroup variables and definitions were determined as plausibly related to treatment effect heterogeneity. Each subgroup variable will be analyzed in two ways: 1) four RPM arms kept separate; and 2) comparing standard team and enhanced team arms. Analyses will not be adjusted for multiple comparisons. Evidence of differential treatment effects across subgroup levels will be summarized using the posterior probability that interaction

terms are greater than 0. In addition, treatment effects by subgroup level will be summarized using the posterior median cumulative odds ratios from each model, and corresponding 95% credible intervals.

Subgroup definitions are as follows:

- Patient Age
    - <65 vs age >=65
- Sex
    - Female vs male
- Race
    - White patients vs Black patients
- Chronic Condition index
    - CCI<5 vs CCI>=5
- Level of comfort with technology
    - Comfortable vs uncomfortable vs missing
- Health literacy level
    - Limited vs marginal vs adequate vs missing
- State-ranked Area Deprivation Index
    - Quartile 1 vs quartile 2 vs quartile 3 vs quartile 4 vs missing
- Location after discharge
    - Skilled Nursing Facility vs home
- Severity of illness
    - Severe (mechanical ventilation or vasopressor use during index hospitalization) vs not severe
- Discharge diagnosis
    - COVID vs Non-COVID Sepsis vs Other lower respiratory tract infection

8.10.3. Analysis of effect heterogeneity on dichotomized outcomes

We will explore potential heterogeneity of the effects of active arms compared to control groups after dichotomizing the PDHD outcome, collapsing all values of PDHD less than 90 into a single category. This will facilitate the estimation of conditional average treatment effects on the risk difference scale [52].

For our first set of analyses, we will dichotomize continuous or ordinal covariates, and analyze the conditional absolute risk differences (cARD) between the levels of the dichotomized covariates. For each covariate, we will test the null hypothesis that there is no difference in the cARDs using the Q test [53]. We will also calculate 95% CI for the cARDs and their differences.

For our second set of analyses, we will use an "effect-based" approach to estimate ARDs conditional on multiple covariates simultaneously[54]. In this approach, we will divide the data into two halves in approximately equal proportion. The first half will be used for cARD model derivation, as well as any missing data imputation models. We will compare the performance of several competing cARD models with different advantages and disadvantages, including causal forest, local linear random forest R-learners, and regularized logistic regression[55-57]. We will compare these models in the derivation set using the following cross-validated performance metrics: area under the target operating characteristic curve (AUTOC) and R-loss[58]. The model with the best performance will be validated in the patients that were not used for model derivation. Model validation will be performed by examining the calibration of the model, including the estimation of calibration intercept, slope, and their 95% Cis[59]. The presence of HTE identified by the model will be determined if the calibration slope p-value is <0.05. In addition, we will estimate ARDs and 95% CIs within quantiles of predicted cARDs generated from the derived model for each patient in the validation set. We will test the null hypothesis that the quantile ARDs are not monotonically increasing across the quantiles[59]. Finally, to examine whether the results are dependent on the particular random derivation/validation split that was used, we will repeat the analyses across many different random splits and aggregate the results, including inferences, with the result from the original random split[59]. If we are able to identify HTE using our selected best-performing cARD prediction model, we will determine the value of the predicted cARD that serves as the best classification rule for identifying the subgroup that benefits from each candidate intervention.[60] Finally, we will explore which baseline characteristics predict treatment effectiveness by plotting the associations between baseline characteristics against cARD predictions, and a fit-the-fit approach using binary decision trees where the predicted cARD is treated as the outcome variable.[61]

8.11. *Handling of missing data*

8.11.1. Missing PDHD data

The primary endpoint is ascertained using claims data for the majority of patients, and medical records review for the small subset of patients that either drop UPMC Health Plan coverage during the course of follow up, or are covered by the Medicare Fee-for-Service program. We will assume that no record of death or readmissions counted against the primary endpoint correctly correspond to the absence of these events for the patient, and therefore the primary outcome will be completely observed for all patinets.

8.11.2. Missing data for secondary endpoints and baseline covariates

For binary and normally-distributed secondary endpoints as well as baseline covariates with missing data, we will generate 10 completed datasets via a Multiple Imputation with Chained Equations (MICE) algorithm for the secondary and sensitivity analyses specified.[62] MICE draws missing data values from a sequence of fully-specified regression

models. We will use logistic regression models for modeling missing binary data, and normal linear regression otherwise. The linear predictor for each regression model will include linear terms for the primary outcome (PDHD), all secondary outcomes, and all baseline covariates, in addition to first-order interactions with each intervention arm.

Once generated, we will use the 10 completed datasets to fit the appropriate analysis models, performing final aggregated estimation and inference using Rubin's rules.[62]

For the primary analysis, we will impute any missing baseline covariates based on the following logic:

- If the patient is missing a value for the severe illness indicator, we will impute the value to 0 (not severe).
- If the patient is missing a value for discharge location, we will impute the value to 0 (home).

We expect minimal missing baseline covariates (<1%) so are choosing imputation rules that are designed around the most likely categories given a value is missing.

8.12. *Type 1 error rate*

Success is declared at the final analysis when all patients have observed their primary endpoint if the posterior probability of any care model relative to STS is greater than 0.991. This threshold was determined through simulation to assure the type I error is controlled at a 2.5% level (see Appendix). Specifically, we simulate 10,000 trials under the null scenario and find the threshold that controls overall 2.5% type I error (probability of declaring success under the null) across the simulated trials.

9. ETHICAL CONSIDERATIONS

9.1. *Data Safety and Monitoring Board*

The DSMB will provide oversight for the trial.

9.2. *Potential domain-specific adverse events*

This study is expected to pose minimal risk. The STS intervention is used for patients recovering from sepsis, LRTI, and other diseases. RPM interventions used in this domain are commonly used as routine care for other diseases. As a result, anticipated adverse events (AE) will be uncommon and include mild distress following survey administration and breach of confidentiality. Other AEs will be reported only where, in the opinion of the co-Principal Investigators, the event might reasonably have occurred as a consequence of a study intervention or study participation (see Core Protocol Section 12). Additionally, it is expected that a high proportion of critically ill patients who will be enrolled in this trial will experience mortality, substantial morbidity and readmissions. These endpoints are included in the primary and secondary endpoints. Stopping rules in the rare scenario that an intervention worsens these endpoints are included in

the Statistical Appendix and data regarding the primary endpoint stratified by intervention and select secondary endpoints will be provided to the DSMB.

9.3. *Domain-specific consent issues*

Electronic, verbal, or paper-based consent for the study will be obtained during a hospital admission at various UPMC and non-UPMC sites. Research Team members, who have completed institution-specific Human Research Protection Office (HRPO) CITI trainings, will assist the subject in navigating the consent process. The consent process will provide subjects with a clear explanation of the objectives, procedures, risks and benefits of the study and their rights as study participants. A Frequently Asked Questions (FAQ) page will also be available to the subjects. Members of the Research Team begin the consent process by reading through an IRB approved script explaining the study and conduct a comprehension and cognitive screener. If the subject is interested and meets eligibility criteria, the Research Team can continue with obtaining consent. Written or verbal consent with a proxy may also be obtained through an IRB approved proxy consent process if the participant has an altered mental state (fails any of the screeners) but still wishes to participant in the study.

Research Team members will be available in-person, over the phone, or via video to answer all questions related to the informed consent process and study procedures prior to the subject consenting to participation. Consent for the qualitative interviews will also be obtained during this process.

Participants will retain their right to have their questions answered by Principal or Co-Investigators or a member of the Research Team via telephone or through any other preferred method of communication. The Research Team believes that informed consent is an ongoing process in any study and so will continue to educate participants about the nature of the research and will address any questions that arise throughout the course of the study. These efforts will comply fully with the Health Insurance Portability and Accountability Act (HIPAA) and the informed consent guidelines of the University of Pittsburgh IRB.

10. FUNDING

10.1. *Funding of domain*

The ACCOMPLISH domain is funded by the Patient Centered Outcomes Research Institute, contract #IHS-2019C1-16055.

10.2. *Funding of domain interventions*

The STS and RPM interventions in the ACCOMPLISH domain are funded by UPMC.

10.3. *Domain-specific declarations of interest*

All investigators involved in REMAP Post-Acute Care ACCOMPLISH domain maintain a declaration of conflicting interests with the Patient-Centered Outcomes Research Institute.

11. REFERENCES

1. Greene SJ, O'Brien EC, Mentz RJ, et al. Home-Time After Discharge Among Patients Hospitalized With Heart Failure. *Journal of the American College of Cardiology*. Jun 12 2018;71(23):2643-2652. doi:10.1016/j.jacc.2018.03.517

2. Torio C, Moore B. National inpatient hospital costs: the most expensive conditions by payer, 2013: statistical brief# 204. 2006;

3. Gijsen R, Hoeymans N, Schellevis FG, Ruwaard D, Satariano WA, van den Bos GA. Causes and consequences of comorbidity: a review. *Journal of clinical epidemiology*. 2001;54(7):661-674.

4. Jencks SF, Williams MV, Coleman EA. Rehospitalizations among patients in the Medicare fee-for-service program. *New England Journal of Medicine*. 2009;360(14):1418-1428.

5. Center for Health Information and Analysis. Performance of the Massachusetts Health Care System Series: A Focus on Provider Quality. http://www.chiamass.gov/assets/Uploads/A-Focus-on-Provider-Quality-Jan-2015.pdf

6. Mayr FB, Talisa VB, Balakumar V, Chang C-CH, Fine M, Yende S. Proportion and cost of unplanned 30-day readmissions after sepsis compared with other medical conditions. *Jama*. 2017;317(5):530-531.

7. Thomas CP, Ryan M, Chapman JD, et al. Incidence and Cost of Pneumonia in Medicare Beneficiaries. *Chest*. 2012/10/01/ 2012;142(4):973-981. doi:https://doi.org/10.1378/chest.11-1160

8. Bohannon RW, Maljanian RD. Hospital readmissions of elderly patients hospitalized with pneumonia. *Connecticut medicine*. 2003;67(10):599-603.

9. Dharmarajan K, Hsieh AF, Lin Z, et al. Diagnoses and Timing of 30-Day Readmissions After Hospitalization for Heart Failure, Acute Myocardial Infarction, or PneumoniaHeart Failure, Acute MI, or Pneumonia Readmissions. *JAMA*. 2013;309(4):355-363. doi:10.1001/jama.2012.216476

10. Raut M, Schein J, Mody S, Grant R, Benson C, Olson W. Estimating the economic impact of a half-day reduction in length of hospital stay among patients with community-acquired pneumonia in the US. *Current medical research and opinion*. 2009;25(9):2151-2157.

11. Mangen M-JJ, Huijts SM, Bonten MJM, de Wit GA. The impact of community-acquired pneumonia on the health-related quality-of-life in elderly. journal article. *BMC Infectious Diseases*. March 14 2017;17(1):208. doi:10.1186/s12879-017-2302-3

12. Holter JC, Ueland T, Jenum PA, et al. Risk factors for long-term mortality after hospitalization for community-acquired pneumonia: A 5-year prospective follow-up study. *PloS one*. 2016;11(2):e0148741.

13. Eurich DT, Marrie TJ, Minhas-Sandhu JK, Majumdar SR. Ten-year mortality after community-acquired pneumonia. A prospective cohort. *American journal of respiratory and critical care medicine*. 2015;192(5):597-604.

14. Iwashyna TJ, Ely EW, Smith DM, Langa KM. Long-term cognitive impairment and functional disability among survivors of severe sepsis. *Jama*. 2010;304(16):1787-1794.

15. Yende S, Angus DC. Long-term outcomes from sepsis. *Current infectious disease reports*. 2007;9(5):382-386.

16. El Solh A, Pineda L, Bouquin P, Mankowski C. Determinants of short and long term functional recovery after hospitalization for community-acquired pneumonia in the elderly: role of inflammatory markers. journal article. *BMC Geriatrics*. August 09 2006;6(1):12. doi:10.1186/1471-2318-6-12

17. Chang DW, Tseng C-H, Shapiro MF. Rehospitalizations following sepsis: common and costly. *Critical care medicine*. 2015;43(10):2085.

18. Prescott HC, Langa KM, Iwashyna TJ. Readmission diagnoses after hospitalization for severe sepsis and other acute medical conditions. *Jama*. 2015;313(10):1055-1057.

19. Axon RN, Williams MV. Hospital Readmission as an Accountability Measure. *JAMA*. 2011;305(5):504-505. doi:10.1001/jama.2011.72

20. Novosad SA. Vital signs: epidemiology of sepsis: prevalence of health care factors and opportunities for prevention. *MMWR Morbidity and mortality weekly report*. 2016;65

21. Gadre SK, Shah M, Mireles-Cabodevila E, Patel B, Duggal A. Epidemiology and Predictors of 30-Day Readmission in Patients With Sepsis. *Chest*. Mar 2019;155(3):483-490. doi:10.1016/j.chest.2018.12.008

22. Prout AJ, Talisa VB, Carcillo JA, et al. Children with Chronic Disease Bear the Highest Burden of Pediatric Sepsis. *The Journal of pediatrics*. Aug 2018;199:194-199.e1. doi:10.1016/j.jpeds.2018.03.056

23. DeVita MA, Smith GB, Adam SK, et al. "Identifying the hospitalised patient in crisis"—a consensus conference on the afferent limb of rapid response systems. *Resuscitation*. 2010;81(4):375-382.

24. Howell MD, Ngo L, Folcarelli P, et al. Sustained effectiveness of a primary-team-based rapid response system. *Critical care medicine*. 2012;40(9):2562-2568. doi:10.1097/CCM.0b013e318259007b

25. Leppin AL, Gionfriddo MR, Kessler M, et al. Preventing 30-day hospital readmissions: a systematic review and meta-analysis of randomized trials. *JAMA internal medicine*. 2014;174(7):1095-1107.

26. Koff P, Jones RH, Cashman JM, Voelkel NF, Vandivier R. Proactive integrated care improves quality of life in patients with COPD. *European Respiratory Journal*. 2009;33(5):1031-1038.

27. Gorodeski EZ. Home-based care for heart failure. *OPTIMIZING HOME HEALTH CARE: ENHANCED VALUE AND IMPROVED OUTCOMES*. 2013;54:20.

28. Bylander J. Community-Focused Health Care For The Seriously Ill. 2019.

29. Burke RE, Coleman EA. Interventions to decrease hospital readmissions: keys for cost-effectiveness. *JAMA internal medicine*. 2013;173(8):695-698.

30. Feltner C, Jones CD, Cené CW, et al. Transitional care interventions to prevent readmissions for persons with heart failure: a systematic review and meta-analysis. *Annals of internal medicine*. 2014;160(11):774-784.

31. Coye MJ, Haselkorn A, DeMello S. Remote patient management: technology-enabled innovation and evolving business models for chronic disease care. *Health Affairs*. 2009;28(1):126-135.

32. Langsetmo L, Platt RW, Ernst P, Bourbeau J. Underreporting exacerbation of chronic obstructive pulmonary disease in a longitudinal cohort. *American journal of respiratory and critical care medicine*. 2008;177(4):396-401.

33. Inglis SC, Clark RA, Dierckx R, Prieto-Merino D, Cleland JG. Structured telephone support or non-invasive telemonitoring for patients with heart failure. *Cochrane Database of Systematic Reviews*. 2015;2015(10)

34. Wong FKY, Chow SKY, Chan TMF, Tam SKF. Comparison of effects between home visits with telephone calls and telephone calls only for transitional discharge support: a randomised controlled trial. *Age and ageing*. 2013;43(1):91-97.

35. Ritchie CS, Houston TK, Richman JS, et al. The E-Coach technology-assisted care transition system: a pragmatic randomized trial. *Translational behavioral medicine*. 2016;6(3):428-437.

36. De San Miguel K, Smith J, Lewin G. Telehealth remote monitoring for community-dwelling older adults with chronic obstructive pulmonary disease. *Telemedicine and e-Health*. 2013;19(9):652-657.

37. Kitsiou S, Paré G, Jaana M. Effects of home telemonitoring interventions on patients with chronic heart failure: an overview of systematic reviews. *Journal of medical Internet research*. 2015;17(3)

38. Ranganathan A, Dougherty M, Waite D, Casarett D. Can palliative home care reduce 30-day readmissions? Results of a propensity score matched cohort study. *Journal of palliative medicine*. Oct 2013;16(10):1290-3. doi:10.1089/jpm.2013.0213

39. Brian Cassel J, Kerr KM, McClish DK, et al. Effect of a Home-Based Palliative Care Program on Healthcare Use and Costs. *Journal of the American Geriatrics Society*. Nov 2016;64(11):2288-2295. doi:10.1111/jgs.14354

40. Durie C, Tanksley-Bowe C. Rural Readmissions in the Palliative Care Vacuum. *Journal of hospice and palliative nursing : JHPN : the official journal of the Hospice and Palliative Nurses Association*. Apr 2018;20(2):160-165. doi:10.1097/njh.0000000000000421

41. Nouryan CN, Morahan S, Pecinka K, et al. Home telemonitoring of community-dwelling heart failure patients after home care discharge. *Telemedicine and e-Health*. 2018;

42. Piña IL. Transitions of Care in Heart Failure: The Case for Comparative Effectiveness Research. *Current heart failure reports*. 2011;8(2):81-83.

43. Inglis SC, Conway A, Cleland JG, Clark RA. Is age a factor in the success or failure of remote monitoring in heart failure? Telemonitoring and structured telephone support in elderly heart failure patients. *European Journal of Cardiovascular Nursing*. 2015;14(3):248-255.

44. Graham J, Tomcavage J, Salek D, Sciandra J, Davis DE, Stewart WF. Postdischarge monitoring using interactive voice response system reduces 30-day readmission rates in a case-managed Medicare population. *Medical care*. 2012:50-57.

45. Gomes B, Calanzani N, Curiale V, McCrone P, Higginson IJ. Effectiveness and cost-effectiveness of home palliative care services for adults with advanced illness and their caregivers. *Cochrane Database of Systematic Reviews*. 2013;(6)

46. Cella D, Riley W, Stone A, et al. The Patient-Reported Outcomes Measurement Information System (PROMIS) developed and tested its first wave of adult self-reported health outcome item banks: 2005–2008. *Journal of clinical epidemiology*. 2010;63(11):1179-1194.

47. Cook KF, Jensen SE, Schalet BD, et al. PROMIS measures of pain, fatigue, negative affect, physical function, and social function demonstrated clinical validity across a range of chronic conditions. *J Clin Epidemiol*. May 2016;73:89-102. doi:10.1016/j.jclinepi.2015.08.038

48. Endicott J, Nee J, Harrison W, Blumenthal R. Quality of Life Enjoyment and Satisfaction Questionnaire: a new measure. *Psychopharmacology bulletin*. 1993;29(2):321-6.

49. Colantuoni E, Scharfstein DO, Wang C, et al. Statistical methods to compare functional outcomes in randomized controlled trials with high mortality. *BMJ*. Jan 3 2018;360:j5748. doi:10.1136/bmj.j5748

50. Liu Q, Shepherd BE, Li C, Harrell FE, Jr. Modeling continuous response variables using ordinal regression. *Statistics in medicine*. Nov 30 2017;36(27):4316-4335. doi:10.1002/sim.7433

51. Singh GK. Area deprivation and widening inequalities in US mortality, 1969–1998. *American journal of public health*. 2003;93(7):1137-1143.

52. Liu Y, Wang B, Yang M, et al. Correct and logical causal inference for binary and time-to-event outcomes in randomized controlled trials. *Biom J*. Feb 2022;64(2):198-224. doi:10.1002/bimj.202000202

53. Michael GA. A significance test of interaction in 2xK designs with proportions. *Tutorials in Quantitative Methods for Psychology*. 2007;3(1):1-7.

54. Kent DM, Steyerberg E, van Klaveren D. Personalized evidence based medicine: predictive approaches to heterogeneous treatment effects. *BMJ*. Dec 10 2018;363:k4245. doi:10.1136/bmj.k4245

55. Athey S, Tibshirani J, Wager S. Generalized random forests. *The Annals of Statistics*. 2019;47(2):1148-1178, 31.

56. Friedberg R, Tibshirani J, Athey S, Wager S. Local Linear Forests. *J Comput Graph Stat*. Nov 10 2021;30(2):503-517. doi:10.1080/10618600.2020.1831930

57. Tian L, Alizadeh AA, Gentles AJ, Tibshirani R. A Simple Method for Estimating Interactions Between a Treatment and a Large Number of Covariates. *Journal of the American Statistical Association*. 2014/10/02 2014;109(508):1517-1532. doi:10.1080/01621459.2014.951443

58. Nie X, Wager S. Quasi-oracle estimation of heterogeneous treatment effects. *Biometrika*. 2020;108(2):299-319. doi:10.1093/biomet/asaa076

59. Chernozhukov V, Demirer M, Duflo E, Fernández-Val I. Generic Machine Learning Inference on Heterogeneous Treatment Effects in Randomized Experiments, with an Application to Immunization in India. *National Bureau of Economic Research Working Paper Series*. 2018;No. 24678doi:10.3386/w24678

60. Talisa VB and Chanc CCH. Learning and confirming. Learning and confirming a class of treatment responders in clinical trials. *Stat Med*. Sep 30 2021; 40(22):4872-4889.

61. Shah FA, Talisa VB, Chang CCH, Triantafyllou S, Tang L, Mayr FB, Higgins AM, Peake SL, Mouncey P, Harrison DA, DeMerle KM, Kennedy JN, Cooper GF, Bellomo R, Rowan K, Yealy DM, Seymour CW, Angus DC, Yende S. Heterogeneity in the effect of early goal-directed therapy for septic shock: A secondary analysis of two multicenter international trials. Crit Care Med 2025 Jan 1; 53(1):e4-e14.

62. Buuren Sv. *Flexible Imputation of Missing Data*. Second ed. Chapman & Hall/CRC; 2018.

12. Appendix

12.1. *Sepsis data for simulations*

To create realistic clinical trial simulations, we obtained pre-trial member-level data from UPMC for patients that were hospitalized from either sepsis, pneumonia, and/or a lower respiratory infection. Patients in this sample were at least 21 years old and deemed low risk for readmission (based on an internal UPMC risk score). We received 4718 records from members that received their insurance through Medicare or commercial plans. This data included the following covariates: age group, CCI, sepsis status, pneumonia status, LRTI status, whether a patient had mechanical ventilation, vasopressors, or was in the ICU. In addition, we have whether a patient was discharged to a SNF or to home, as well as their home days endpoint score. We additionally received 2737 records from members whose insurance is through Medicaid or SNP (special needs population). This data only contained the home days endpoint with no additional covariate information.

Using the totality of this data, we summarize the home days distribution by finding the proportion in each level of the outcome, as shown in the “Overall” column in Figure 2.

In addition, we summarize the two strata variables using the partial data with covariate information. We define the severe strata as patients who were on mechanical ventilation or vasopressors during their hospital stay. In this data, 11% of the population meets this definition. We define the SNF population as patients whose discharge status was “Discharge to SNF”, where 37% of the population meet this definition. Using these covariates, we estimate the relationship between the home days endpoint and the covariates, illness severity and discharge to SNF, using a frequentist ordinal regression. We estimate an odds ratio for severe illness compared to non-severe of 0.75 and an odds ratio for patients discharged to SNF compared to those discharged home to be 0.35. Under these estimated odds ratios, we transform the home days distribution (using the full data) for the severe strata and the discharged to SNF strata. The resulting distributions are presented in Figure 2. Patients who are not categorized as severe or were discharged straight to home are assumed to have the baseline home days distribution shown in the overall column.

Strata variables were chosen empirically by evaluating the covariates that best explained patient heterogeneity in the data. These two variables were identified as major risk factors for this patient population using the provided data, therefore, were critical to control baseline characteristics.

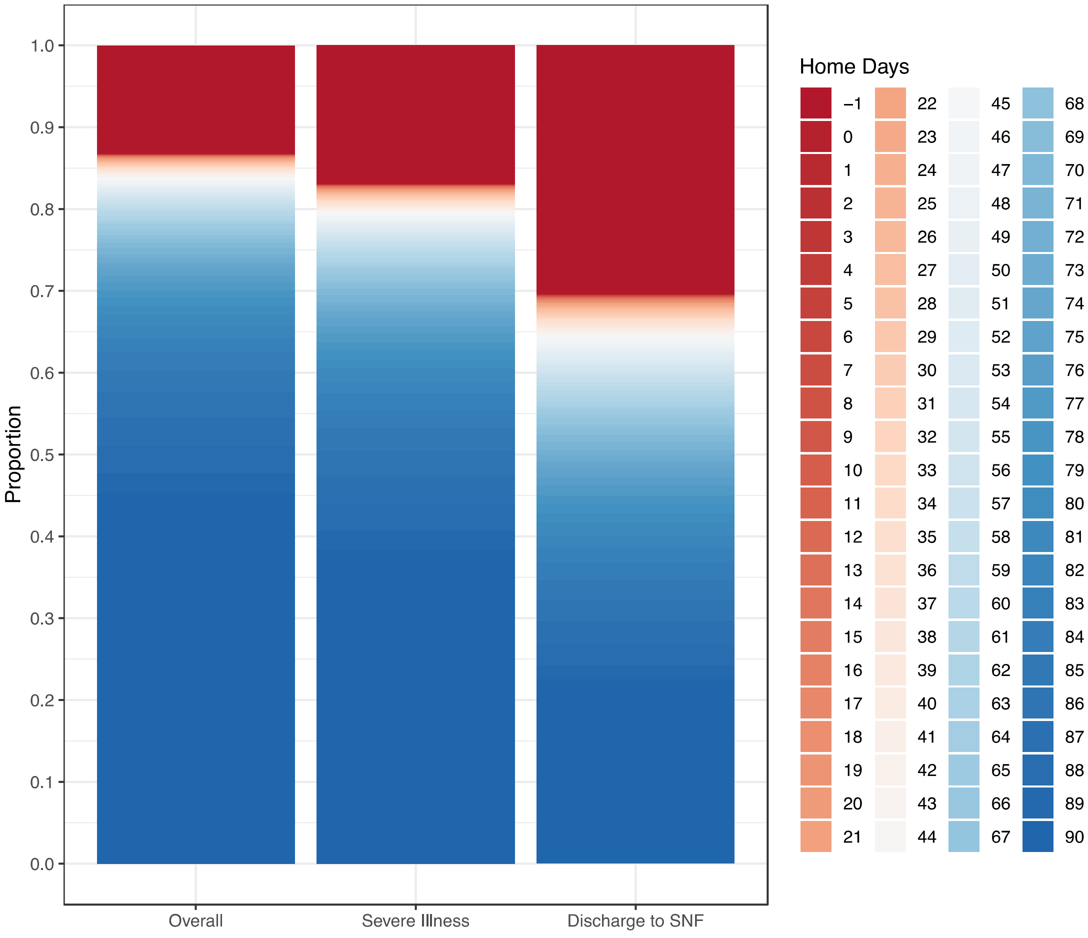

Figure 2: Distribution of home days endpoint using UPMC member level data.

12.2. *Virtual patient simulation*

In these simulations, we simulate a maximum total sample size of 1500 patients assuming no patient dropout. For each simulation, we assume the ACCOMPLISH trial will enroll 11% of patients that are in the severe strata and 37% of patients that are discharged to SNF (using proportions from the observed data). We simulate the strata by drawing from two independent Bernoulli variables with probability 11% for the severe strata variable and 37% for the SNF variable. These strata are not mutually exclusive such that a patient may be both severe and discharged to SNF. Based on a patient's strata assignment, we shift the overall home days distribution using the odds ratios of 0.75 and 0.35 for severe and discharged to SNF strata, respectively. If a patient is both severe and discharged to SNF, their home days distribution is shifted with an odds ratio of 0.26 ($\exp(\log(0.75) + \log(0.35))$). We do not incorporate the additional covariates (age categories) into the simulations since we do not expect age to influence the treatment effect and therefore the operating characteristics of the trial.

To simplify the simulations, we assume a collapsed distribution of home days. Table 2 shows the collapsed distribution for the baseline population.

Table 2. Collapsed home days distribution for the baseline population

Death	0-19	20-39	40-49	50-59	60-69	70-79	80-89	90
0.133	0.005	0.017	0.017	0.026	0.042	0.077	0.228	0.454

For patients in strata, this distribution is then shifted based on the ORs described above.

12.3. *Simulated treatment effect scenarios*

We assume proportional odds treatment effects for each care model under different patterns of efficacy. Table 3 presents the scenarios in terms of the OR on each of the 4 care models. Table 4 summarizes the endpoint for each stratum under each of these assumed OR treatment effects.

Table 3. Treatment effect scenarios

		RPM low + Standard	RPM high + Standard	RPM low + Enhanced	RPM high + Enhanced
**1**	Null	1.0	1.0	1.0	1.0
**2**	One Weak	1.0	1.0	1.0	1.25
**3**	One Expected	1.0	1.0	1.0	1.5
**4**	One Strong	1.0	1.0	1.0	1.75
**5**	Two Weak	1.0	1.25	1.0	1.25
**6**	Two Expected	1.0	1.5	1.0	1.5
**7**	Two Strong	1.0	1.75	1.0	1.75
**8**	All Weak	1.25	1.25	1.25	1.25
**9**	All Expected	1.5	1.5	1.5	1.5
**10**	All Strong	1.75	1.75	1.75	1.75
**11**	All Weak, Combo Additive	1.25	1.25	1.25	1.56
**12**	All Expected, Combo Additive	1.5	1.5	1.5	2.25
**13**	All Strong, Combo Additive	1.75	1.75	1.75	3.06

Table 4. Summary of PDHD distribution under different treatment effect scenarios*.

	Median PDHD	Probability of Mortality	Probability of 90 PDHDs
**OR = 1.00**			
Baseline	90 (82, 90)	0.13	0.45
Severe	88 (79, 90)	0.17	0.38
Discharged to SNF	84 (70, 90)	0.31	0.23
**OR = 1.25**			
Baseline	90 (84, 90)	0.11	0.51
Severe	90 (81, 90)	0.14	0.44
Discharged to SNF	86 (72, 90)	0.26	0.27
**OR = 1.50**			
Baseline	90 (85, 90)	0.09	0.55
Severe	90 (83, 90)	0.12	0.48
Discharged to SNF	97 (75, 90)	0.23	0.30
**OR = 1.75**			
Baseline	90 (86, 90)	0.08	0.59
Severe	90 (84, 90)	0.11	0.52
Discharged to SNF	87 (77, 90)	0.20	0.34

**For a clinical perspective, assume a length of stay of readmission is 5 days. To achieve an OR of 1.5 in the baseline group (nonsevere illness, discharged home), at least 25% of these patients must experience an increase in PDHD from 82 to 84, corresponding to ~0.5 fewer readmissions.*

12.4. *Accrual*

We simulate patient accrual from a Poisson process assuming an average accrual rate of 70 patients per month until the maximum sample size of 1500 with complete 90-day follow-up is achieved.

12.5. *Operating characteristics when assumptions are met*

For each of the scenarios described in Table 3, we simulate 1000 trials with and without the futility rule and characterize the behavior across trials. The null scenarios are summarized with 10,000 simulated trials.

Under each treatment effect scenario, we report the following operating characteristics in Table 5.1 for results without the futility rule and Table 5.2 for results with the futility rule:

- Probability of success overall and per arm (e.g., power or type I error)
- Probability of futility overall and per arm
- Mean number of subjects enrolled per arm and in total

- Probability each arm is selected as best
- Average bias for each arm (calculated as estimated OR (using posterior median) – true OR for each arm)

Tables 5.1 and 5.2 abbreviate the names of the interventions to 1-4, where 1 = RPM low/Standard, 2 = RPM high/Standard, 3 = RPM low/Enhanced, 4 = RPM high/Enhanced.

Under the null scenario (with and without futility), the overall simulated type I error (defined as any active arm declaring success in the null scenario) is about 2.3%. The average number of patients enrolled on each care model is between 249-268 patients and all arms have about an equal (~25%) probability of being declared the best care model. The average bias for each arm is -0.03 (similar regardless of futility).

The following discussion focuses on the results including the futility rule. Table 5.1 is there for reference, though notably we see essentially no power loss when the futility rule is used compared to no futility. Under the presented scenarios, trials rarely stop prior to the maximum sample size. The column summarizing the average total sample size of the trial is provided to understand the impact of the futility rule. Under the null scenario, the average total sample size is 1482 when the futility rule is used, with a 4.7% probability that the trial stops early for futility. Each arm has about a 25% chance of being stopped for futility at one of the interim analyses.

Rows 2-4 show scenarios where only intervention 4 (RPM enhanced/standard) is effective. In these scenarios, arm 4 enrolls on average 457, 607, and 695 patients under the three assumed treatment effect strengths. Arms 1, 2 and 3 enroll around 200 patients under the weak treatment effect, around 150 for the moderate treatment effect, and around 120 for the strong treatment effect. When there is a single effective intervention, the design appropriately enrolls more patients onto the stronger arm and reduces allocation to the weaker arms. This is also seen in the summary of the probability of selecting arm 4 as the best arm, which is around 99% when the treatment effect is expected or strong. The power for the effective arm (4) is 29.9% under a weak effect, 85.1% under the expected effect, and 98.6% under the strong effect. The type I error rate on the non-effective interventions (1, 3, 4) is less than 0.025. The overall power for these scenarios is 30.8%, 85.1%, and 98.7% under the weak, expected, and strong treatment effect, respectively. The average bias is minimal for these scenarios with all arms observing bias less than 0.10 in both directions for all treatment effect strengths.

In scenarios 5-7 when interventions 2 and 4 are effective, the overall power increases to 41.7%, 91.8%, and 99.5% under the weak, expected, and strong treatment effect, respectively. The per arm power is 24.9% for arm 2 and 27.3% for arm 4 under a weak treatment effect, 71.2% for arm 2 and 72.5% for arm 4 under the expected treatment effect, and 90.4% and 92.2% for arm 2 and 4 under the strong treatment effect. The ineffective arms (1 and 3) have type I errors that are less than 2.5%. The average bias is minimal for all arms under all treatment effects.

In scenarios 8-10 when the treatment effects are the same across all arms, allocation is consistent across the care models, with around 250-270 patients per arm. The probability of being selected as the best arm is also approximately 25% for each of the four care models. Power per arm under the expected treatment effect is about 60%, and the overall probability of finding a successful arm (overall power) is 95%. Power per arm under the strong treatment effect is about 80%, and the overall probability of finding a successful arm (overall power) is 99.9%.

In the final scenario, when all arms are effective, but intervention 4 has an additive benefit, power for arm 4 is 81% under the weak treatment effect and greater than 99% for the expected and strong treatment effect. The probability of being superior to STS (power) for Arm 2 and 3 are about 20% for the weak effect, 50% for the expected effect and about 76% for the strong effect. Arm 1 has about 20-30% lower power in this scenario compared to arms 2 and 3 despite the arms all being simulated with the same treatment effect. The model underestimates the effect of arm 1, with an average bias between -0.07 in weak scenario to -0.16 in the strong scenario. Overall power for these scenarios is 86.5% under the weak treatment effect and 100% for both the expected and strong treatment effect. The design appropriately finds the best arm 85% of the time under the weak effect and about 99% of the time under the expected and strong treatment effect.

Table 5.1 Operating characteristics per intervention without futility

Scenario		Mean N						Success					Prob Selected Best				Bias			
		STS	1	2	3	4	Total	1	2	3	4	Overall	1	2	3	4	1	2	3	4
**1**	Null	450	253	262	266	266	1500	0.005	0.006	0.007	0.007	0.023	0.247	0.244	0.258	0.251	-0.03	-0.03	-0.02	-0.03
**2**	One Weak	450	178	206	208	458	1500	0.006	0.009	0.011	0.299	0.308	0.038	0.056	0.052	0.854	-0.04	0.01	0.01	-0.01
**3**	One Expected	450	130	156	158	607	1500	0.001	0.014	0.006	0.847	0.847	0.006	0.002	0.001	0.991	-0.06	0.03	0.04	-0.01
**4**	One Strong	450	104	128	124	694	1500	0.004	0.009	0.013	0.987	0.988	0.001	0	0	0.999	0.06	-0.02	-0.07	0.05
**5**	Two Weak	450	157	358	160	375	1500	0.009	0.252	0.007	0.269	0.416	0.014	0.472	0.012	0.502	-0.01	-0.02	-0.01	-0.02
**6**	Two Expected	450	109	410	111	420	1500	0.004	0.713	0.003	0.726	0.919	0.002	0.473	0	0.525	0.00	-0.02	-0.01	-0.02
**7**	Two Strong	450	91	427	92	440	1500	0.002	0.903	0.004	0.923	0.996	0	0.485	0	0.515	0.00	-0.02	-0.02	-0.02
**8**	All Weak	450	253	258	266	273	1500	0.189	0.188	0.213	0.213	0.498	0.244	0.246	0.268	0.242	-0.03	-0.03	-0.02	-0.02
**9**	All Expected	450	245	267	266	272	1500	0.562	0.595	0.601	0.603	0.952	0.217	0.274	0.257	0.252	-0.05	-0.04	-0.03	-0.04
**10**	All Strong	450	243	262	272	272	1500	0.823	0.817	0.852	0.843	0.999	0.231	0.236	0.274	0.259	-0.04	-0.04	-0.02	-0.03
**11**	All Weak, Combo Additive	450	175	206	210	458	1500	0.113	0.206	0.215	0.808	0.864	0.053	0.052	0.047	0.848	-0.07	0.01	0.01	-0.03
**12**	All Expected, Combo Additive	450	125	163	162	599	1500	0.301	0.554	0.551	0.997	1.000	0.004	0.004	0.006	0.986	-0.12	0.04	0.04	-0.02
**13**	All Strong, Combo Additive	450	106	132	134	678	1500	0.500	0.778	0.801	1.000	1.000	0.000	0.000	0.000	1.000	-0.16	0.09	0.11	-0.02

Table 5.2 Operating characteristics per intervention with futility

Scenario		Mean N						Success/Futility					Prob Selected Best				Bias			
		STS	1	2	3	4	Total	1	2	3	4	Overall	1	2	3	4	1	2	3	4
1	Null	451	249	257	262	263	1482	0.006/0.252	0.006/0.257	0.006/0.249	0.006/0.267	0.023/0.047	0.247	0.24	0.261	0.252	-0.03	-0.03	-0.03	-0.03
2	One Weak	450	176	204	205	457	1492	0.006/0.270	0.009/0.186	0.011/0.175	0.299/0.055	0.308/0.018	0.042	0.053	0.057	0.848	-0.05	0.01	0.01	-0.02
3	One Expected	450	128	156	157	607	1498	0.001/0.279	0.014/0.144	0.006/0.131	0.851/0.011	0.851/0.006	0.007	0.003	0.001	0.989	-0.06	0.03	0.04	-0.01
4	One Strong	450	104	128	124	695	1500	0.004/0.321	0.009/0.120	0.013/0.135	0.986/0.006	0.987/0.004	0.001	0	0	0.999	-0.08	0.06	0.05	-0.01
5	Two Weak	451	156	357	158	375	1496	0.009/0.202	0.249/0.047	0.007/0.197	0.273/0.043	0.417/0.010	0.019	0.468	0.01	0.503	-0.01	-0.02	-0.01	-0.01
6	Two Expected	450	109	410	111	420	1500	0.004/0.180	0.712/0.004	0.003/0.192	0.725/0.003	0.918/0.002	0.002	0.476	0	0.522	0	-0.02	-0.01	-0.02
7	Two Strong	450	91	427	92	440	1500	0.002/0.180	0.904/0.004	0.004/0.193	0.922/0.003	0.995/0.002	0	0.486	0	0.514	0	-0.02	-0.01	-0.02
8	All Weak	450	253	258	266	272	1499	0.190/0.034	0.190/0.041	0.213/0.039	0.213/0.050	0.500/0.004	0.243	0.248	0.267	0.242	-0.03	-0.03	-0.02	-0.02
9	All Expected	450	245	267	266	272	1500	0.562/0.016	0.595/0.006	0.601/0.020	0.603/0.009	0.952/0.001	0.217	0.274	0.257	0.252	-0.05	-0.04	-0.04	-0.04
10	All Strong	450	243	262	272	272	1500	0.823/0.003	0.817/0.004	0.852/0.002	0.843/0.002	0.999/0.000	0.231	0.236	0.274	0.259	-0.04	-0.04	-0.02	-0.03
11	All Weak, Combo Additive	450	175	206	210	458	1500	0.113/0.051	0.205/0.037	0.216/0.036	0.808/0.013	0.865/0.001	0.052	0.052	0.048	0.848	-0.07	0.01	0.01	-0.03
12	All Expected, Combo Additive	450	125	163	162	599	1500	0.301/0.017	0.554/0.008	0.551/0.011	0.997/0.002	1.000/0.001	0.004	0.004	0.006	0.986	-0.12	0.04	0.04	-0.02
13	All Strong, Combo Additive	450	106	132	134	678	1500	0.500/0.007	0.778/0.004	0.801/0.002	1.000/0.000	1.000/0.000	0	0	0	1	-0.16	0.09	0.11	-0.02

Power Calculations 11/24/2023

There is a possibility that full enrollment will not be feasible. In this section we illustrate the power that may be obtained in each of the scenarios above at sample sizes of 1200 to 1500 complete, with 1500 complete replicating the results shown above in the “with futility” tables. For the N=1500 column, taken from the table above, the results were computed via trial simulation. For the remaining columns, approximations were employed reflecting the information

fractions available at 1200 to 1450 complete patients. We expect these approximations to be close, but they are likely to be slightly aggressive, meaning that if they are off they are likely to overestimate power. We do not expect needed sample sizes to be off by more than ~25 patients.

Note that similar to the above tables, the table below shows sample sizes of complete patients. At present, the trial is observing 2.5% of patients lost to followup, significantly fewer than originally estimated. Nevertheless, to obtain a given number of complete patients, we must enroll a larger number. For example, to obtain 1350 complete patients with 2.5% loss to followup, we need to enroll 1350/(1-0.025) = 1385 patients total.

Scenario	N=1200	N=1250	N=1300	N=1350	N=1400	N=1450	N=1500	N Needed for 80% power (if feasible)
1, Null	~0.025	~0.025	~0.025	~0.025	~0.025	~0.025	0.023	NA
2, One Weak	0.256	0.265	0.274	0.282	0.291	0.299	0.308	NA
3, One Expected	0.765	0.782	0.798	0.812	0.826	0.839	0.851	1308
4, One Strong	0.963	0.969	0.974	0.978	0.981	0.984	0.987	<1000
5, Two Weak	0.347	0.359	0.371	0.382	0.394	0.406	0.417	NA
6, Two Expected	0.850	0.864	0.877	0.889	0.899	0.909	0.918	1049
7, Two Strong	0.982	0.985	0.988	0.990	0.992	0.994	0.995	<1000

8, All weak	0.418	0.432	0.446	0.460	0.474	0.487	0.500	NA
9, All Expected	0.900	0.911	0.921	0.930	0.938	0.946	0.952	<1000
10, All Strong	0.994	0.996	0.997	0.998	0.998	0.999	0.999	<1000
11, All weak combo additive	0.782	0.798	0.814	0.828	0.841	0.854	0.865	1255
12, All expected combo additive	>0.997	>0.998	>0.998	>0.999	>0.999	>0.999	1.000	<1000
13, All strong, combo additive	>0.997	>0.998	>0.998	>0.999	>0.999	>0.999	1.000	<1000

12.6 Operating characteristics when assumptions are violated

As noted in Section 8.5, it is possible that the interventions have differential effects on patients who would have died within 90 days had they been assigned to SRS compared to those who would have survived. This type of differential effect of treatment for different types of patients violates the proportional odds assumption from the ordinal logistic regression model. Therefore, we provide operating characteristics for simulations in which patient data are generated under non-proportional assumptions. In these scenarios, we leave the probability of death unchanged regardless of the treatment effect and shift the remaining portion of the home-days endpoint (0-90) by the scenario specific OR. See Figure 2 for a visual representation of the treatment effect on the baseline probability distribution under these non-proportional assumptions.

Table 6 shows the population characteristics under a range of odds ratios for these two scenarios, for each of the 3 population subsets. Odds ratios in Table t reflect the effect of interventions on home days among survivors only.

Table 6. Summary of PDHD distribution under different treatment effect scenarios*, when proportionality of effect of intervention is violated

	Median PDHD Among Survivors (IQR)	Probability of Mortality	Probability of 90 PDHDs
**OR=1.00**			
Baseline	90 (82, 90)	0.13	0.45
Severe	88 (79, 90)	0.17	0.38
Discharged to SNF	84 (70,90)	0.30	0.23
**OR=1.25**			
Baseline	90 (84, 90)	0.13	0.50
Severe	90 (82, 90)	0.17	0.43
Discharged to SNF	86 (74, 90)	0.30	0.26
**OR=1.50**			
Baseline	90 (85, 90)	0.13	0.54
Severe	90 (84, 90)	0.17	0.47
Discharged to SNF	87 (76, 90)	0.30	0.29
**OR=1.75**			
Baseline	90 (86, 90)	0.13	0.57

Severe	90 (85, 90)	0.17	0.50
Discharged to SNF	88 (79, 90)	0.30	0.32

**For a clinical perspective, assume a length of stay of readmission is 5 days. To achieve an OR of 1.5 in the baseline group (non-severe illness, discharged home), at least 25% of these patients must experience an increase in PDHD from 82 to 84, corresponding to ~0.5 fewer readmissions.*

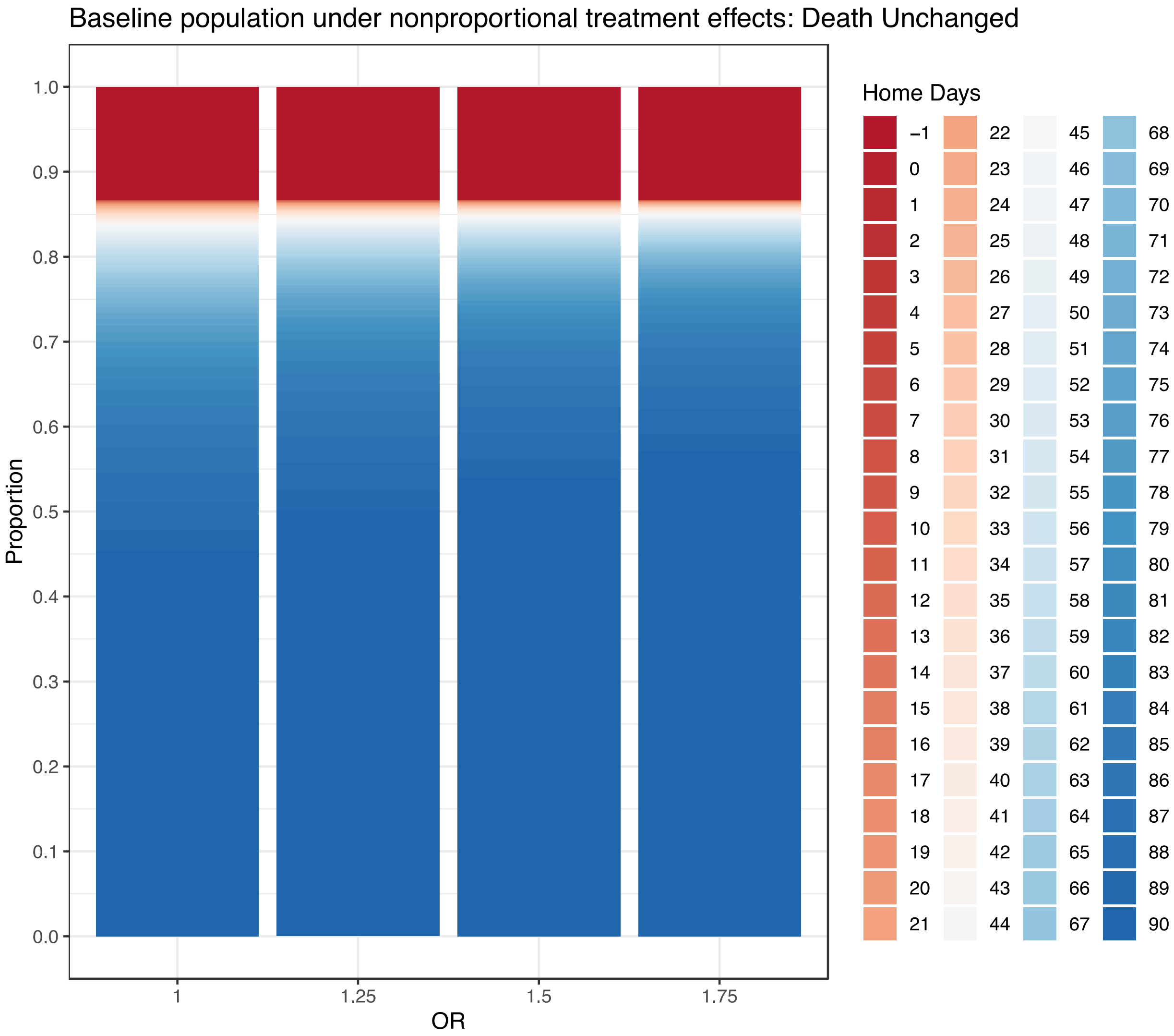

Figure 3: Distribution of home days endpoint using UPMC member level data showing non-proportional shifts where the probability of death is unchanged and the effect of the OR is applied only to 0-90 values on the scale.

For all scenarios outlined in Table 5, we repeat the simulations under these non-proportional assumptions. The simulations are summarized in the same manner as above in Tables 7.1 and 7.2, displaying results without futility and with futility, respectively.

Table 7.1 Operating characteristics per intervention without futility under non-proportional assumptions

Scenario		Mean N						Success					Prob Selected Best				Bias			
		STS	1	2	3	4	Total	1	2	3	4	Overall	1	2	3	4	1	2	3	4
**1**	Null	450	253	262	266	266	1500	0.005	0.006	0.007	0.007	0.023	0.247	0.244	0.258	0.251	-0.03	-0.03	-0.02	-0.03
**2**	One Weak	450	206	233	236	376	1500	0.002	0.006	0.01	0.085	0.095	0.107	0.124	0.11	0.659	-0.04	-0.01	0	-0.13
**3**	One Expected	450	174	201	202	472	1500	0.005	0.012	0.003	0.338	0.341	0.05	0.052	0.041	0.857	-0.05	0	0	-0.25
**4**	One Strong	450	143	178	173	556	1500	0.001	0.013	0.01	0.615	0.617	0.006	0.014	0.015	0.965	-0.06	0.02	0.02	-0.38
**5**	Two Weak	450	190	331	196	333	1500	0.005	0.078	0.005	0.083	0.146	0.061	0.429	0.054	0.456	-0.02	-0.13	-0.02	-0.12
**6**	Two Expected	450	149	374	152	376	1500	0.004	0.287	0.003	0.287	0.473	0.019	0.501	0.008	0.472	-0.01	-0.25	-0.02	-0.26
**7**	Two Strong	451	121	396	125	408	1500	0.004	0.543	0.003	0.555	0.774	0	0.476	0.001	0.523	-0.01	-0.38	-0.01	-0.37
**8**	All Weak	449	256	262	267	266	1500	0.07	0.060	0.060	0.076	0.208	0.255	0.244	0.252	0.249	-0.13	-0.14	-0.13	-0.13
**9**	All Expected	450	257	261	265	267	1500	0.221	0.231	0.238	0.232	0.581	0.258	0.243	0.263	0.236	-0.26	-0.26	-0.25	-0.26
**10**	All Strong	450	253	258	270	268	1500	0.404	0.410	0.421	0.423	0.838	0.241	0.236	0.266	0.257	-0.4	-0.4	-0.39	-0.4
**11**	All Weak, Combo Additive	450	209	233	236	373	1500	0.07	0.071	0.073	0.341	0.423	0.135	0.117	0.127	0.621	-0.15	-0.12	-0.11	-0.29
**12**	All Expected, Combo Additive	451	174	205	211	458	1500	0.141	0.21	0.222	0.838	0.903	0.046	0.043	0.059	0.852	-0.31	-0.24	-0.23	-0.69
**13**	All Strong, Combo Additive	450	153	183	195	518	1500	0.197	0.374	0.398	0.954	0.989	0.024	0.024	0.031	0.921	-0.47	-0.36	-0.35	-1.23

Table 7.2 Operating characteristics per intervention with futility under non-proportional assumptions

Scenario		Mean N						Success/Futility					Prob Selected Best				Bias			
		STS	1	2	3	4	Total	1	2	3	4	Overall	1	2	3	4	1	2	3	4
1	Null	451	249	257	262	263	1482	0.006/ 0.252	0.006/ 0.257	0.006/ 0.249	0.006/ 0.267	0.023/ 0.047	0.247	0.24	0.261	0.252	-0.03	-0.03	-0.03	-0.03
2	One Weak	450	201	229	233	375	1487	0.002/ 0.281	0.006/ 0.211	0.01/ 0.186	0.087/ 0.109	0.097/ 0.034	0.114	0.122	0.119	0.645	-0.04	-0.01	0	-0.13
3	One Expected	451	173	200	200	473	1497	0.005/ 0.253	0.012/ 0.164	0.003/ 0.163	0.342/ 0.051	0.345/ 0.011	0.052	0.052	0.039	0.857	-0.05	0	0	-0.26
4	One Strong	452	141	177	172	556	1498	0.001/ 0.295	0.013/ 0.156	0.01/ 0.166	0.622/ 0.022	0.624/ 0.008	0.007	0.015	0.016	0.962	-0.07	0.02	0.02	-0.38
5	Two Weak	452	188	330	194	332	1497	0.005/ 0.233	0.077/ 0.090	0.005/ 0.234	0.083/ 0.101	0.145/ 0.015	0.059	0.433	0.059	0.449	-0.02	-0.13	-0.02	-0.13
6	Two Expected	450	149	374	151	377	1500	0.004/ 0.192	0.287/ 0.030	0.003/ 0.211	0.290/ 0.032	0.475/ 0.003	0.02	0.499	0.008	0.473	-0.01	-0.25	-0.02	-0.26
7	Two Strong	450	120	396	124	407	1498	0.004/ 0.171	0.544/ 0.016	0.003/ 0.193	0.554/ 0.020	0.775/ 0.003	0	0.479	0.001	0.52	-0.01	-0.38	-0.01	-0.37
8	All Weak	450	255	261	265	265	1496	0.069/ 0.084	0.06/ 0.087	0.060/ 0.079	0.075/ 0.090	0.206/ 0.008	0.251	0.249	0.252	0.248	-0.13	-0.14	-0.13	-0.13
9	All Expected	450	257	261	265	267	1500	0.220/ 0.037	0.231/ 0.028	0.237/ 0.027	0.232/ 0.040	0.579/ 0.001	0.256	0.244	0.264	0.236	-0.26	-0.26	-0.25	-0.26
10	All	450	252	258	270	268	1499	0.403/ 0.030	0.41/ 0.029	0.422/ 0.028	0.423/ 0.028	0.837/ 0.003	0.242	0.235	0.267	0.256	-0.4	-0.4	-0.39	-0.4

	Strong																			
**11**	All Weak, Combo Additive	450	208	233	235	373	1499	0.07/ 0.095	0.071/ 0.064	0.073/ 0.066	0.341/ 0.035	0.423/ 0.004	0.134	0.12	0.125	0.621	-0.15	-0.12	-0.11	-0.29
**12**	All Expected, Combo Additive	451	174	205	211	458	1500	0.141/ 0.041	0.212/ 0.030	0.221/ 0.024	0.839/ 0.010	0.903/ 0.002	0.046	0.044	0.058	0.852	-0.31	-0.24	-0.23	-0.69
**13**	All Strong, Combo Additive	450	153	183	195	518	1500	0.197/ 0.031	0.374/ 0.019	0.398/ 0.015	0.954/ 0.006	0.989/ 0.003	0.024	0.024	0.031	0.921	-0.47	-0.36	-0.35	-1.23
