# Supplementary material for: Remote Monitoring Approaches to Reduce Readmissions After Infection and Sepsis: A Randomized Clinical Trial
Source: JAMA Netw Open. 2026 Jun 11;9(6):e2616641. doi: 10.1001/jamanetworkopen.2026.16641 (PMC13261490; doi:10.1001/jamanetworkopen.2026.16641)
Supplement: Supplement 2. — eMethods. Remote Patient Monitoring, Qualitative Analyses, and Post Hoc Subgroup Analyses eFigure 1. Number of Patients Randomized to Each Arm eFigure 2. Number of Patients Randomized Over Time eFigure 3. Cumulative Probability Density Plots for Postdischarge Home Days by Intervention Arm eFigure 4. Histograms of Postdischarge Home Days Outcomes by Intervention Arm eFigure 5. Relationship Between Cumulative Odds Ratios and Absolute Differences in PDHDs eFigure 6. Sensitivity Analysis of the Dichotomized Categories of PDHD eFigure 7. Marginal Effects of RPM Intensity and Care Team Compared With Usual Care (UC) on Postdischarge Home Days eFigure 8. Adjusted Hazard Ratios for Readmission Accounting for Competing Risk of Death by Intervention Arm eFigure 9. Cumulative Incidence of Readmission Accounting for Competing Risk of Death by Intervention Arm eTable 1. Criteria to Identify Sepsis, Respiratory Tract Infection, and COVID-19 eTable 2. Variables and Their Characteristics Used in the Predictive Model to Estimate the Risk of Readmission eTable 3. Exclusion Criteria eTable 4. Questionnaires Used for Remote Monitoring eTable 5. Secondary End Points eTable 6. Power Calculations at the Onset of the Trial for Different Scenarios eTable 7. Revised Power Calculation Prior to Resizing in November 2023 eTable 8. Baseline Characteristics by RPM Intensity and Team Composition eTable 9. Fidelity and Engagement of Patients in the Remote Monitoring Pathways or Questionnaires eTable 10. Fidelity and Engagement for the 328 Patients Assigned to Enhanced Care Team eTable 11. Effects of Study Interventions Compared With Usual Care on Secondary Outcomes eTable 12. Results of Subgroup and Sensitivity Analyses eTable 13. Probability of Differential Treatment Response Across Prespecified Subgroups eTable 14. Mortality, Hospital Readmission, and Hospice Admission Outcomes Stratified by Age Group eTable 15. Mortality, Hospital Readmission, and Hospice Admission Outcomes Stratified by Discharg [file jamanetwopen-e2616641-s002.pdf]

Supplementary Online Content

Yende S, Talisa VB, Mayes K, et al. Remote monitoring approaches to reduce readmissions after infection and sepsis: a randomized clinical trial. *JAMA Netw Open.* 2026;9(6):e2616641. doi:10.1001/jamanetworkopen.2026.16641

eMethods. Remote Patient Monitoring, Qualitative Analyses, and Post Hoc Subgroup Analyses

eFigure 1. Number of Patients Randomized to Each Arm

eFigure 2. Number of Patients Randomized Over Time

eFigure 3. Cumulative Probability Density Plots for Postdischarge Home Days by Intervention Arm

eFigure 4. Histograms of Postdischarge Home Days Outcomes by Intervention Arm

eFigure 5. Relationship Between Cumulative Odds Ratios and Absolute Differences in PDHDs

eFigure 6. Sensitivity Analysis of the Dichotomized Categories of PDHD

eFigure 7. Marginal Effects of RPM Intensity and Care Team Compared With Usual Care (UC) on Postdischarge Home Days

eFigure 8. Adjusted Hazard Ratios for Readmission Accounting for Competing Risk of Death by Intervention Arm

eFigure 9. Cumulative Incidence of Readmission Accounting for Competing Risk of Death by Intervention Arm

eTable 1. Criteria to Identify Sepsis, Respiratory Tract Infection, and COVID-19

eTable 2. Variables and Their Characteristics Used in the Predictive Model to Estimate the Risk of Readmission

eTable 3. Exclusion Criteria

eTable 4. Questionnaires Used for Remote Monitoring

eTable 5. Secondary End Points

eTable 6. Power Calculations at the Onset of the Trial for Different Scenarios

eTable 7. Revised Power Calculation Prior to Resizing in November 2023

eTable 8. Baseline Characteristics by RPM Intensity and Team Composition

© 2026 Yende S et al. *JAMA Network Open*.

eTable 9. Fidelity and Engagement of Patients in the Remote Monitoring Pathways or Questionnaires

eTable 10. Fidelity and Engagement for the 328 Patients Assigned to Enhanced Care Team

eTable 11. Effects of Study Interventions Compared With Usual Care on Secondary Outcomes

eTable 12. Results of Subgroup and Sensitivity Analyses

eTable 13. Probability of Differential Treatment Response Across Prespecified Subgroups

eTable 14. Mortality, Hospital Readmission, and Hospice Admission Outcomes Stratified by Age Group

eTable 15. Mortality, Hospital Readmission, and Hospice Admission Outcomes Stratified by Discharge Location

eTable 16. Characteristics of the Cohort Participating in Qualitative Analyses

eTable 17. Illustrative Quotes From Qualitative Analyses

This supplementary material has been provided by the authors to give readers additional information about their work.

© 2026 Yende S et al. *JAMA Network Open*.

eMethods. Remote Patient Monitoring, Qualitative Analyses, and Post Hoc Subgroup Analyses

Remote Patient Monitoring

Aspects of all intervention arms were in use by patients with complex care needs prior to execution of the trial at UPMC. The remote monitoring questionnaires were delivered through and/or supported by UPMC's existing remote patient monitoring (RPM) platform. Each intervention is designed to facilitate symptom monitoring, identification of worsening disease, and adherence to follow up care recommendations. RPM interventions started when the patient returned home, regardless of whether they had a temporary skilled nursing facility stay between hospital and home or not. Patients answered RPM questions while at home only. A summary of the study arms is included below and in Table S4:

- Usual care consisted of post-discharge assessment, education, and medication reconciliation delivered telephonically by a health plan case manager, home care as needed, and follow-up with the primary care within seven days post-discharge.

- Low-intensity Remote Patient Monitoring (RPM-Low):[74] Questions were pushed to patients twice a week for up to 90 days post-discharge (Table S4). Questions included those indicating worsening of infection, general health issues, common symptoms in patients who are recovering from a hospitalization for infection and sepsis, medications, use of emergency care, and satisfaction with end-of-life care. Additionally, patients had access to educational materials. Patient answers to RPM questions triggered high or medium alerts, which trigger a response by members of the intervention care team (see below).

- High-intensity Remote Patient Monitoring (RPM-High): Questions were pushed to members twice per week for up to 90 days post-discharge (Table S4). Questions included all questions in RPM-low but also asked about worsening of underlying heart or lung conditions, such as weight

© 2026 Yende S et al. *JAMA Network Open*.

gain or shortness of breath. Patient answers to RPM questions triggered high or medium alerts, which trigger a response by members of the intervention care team.

- Standard Team: RPM alerts were screened by a nurse-staffed call center. Nurses determined whether emergency care is needed. If not, nurses contacted the patient and/or the patients' primary care physician (PCP) or specialist to coordinate care and ensure timely follow-up.

- Enhanced Team: RPM alerts were screened by a nurse-staffed call center. Nurses determine whether emergency care is needed. If not, the call center alerted a multidisciplinary care team that is led by a certified registered nurse practitioner (CRNP). CRNPs had prescribing authority and training in a palliative care. In addition to reacting to RPM triggers, team members (e.g., CRNP, social workers, nurses) meet with the patient in-person or virtually in the week after discharge and at least twice more in the next 90 days, conducted assessments and a pharmacy review, develop care plans, and complete a POLST (physician orders for life-sustaining treatment) or a Pennsylvania Advance Directive.

Qualitative analyses

From September 2021 through July 2024, we conducted semi-structured telephone interviews with a subset of patients. All patients were eligible to be interviewed following the 90-day intervention period and outreach for participation was conducted by phone or email. We sought to purposively sample 20 to 25 patients from each intervention arm with some having a readmission within 60 days. We also aimed to stratify per intervention arm based on level of engagement (>/< 60% completion of questionnaires), severity of illness (ventilated or on vasopressors/inotropes during index hospitalization), and racial/ethnic diversity. Patient interviews were conducted as soon as possible following the 90-day intervention period to maximize study recall. The study team developed a semi-

© 2026 Yende S et al. *JAMA Network Open*.

structured interview guide which included questions related to participants' experiences with, opinions, and impact of RPM.

The qualitative component of the study was approved by the University of Pittsburgh Institutional Review Board. Consent for interview outreach was included in the general consent for the larger study with information related to participation and verbal consent obtained prior to starting the interviews. Participants received $25 compensation for completing an interview.

The interview questions relevant to the results included: 1) How confident did you feel logging in to see the remote patient monitoring questions; 2) How confident did you feel answering the remote patient monitoring questions you received; 3) Tell me about anything that you liked about using the monitoring (did not like); 4) During the study, were you ever contacted by a nurse because of one of your responses to the monitoring questions? (If yes, what information did the nurse provide about what you needed to do next?); 5) Were there any advantages to participating in remote patient monitoring after you were discharged from the hospital.

Interviews were conducted by trained qualitative PhD-level researchers (KR, JB) who had no prior relationships with the trial participants. Interviewers were audio recorded, transcribed, de-identified and uploaded to NVivo 13 software (2020, R1) for thematic analysis. Two qualitative researchers (KR, JB) iteratively reviewed transcripts to develop a hybrid inductive-deductive thematic codebook. The codebook included domains and themes, subthemes, definitions, and inclusion and exclusion criteria. Once an acceptable inter-rater reliability (kappa > 0.7, % agreement > 95%) was achieved, the transcripts were individually coded, and coding consistency was reviewed at predetermined intervals of the data (every 25%). For this paper, themes were summarized related to user engagement and impacts.

© 2026 Yende S et al. *JAMA Network Open*.

Post-hoc subgroup analyses

Predicted enrollment in remote monitoring

A total of 529 patients assigned to a remote monitoring enrolled to receive the intervention, and 358 chose not to enroll. Another 399 patients were assigned to usual care. A regression forest model (R package grf) was fit to the 887 RPM patients to predict enrollment status using the following baseline variables: age, sex, race, Charlson Comorbidity Index, comfort with technology, QLES score, PROMIS score, ADI state ranking, discharge to SNF, severe illness status, admission diagnosis, health plan membership, and whether the patient reported living alone. Missing data were handled internally by the regression forest algorithm. Overlap was examined by plotting the distribution of predicted probability to enroll separately for those who did and did not sign the EULA. The model was then used to predict enrollment for all N=1286 patients in the ACCOMPLISH primary analysis cohort, which was classified into “unlikely” and “likely” to enroll based on the predicted probabilities, using a threshold of 0.5. Cumulative logit models were then fit to estimate treatment effects within the unlikely and likely enrollment groups, as well as the interaction between group and treatment assignment. Models were equivalent to those fit to estimate pre-specified subgroup effects and interactions.

Enrollment era

We divided the enrollment period, which began in April 2021 as the first COVID wave was beginning in the United States, into two eras: during COVID surges (through January 2022 and marked by the Delta [mid-2021] and Omicron [late 2021 to early 2022] variant surges) and the post-COVID surges (February 2022 and after). Effects of RPM strategies on post-discharge home days compared to usual care were estimated similar to other subgroups, with interaction terms in a Bayesian cumulative proportional odds model and adjusting for discharge location, severity of illness, and quarter of calendar time. Other outcomes were described by arm and enrollment era.

© 2026 Yende S et al. *JAMA Network Open*.

eFigure 1. Number of Patients Randomized to Each Arm

Figure shows the total number of randomized to each arm – remote patient monitoring low intensity/standard team (standard low), remote patient monitoring high intensity/standard team (standard high), remote patient monitoring low intensity/enhanced team (enhanced low), and remote patient monitoring high intensity/enhanced team (enhanced high).

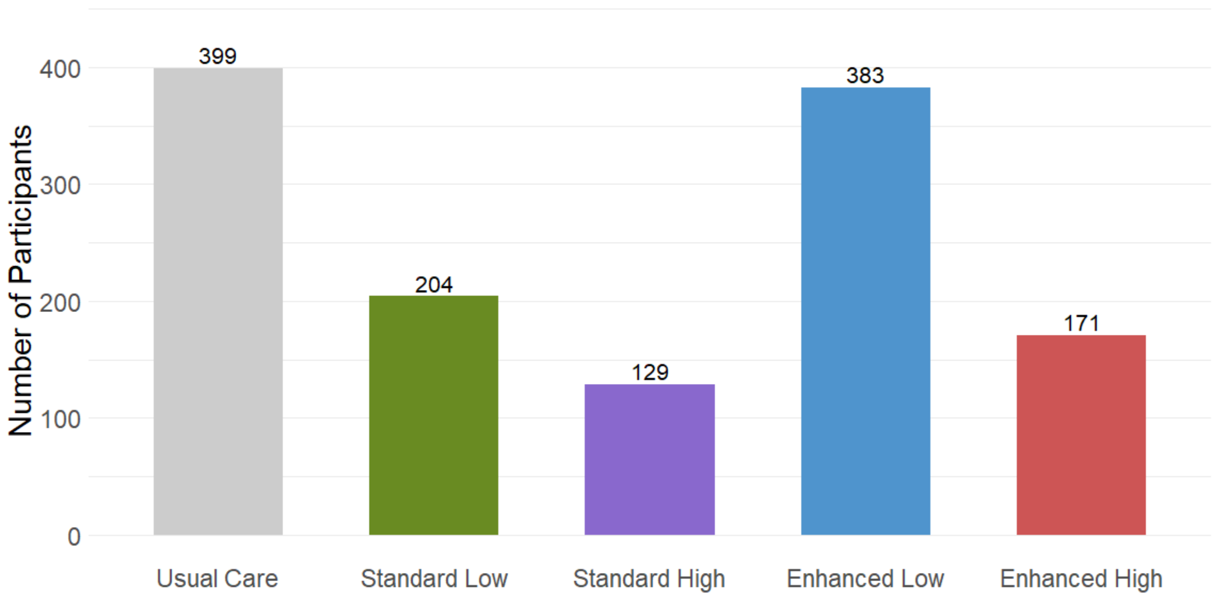

© 2026 Yende S et al. *JAMA Network Open*.

eFigure 2. Number of Patients Randomized Over Time

Total number of patients and by intervention arm randomized over time and distribution within each time epochs used to estimate time trends in the analysis model. The time epochs were derived so that the first bucket is the 3 months (12 weeks) from the earliest randomization date for a patient in the dataset. Thereafter, each bucket is defined as the next 3-month interval forwards in time.

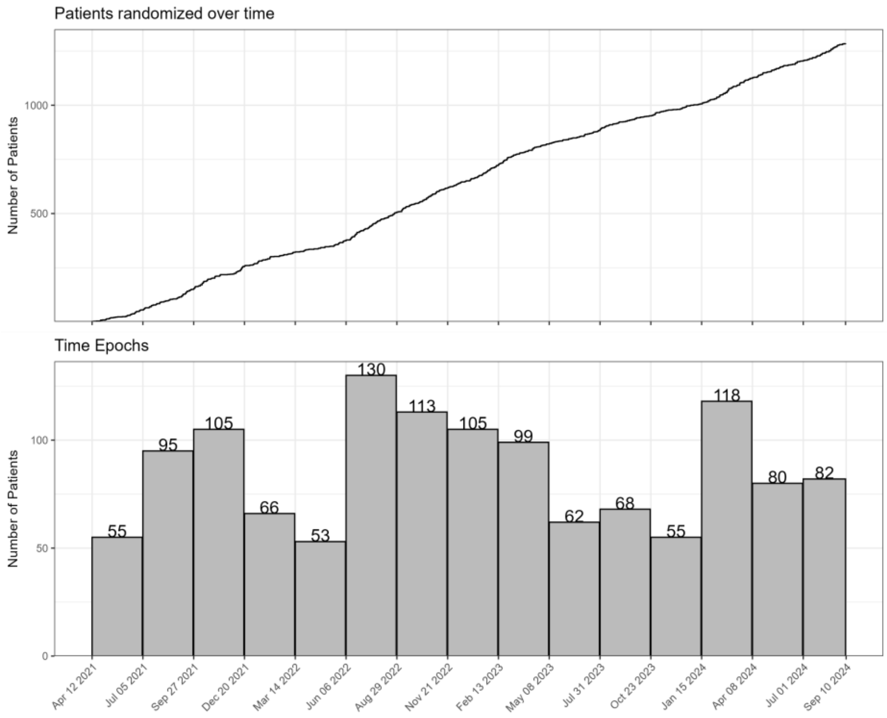

© 2026 Yende S et al. *JAMA Network Open*.

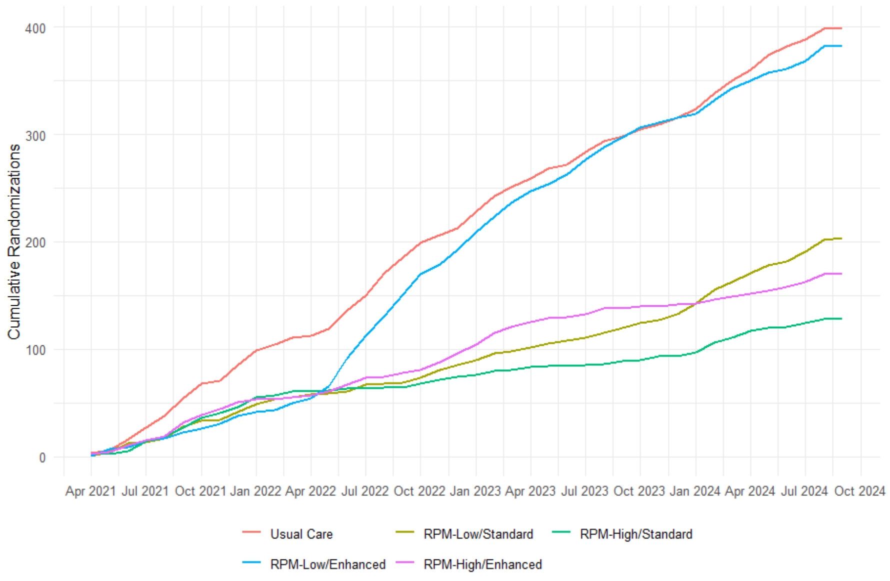

© 2026 Yende S et al. *JAMA Network Open*.

eFigure S3. Cumulative Probability Density Plots for Postdischarge Home Days by Intervention Arm

Cumulative probability distribution of post-discharge home days primary outcome by intervention arm over 90 days once patients were home. Cumulative proportion shown on the y-axis for each intervention group, with days since patients were home on the x-axis. The x-axis ordinal scale includes a score of -1 (death by day 90, the worst possible outcome), and scores ranging from 0 to 90 (the number of days alive and at home). This plot shows information very similar to a histogram, except instead of showing the proportion of each arm with a given value of the outcome, it shows the proportion of patients each arm with a given value or lower.

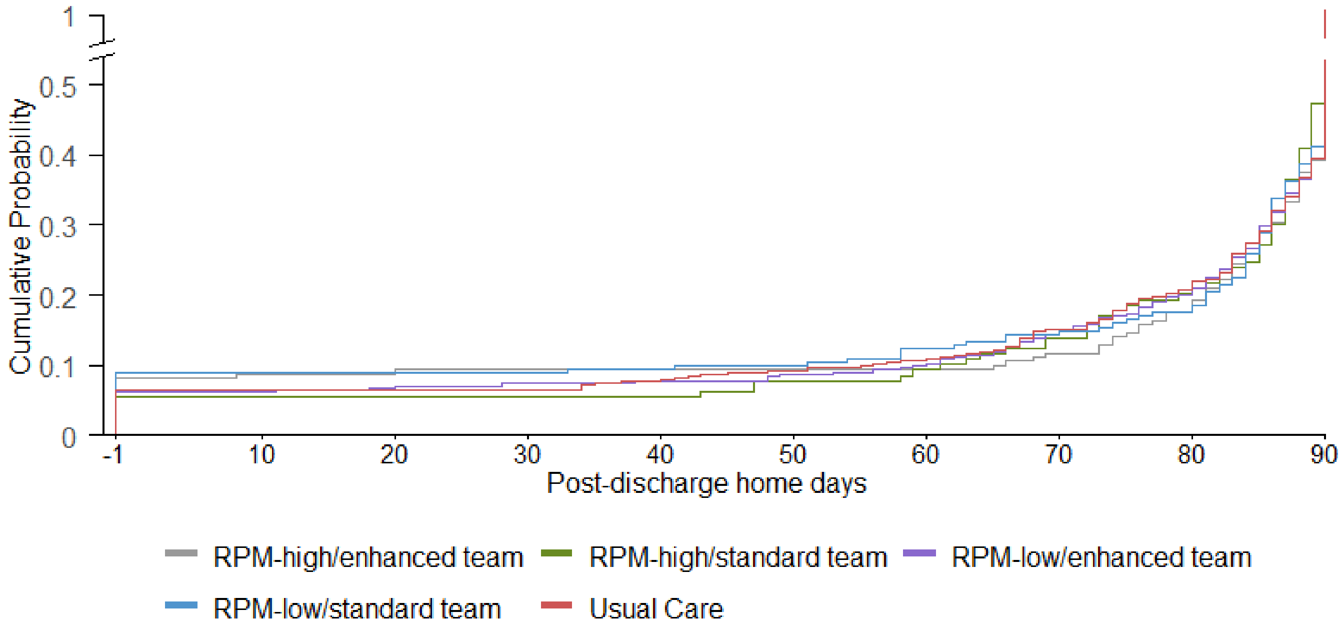

© 2026 Yende S et al. *JAMA Network Open*.

eFigure 4. Histograms of Postdischarge Home Days Outcomes by Intervention Arm

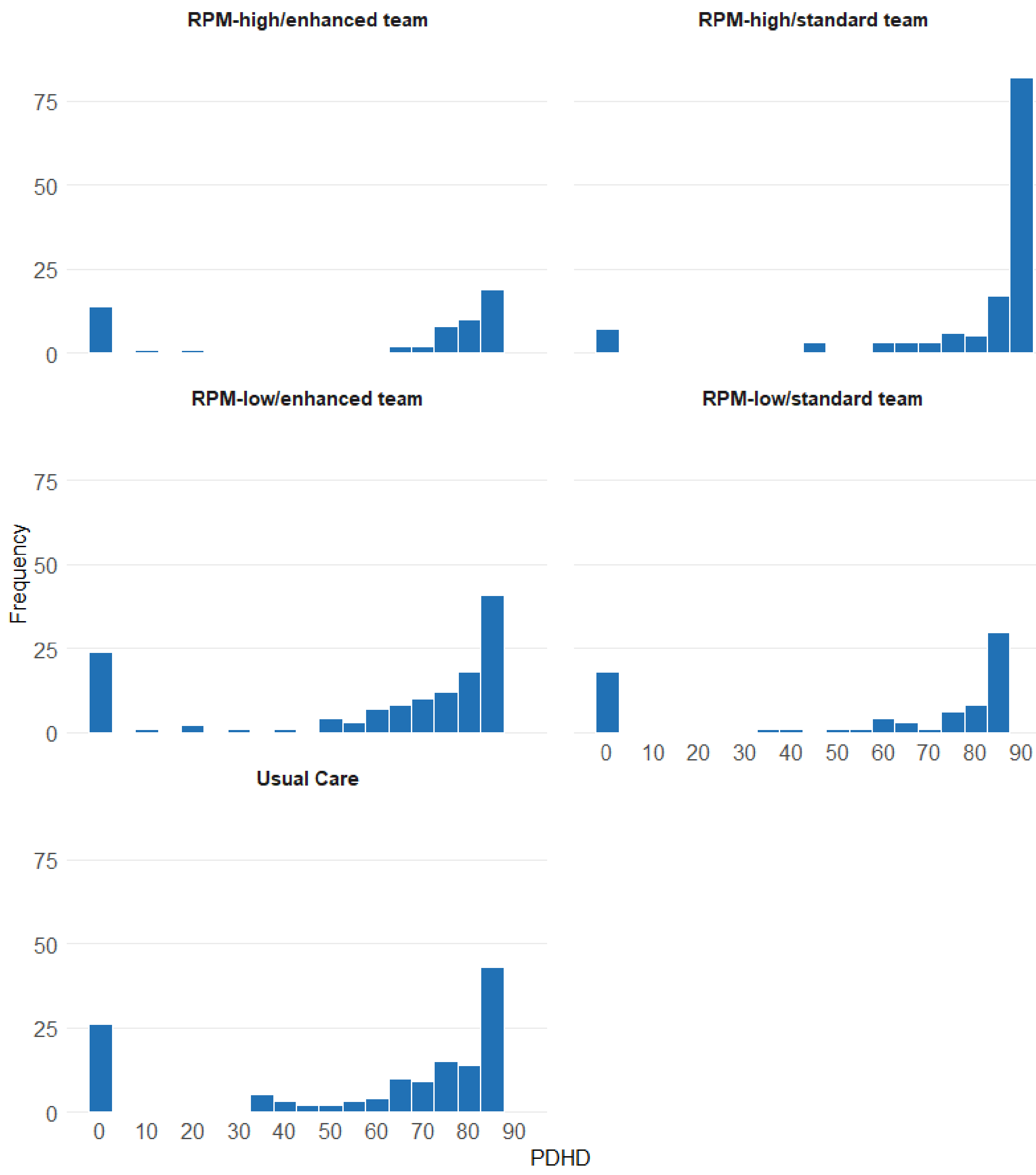

© 2026 Yende S et al. *JAMA Network Open*.

eFigure 5. Relationship Between Cumulative Odds Ratios and Absolute Differences in PDHDs

From the primary analysis model, we estimated cumulative odds ratios (CORs) and the corresponding absolute differences in the probability of patients staying at home for 90 days between each RPM arm and usual care. An absolute difference <0 indicates that the RPM arm had a lower probability of 90 PDHDs compared to usual care (suggesting that usual care is better), while a difference >0 indicates the RPM arm had a higher probability of 90 PDHDs than usual care (suggesting that RPM arm is better). The dashed vertical line at 0 indicates no difference. Across arms, absolute differences ranged from –3.6% (RPM-High/Standard) to 0.2% (RPM-Low/Enhanced) and the 95% credible interval crossed zero, indicating that the interventions had no effect on PDHD. Estimates assume patients were not severely ill and were not discharged to a skilled nursing facility, but results are not meaningfully different for the other strata.

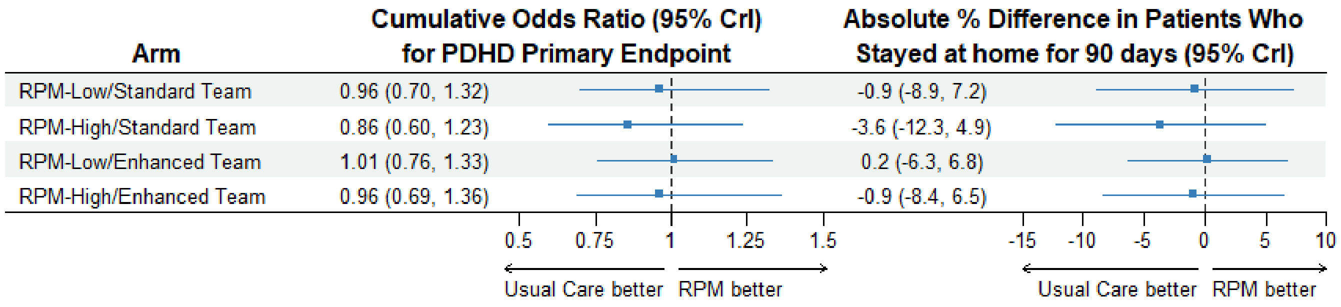

eFigure 6. Sensitivity Analysis of the Dichotomized Categories of PDHD

Each dot represents the odds ratio estimate for the dichotomized endpoint using the cutpoint indicated on the y-axis (where the dichotomization is ≤ cutpoint versus > cutpoint). The horizontal lines represent the 95% credible intervals of the odds ratio estimate for the dichotomized endpoint. These estimates are compared to the common odds ratio from the primary analysis where the solid vertical line is the posterior mean and the shaded region is the 95% credible interval. The dashed vertical lines show, for reference, an odds ratio of 1.0 (black).

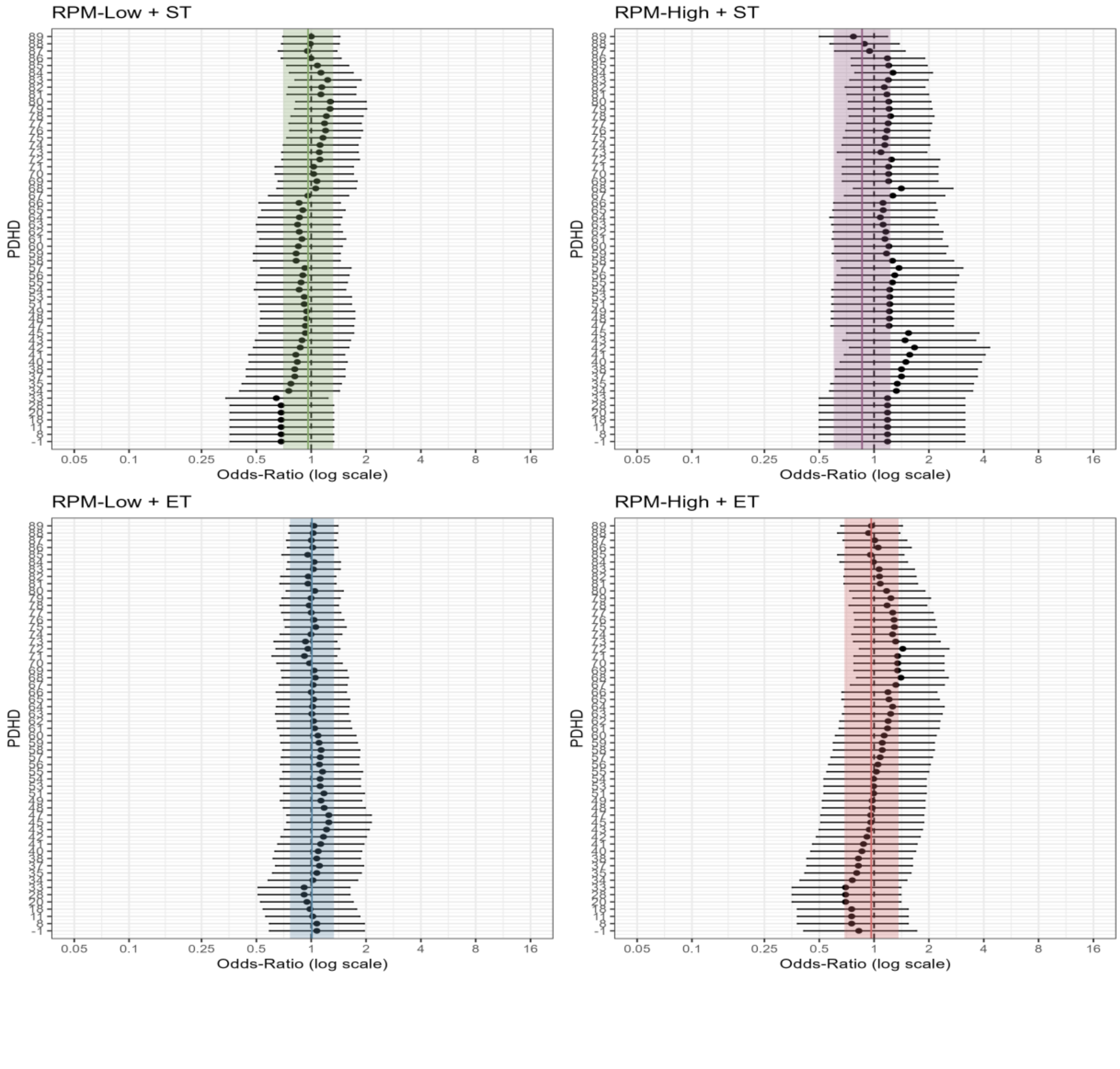

© 2026 Yende S et al. *JAMA Network Open*.

eFigure 7. Marginal Effects of RPM Intensity and Care Team Compared With Usual Care (UC) on Postdischarge Home Days

Arm	Cumulative Odds Ratio Estimate	Cumulative Odds Ratio 95% CrI	Probability of Superiority
RPM Intensity			
Low	0.99	0.77 to 1.27	47.0%
High	0.91	0.68 to 1.23	27.8%
Care Team			
Standard	0.92	0.69 to 1.23	28.3%
Enhanced	0.99	0.77 to 1.28	47.9%

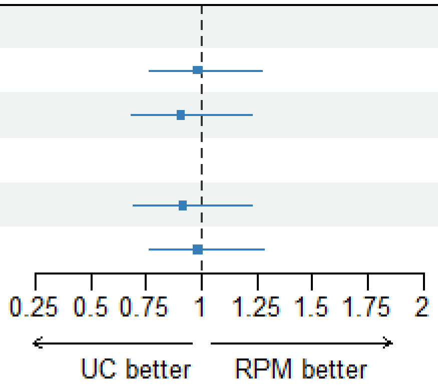

© 2026 Yende S et al. *JAMA Network Open*.

eFigure 8. Adjusted Hazard Ratios For Readmission Accounting for Competing Risk of Death by Intervention Arm

Adjusted hazard ratios for readmission accounting for death as a competing risk using a frequentist Fine-Gray subdistribution hazards model. Model adjusts for the same covariates as the primary analysis model: discharge location (home vs skilled nursing facility), severe illness, and randomization quarter. Larger subdistribution hazard ratios >1 indicate higher instantaneous risk of first readmission prior to the occurrence of death in the intervention arm compared to usual care.

	Subdistribution Hazard Ratio for Readmission	
Arm	Estimate	95% CI
RPM-low/standard team	0.99	0.75 to 1.31
RPM-high/standard team	1.24	0.91 to 1.68
RPM-low/enhanced team	0.99	0.78 to 1.25
RPM-high/enhanced team	0.98	0.72 to 1.33

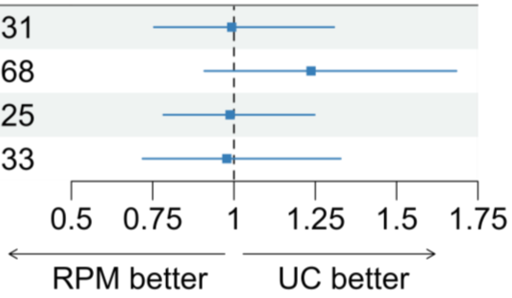

© 2026 Yende S et al. *JAMA Network Open*.

eFigure 9. Cumulative Incidence of Readmission Accounting for Competing Risk of Death by Intervention Arm

Unadjusted cumulative incidence of readmission by treatment arm estimated using Fine-Gray subdistribution hazards model accounting for death as a competing risk.

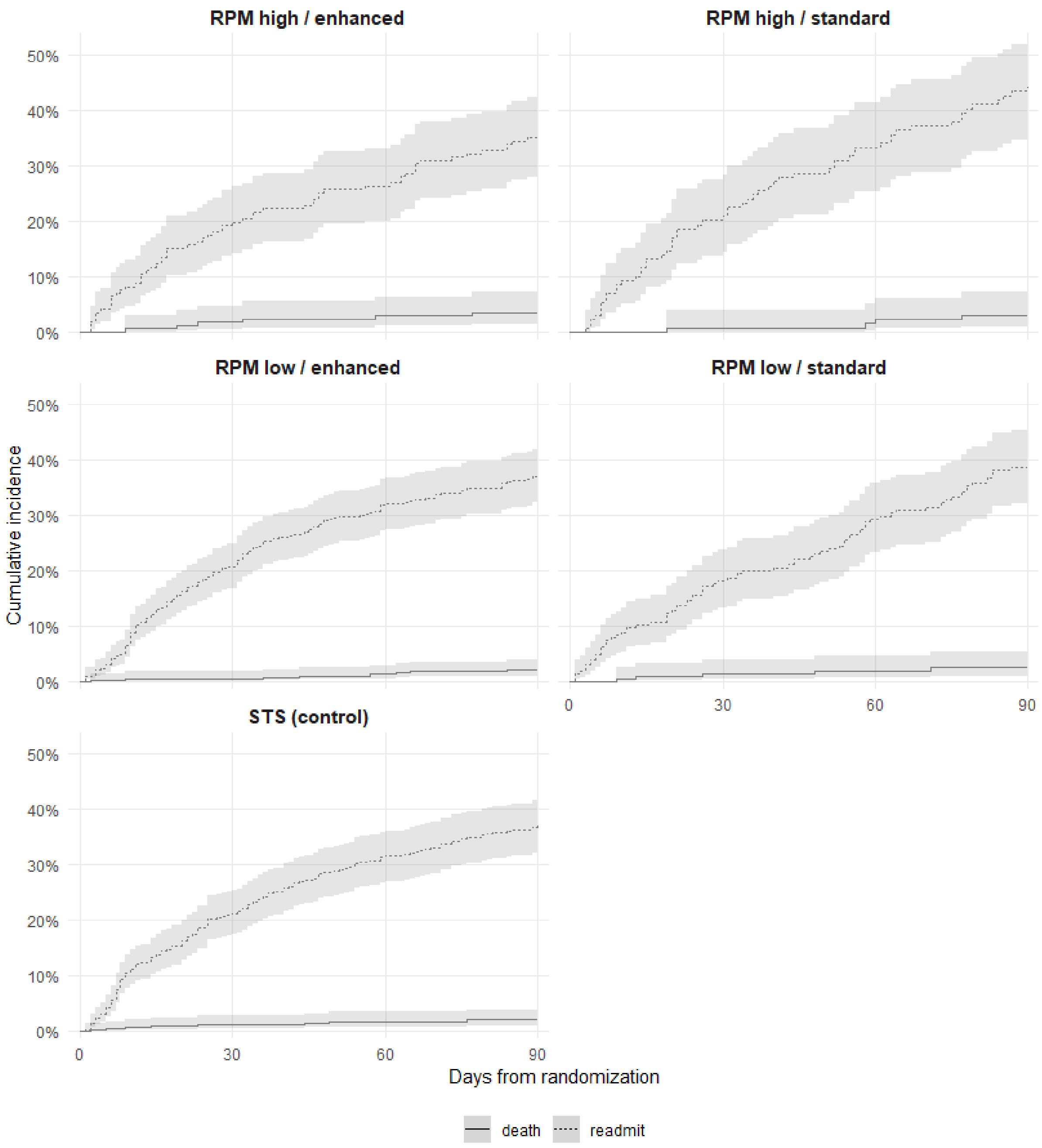

© 2026 Yende S et al. *JAMA Network Open*.

eTable 1. Criteria to Identify Sepsis, Respiratory Tract Infection, and COVID-19

These criteria were used to identify patients during medical record review by study coordinators. Additional review by a critical care physician was performed, as needed.

Condition	Criteria used by study coordinator
Sepsis	All three criteria were met:<br>• Clear source of infection (e.g., urinary tract infection, catheter-related blood stream infection, abscess) OR at least one culture (e.g., blood, urine, lower respiratory tract) ordered within the first three days<br>• Received at least two days of antibiotics, which were started within 24 hours of hospital presentation<br>• At least one criterion for organ dysfunction or notes mentioned “sepsis” or “septic shock”:<br>o Elevated serum creatinine and blood urea nitrogen levels (kidney function)<br>o Elevated serum lactate levels<br>o Received vasopressors (e.g., dobutamine, norepinephrine, epinephrine)
Lower respiratory tract infection	All three criteria were met:<br>• Received at least two days of antibiotics, which were started within 24 hours of hospital presentation<br>• At least one culture from blood or lower respiratory tract ordered within first three days of hospital presentation<br>Pneumonia or chronic obstructive pulmonary disease (COPD) exacerbation considered as a potential diagnosis
COVID-19	All three criteria were met:<br>• COVID-19 was the primary reason for hospital admission<br>• Presence of respiratory symptoms (e.g., shortness of breath, cough dyspnea)<br>• Prescribed/ordered one of the following during hospitalization:<br>o Dexamethasone (6 mg daily)<br>o One of the following: Remdesivir, Sotrovimab, Bebtelovimab, Paxlovid (nirmatrelvir tablets and ritonavir tablets, co-packaged for oral use), Tocilizumab, Baricitinib<br>o Received vasopressors, high-flow oxygen, invasive or non-invasive mechanical ventilation

© 2026 Yende S et al. *JAMA Network Open*.

eTable 2. Variables and Their Characteristics Used in the Predictive Model to Estimate the Risk of Readmission

Variable	High or moderate risk patients	Low risk patients
Charlson comorbidity index (mean)	7.8	4.9
Number of unique prescriptions in the past 6 months (mean)	12.1	8.5
Number of medical providers seen in the past 6 months (mean)	6	4.5
Age in years (mean)	70	54
Proportion of women (%)	57	65
Proportion with asthma (%)	25	18
Proportion with chronic obstructive lung disease (%)	54	36
Proportion with heart failure (%)	53	25
Proportion with diabetes (%)	59	35
Proportion with serious mental illness (%)	48	30

A risk prediction model was developed using UPMC data, incorporating over 40 variables collected at admission across more than 1.5 million records. The model was trained and validated on separate datasets to assess risk of 7 and 30-day readmission, achieving an area under the curve (AUC) of 0.75 in the test set. The overall 30-day readmission in moderate/high-risk patients was 16.5%.

© 2026 Yende S et al. *JAMA Network Open*.

eTable 3. Exclusion Criteria

Exclusion criteria
Admitted from a setting other than home or independent living
Enrollment in hospice prior to admission
Discharged to a skilled nursing facility for more than 28 days after index admission
Inability to participate in remote monitoring due to lack of an appropriate device or internet access
Known pregnancy
Severe persistent cognitive impairment[a]
No documented primary care physician (PCP) or if their PCP disapproves enrollment

© 2026 Yende S et al. *JAMA Network Open*.

eTable 4. Questionnaires Used for Remote Monitoring

	Question	Frequency
	**Questions Asked Across All RPM Arms**	
1	***Based on the thermometer, which number best describes how you are currently feeling?***<br>- 0, 1, 2: Skip all symptom questions<br>- 3, 4, 5, 6: Ask all applicable symptom questions<br>- 7-10 (high alert): Ask all applicable symptom questions	2x/week
2	***Are you having pain?***<br>- No<br>- Yes<br>○ ***Is your pain worse than usual?***<br>→ No<br>→ Yes (medium alert)<br>▪ *Pain Health Tip*	2x/week
3	***Are you having problems breathing?***<br>- No<br>- Yes<br>○ ***Is your breathing worse than usual?***<br>→ No<br>→ Yes (medium alert)<br>▪ *Shortness of Breath Health Tip*	2x/week
4	***Do you have nausea or vomiting?***<br>- No<br>- Yes<br>○ ***Are you unable to eat or drink because of nausea and/or vomiting?***<br>→ No<br>→ Yes (medium alert)	2x/week
5	***Do you have problems moving your bowels?***<br>- No<br>- Yes<br>○ ***Has it been more than 3 days since you had a bowel movement or is your stool hard to pass?***<br>→ No<br>→ Yes (medium alert)	2x/week
	**Healthwise video available**	

© 2026 Yende S et al. *JAMA Network Open*.

6	***Do you feel lightheaded or faint?***<br>- No<br>- Yes (medium alert)	2x/week
7	***Have you fallen in the past week?***<br>- No<br>- Yes (medium alert)<br><br>**Healthwise video available**	1x/week
8	***Do you have fever or chills?***<br>- No<br>- Yes (medium alert)<br><br>**Healthwise video available**	2x/week
9	***Do you feel like you must go to your doctor's office or emergency room in the next few days?***<br>- No<br>- Yes (medium alert)	2x/week
10	***Do you have questions about your medicines or medical conditions?***<br>- No<br>- Yes (medium alert)	2x/week
11	***Have you missed any doses of your antibiotic in the past three days?***<br>- No<br>- Yes (medium alert)	2x/week
12	***Have you started a new medicine in the last month?***<br>- No<br>- Yes (medium alert)	Monthly
13	**PHQ 2/9**	1x in week 1, then 1x every 8 weeks
14	**FAMCARE-P13**	1x in week 7, then 1x every 8 weeks

© 2026 Yende S et al. *JAMA Network Open*.

15	**Advanced Care Planning Video**	1x at 3 weeks until completed, followed by 1x weekly
16	**Choosing a Health Agent Video**	1x at 2 weeks until completed, followed by 1x weekly
**Additional Questions for Specific RPM Arms**		
17	RPM High Patients Only:<br><br>***Have you had a worsening cough or chest pain in the past three days?***<br>- No<br>- Yes (medium alert)	2x/week
18	RPM High Patients Only:<br><br>***Have you had swelling in your legs and ankles in the past 3 days?***<br>- No<br>- Yes (medium alert)	2x/week
19	RPM Enhanced Patients Only:<br><br>***Do you have someone who can help you with transportation, bill paying, and shopping?***<br>- Yes<br>- No (medium alert)	Monthly
20	RPM Enhanced Patients Only:<br><br>***Do you have problems caring for yourself at home?***<br>- No<br>- Yes (medium alert)	Monthly

© 2026 Yende S et al. *JAMA Network Open*.

eTable 5. Secondary End Points

Study Variables	Data Source	When Measured	How Measured
Functional status	Patient self-report	Baseline, 90 days	PROMIS[a] Physical Function – for Mobility Aid Users[80,81]
Health-related Quality of Life	Patient self-report	Baseline, 90 day	Quality of Life Enjoyment and Satisfaction Questionnaire-SF (QLESQ-SF)[82]
Transition to Hospice	Claims data	90 days	Whether member transitioned to hospice care
Emergent outpatient utilization	Claims data, EHR[b] data, HIE[c] data, patient self-report	90 days	Count of urgent outpatient visits (Emergency department and urgent care visits)
Inpatient Readmissions	Claims data, EHR data, HIE data, patient self-report	7, 30, and 90 days	Subsequent inpatient admission after index admission
Mortality	Claims data, EHR data, Caregiver	90 days	Whether member dies during observation period

[a]PROMIS – Patient-Reported Outcomes Measurement Information System; [b]EHR – Electronic Health Record; [c]HIE- Health Information Exchange

eTable 6. Power Calculations at the Onset of the Trial for Different Scenarios

Arms 1-4 represent the four intervention arms that were tested in the trial.

Scenario		Mean N						Probability of Success/Futility					Probability Selected as Optimal RPM Arm			
		Usual care	1	2	3	4	Total	1	2	3	4	Overall	1	2	3	4
1	Null	451	249	257	262	263	1482	0.006/ 0.252	0.006/ 0.257	0.006/ 0.249	0.006/ 0.267	0.023/ 0.047	0.247	0.24	0.261	0.252
2	One Weak	450	176	204	205	457	1492	0.006/ 0.270	0.009/ 0.186	0.011/ 0.175	0.299/ 0.055	0.308/ 0.018	0.042	0.053	0.057	0.848
3	One Expected	450	128	156	157	607	1498	0.001/ 0.279	0.014/ 0.144	0.006/ 0.131	0.851/ 0.011	0.851/ 0.006	0.007	0.003	0.001	0.989
4	One Strong	450	104	128	124	695	1500	0.004/ 0.321	0.009/ 0.120	0.013/ 0.135	0.986/ 0.006	0.987/ 0.004	0.001	0	0	0.999
5	Two Weak	451	156	357	158	375	1496	0.009/ 0.202	0.249/ 0.047	0.007/ 0.197	0.273/ 0.043	0.417/ 0.010	0.019	0.468	0.01	0.503
6	Two Expected	450	109	410	111	420	1500	0.004/ 0.180	0.712/ 0.004	0.003/ 0.192	0.725/ 0.003	0.918/ 0.002	0.002	0.476	0	0.522
7	Two Strong	450	91	427	92	440	1500	0.002/ 0.180	0.904/ 0.004	0.004/ 0.193	0.922/ 0.003	0.995/ 0.002	0	0.486	0	0.514
8	All Weak	450	253	258	266	272	1499	0.190/ 0.034	0.190/ 0.041	0.213/ 0.039	0.213/ 0.050	0.500/ 0.004	0.243	0.248	0.267	0.242
9	All Expected	450	245	267	266	272	1500	0.562/ 0.016	0.595/ 0.006	0.601/ 0.020	0.603/ 0.009	0.952/ 0.001	0.217	0.274	0.257	0.252
10	All Strong	450	243	262	272	272	1500	0.823/ 0.003	0.817/ 0.004	0.852/ 0.002	0.843/ 0.002	0.999/ 0.000	0.231	0.236	0.274	0.259

© 2026 Yende S et al. *JAMA Network Open*.

11	All Weak, Combo Additive	450	175	206	210	458	1500	0.113/ 0.051	0.205/ 0.037	0.216/ 0.036	0.808/ 0.013	0.865/ 0.001	0.052	0.052	0.048	0.848
12	All Expected, Combo Additive	450	125	163	162	599	1500	0.301/ 0.017	0.554/ 0.008	0.551/ 0.011	0.997/ 0.002	1.000/ 0.001	0.004	0.004	0.006	0.986
13	All Strong, Combo Additive	450	106	132	134	678	1500	0.500/ 0.007	0.778. 0.004	0.801/ 0.002	1.000/ 0.000	1.000/ 0.000	0	0	0	1

Note: Ground truth cumulative odds ratios (CORs) associated with each scenario label as follows: Null: COR=1; weak: COR=1.25; Expected: COR=1.5; strong: COR=1.75; all weak, combo additive: COR=1.25 for Standard Low, Standard High, Enhanced Low, and COR=1.56 for Enhanced High arm; all expected, combo additive: COR=1. 5 for Standard Low, Standard High, Enhanced Low, and COR=2.25 for Enhanced High arm; COR=1.75 for Standard Low, Standard High, Enhanced Low, and COR=3.06 for Enhanced High arm.

© 2026 Yende S et al. *JAMA Network Open*.

eTable 7. Revised Power Calculation Prior to Resizing in November 2023

These power calculation were based on a lower attrition rate. Based on these analyses, the trial was resized to 1,282 patients because this sample size maintained power above 80% for most effectiveness scenarios.

	Power for the primary hypothesis							
Scenario	N=1200	N=1250	N=1300	N=1350	N=1400	N=1450	N=1500	N Needed for 80% power (if feasible)
1, Null	~0.025	~0.025	~0.025	~0.025	~0.025	~0.025	0.023	NA
2, One Weak	0.256	0.265	0.274	0.282	0.291	0.299	0.308	NA
3, One Expected	0.765	0.782	0.798	0.812	0.826	0.839	0.851	1308
4, One Strong	0.963	0.969	0.974	0.978	0.981	0.984	0.987	<1000
5, Two Weak	0.347	0.359	0.371	0.382	0.394	0.406	0.417	NA
6, Two Expected	0.850	0.864	0.877	0.889	0.899	0.909	0.918	1049
7, Two Strong	0.982	0.985	0.988	0.990	0.992	0.994	0.995	<1000
8, All weak	0.418	0.432	0.446	0.460	0.474	0.487	0.500	NA
9, All Expected	0.900	0.911	0.921	0.930	0.938	0.946	0.952	<1000
10, All Strong	0.994	0.996	0.997	0.998	0.998	0.999	0.999	<1000
11, All weak combo additive	0.782	0.798	0.814	0.828	0.841	0.854	0.865	1255
12, All expected	>0.997	>0.998	>0.998	>0.999	>0.999	>0.999	1.000	<1000

© 2026 Yende S et al. *JAMA Network Open*.

combo additive 13, All strong, combo additive	>0.997	>0.998	>0.998	>0.999	>0.999	>0.999	1.000	<1000

Note: Ground truth cumulative odds ratios (CORs) associated with each scenario label as follows: Null: COR=1; weak: COR=1.25; Expected: COR=1.5; strong: COR=1.75; all weak, combo additive: COR=1.25 for Standard Low, Standard High, Enhanced Low, and COR=1.56 for Enhanced High arm; all expected, combo additive: COR=1. 5 for Standard Low, Standard High, Enhanced Low, and COR=2.25 for Enhanced High arm; COR=1.75 for Standard Low, Standard High, Enhanced Low, and COR=3.06 for Enhanced High arm

© 2026 Yende S et al. *JAMA Network Open*.

eTable 8. Baseline Characteristics by RPM Intensity and Team Composition

				RPM[a] Intensity		Care Team	
Patient characteristics	All Patients (N=1286)	Usual care (N=399)	Any care model (N=887)	Low (N=587)	High (N=300)	Standard (N=333)	Enhanced (N=554)
**Demographics**							
Age (years)	63 (54, 71)	64.0 (54, 71)	63.0 (53, 71)	62.0 (52, 71)	64 (55, 71)	63 (53, 71)	62.0 (53, 71)
Sex							
Female	665 (52)	194 (49)	317 (54)	154 (51)	168 (50)	303 (55)	665 (52)
Male	595 (46)	196 (49)	261 (44)	138 (46)	156 (47)	243 (44)	595 (46)
Other[b]	26 (2)	9 (2)	9 (2)	8 (3)	9 (3)	8 (1)	26 (2)
Race							
Black	199 (15)	66 (17)	93 (16)	40 (13)	54 (16)	79 (14)	199 (15)
White	1029 (80)	316 (79)	472 (80)	241 (80)	264 (79)	449 (81)	1029 (80)
Other[c]	58 (5)	17 (4)	22 (4)	19 (6)	15 (5)	26 (5)	58 (5)
Ethnicity							
Hispanic	11 (1)	4 (1)	4 (1)	3 (1)	1 (0)	6 (1)	11 (1)
Non-Hispanic	1211 (94)	377 (94)	559 (95)	275 (92)	309 (93)	525 (95)	1211 (94)
Unknown	64 (5)	18 (5)	24 (4)	22 (7)	23 (7)	23 (4)	64 (5)
**Health, education, and socioeconomic status before admission**							
Charlson Comorbidity Index[d]	6 (3, 9.0)	6.0 (4, 9)	6 (3, 8)	6 (3, 9)	6.0 (3, 8)	6.0 (3, 8)	6.0 (3, 9)
Prior admissions							
0	941 (80)	291 (79)	650 (80)	431 (80)	219 (80)	246 (79)	404 (80)
1	96 (8)	32 (9)	64 (8)	45 (8)	19 (7)	29 (9)	35 (7)
2	69 (6)	24 (7)	45 (6)	28 (5)	17 (6)	16 (5)	29 (6)
≥3	77 (7)	22 (6)	55 (7)	36 (7)	19 (7)	19 (6)	36 (7)
Area deprivation index[e]	7 (5, 9)	8 (5, 9)	7 (5, 9)	7 (5, 9)	7 (4, 9)	8 (5, 9)	7 (5, 9)

© 2026 Yende S et al. *JAMA Network Open*.

Insurance payer							
UPMC[f] Health Plan	1183 (92)	369 (92)	814 (92)	540 (92)	274 (91)	310 (93)	504 (91)
Medicare fee-for-service	103 (8)	30 (8)	73 (8)	47 (8)	26 (9)	23 (7)	50 (9)
Marital status							
Married	349 (27)	115 (29)	234 (26)	144 (25)	90 (30)	83 (25)	151 (27)
Never married	214 (17)	69 (17)	145 (16)	91 (16)	54 (18)	51 (15)	94 (17)
Separated / divorced	161 (13)	51 (13)	110 (12)	72 (12)	38 (13)	42 (13)	68 (12)
Widowed	106 (8)	22 (6)	84 (9)	63 (11)	21 (7)	34 (10)	50 (9)
Unknown	456 (35)	142 (36)	314 (35)	217 (37)	97 (32)	123 (37)	191 (34)
Comfort with technology							
Comfortable	581 (45)	183 (46)	398 (45)	252 (43)	146 (49)	141 (42)	257 (46)
Uncomfortable	242 (19)	75 (19)	167 (19)	108 (18)	59 (20)	68 (20)	99 (18)
Unknown	463 (36)	141 (35)	322 (36)	227 (39)	95 (32)	124 (37)	198 (36)
Health literacy							
Limited	183 (14)	45 (11)	138 (16)	91 (16)	47 (16)	56 (17)	82 (15)
Marginal	268 (21)	85 (21)	183 (21)	128 (22)	55 (18)	59 (18)	124 (22)
Adequate	378 (29)	129 (32)	249 (28)	146 (25)	103 (34)	96 (29)	153 (28)
Unknown	457 (36)	140 (35)	317 (36)	222 (38)	95 (32)	122 (37)	195 (35)
**Index hospitalization**							
Bed size	336 (145, 1120)	336 (163, 1120)	336 (145, 1120)	336 (145, 1120)	336 (145, 1120)	336 (145, 1120)	336 (145, 1120)
Teaching status	1274 (99)	398 (100)	876 (99)	581 (99)	295 (98)	329 (99)	547 (99)
Admission diagnosis							
Lower respiratory tract infection	367 (29)	113 (28)	254 (29)	158 (27)	96 (32)	111 (33)	143 (26)
Sepsis	606 (47)	188 (47)	418 (47)	288 (49)	130 (43)	161 (48)	257 (46)
COVID-19	313 (24)	98 (25)	215 (24)	141 (24)	74 (25)	61 (18)	154 (28)

© 2026 Yende S et al. *JAMA Network Open*.

Mechanical ventilation	62 (5)	23 (6)	39 (5)	27 (5)	12 (4)	20 (6)	19 (4)
Vasopressor use	144 (12)	47 (13)	97 (12)	65 (12)	32 (12)	42 (14)	55 (11)
Dialysis	100 (8)	37 (10)	63 (8)	46 (9)	17 (6)	25 (8)	38 (8)
Intensive care unit stay	386 (33)	113 (31)	273 (34)	186 (34)	87 (32)	110 (35)	163 (32)
Length of stay in the hospital	7 (4, 11)	7 (4, 11)	7 (4, 11)	7 (4, 11)	7 (4, 10)	7 (5, 11)	7 (4, 11)
Discharged to skilled nursing facility	110 (9)	36 (9)	74 (8)	53 (9)	21 (7)	28 (8)	46 (8)

Cells show median with interquartile range (IQR) for continuous variables and frequency (%) for binary variables; [a]RPM – remote patient monitoring; [ab] Other reported sexes include agender, gender queer and transgender; [bc] other reported races include American Indian / Alaska Native, Asian, and multi race. [a]RPM – remote patient monitoring; [bd]Charlson Comorbidity Index –; [ce]Area deprivation index calculated using state rankings; [df]UPMC – University of Pittsburgh Medical Center.

© 2026 Yende S et al. *JAMA Network Open*.

eTable 9. Fidelity and Engagement of Patients in the Remote Monitoring Pathways or Questionnaires

		RPM Intensity		Response team		Combined RPM intensity and response team			
	Any care model	Low	High	Standard	Enhanced	RPM low/ standard	RPM low/ enhanced	RPM high /standard	RPM high / enhanced
Number of patients enrolled in the arm	887	587	300	333	554	204	383	129	171
Enrolled in remote monitoring, n (% of enrolled in the study arm)	529 (60)	338 (58)	191 (64)	201 (60)	328 (59)	119 (58)	219 (57)	82 (64)	109 (64)
Pathways or questionnaires received by patients and responded									
Number received	10,561	6,793	3,768	3,943	6,618	2,290	4,503	1,653	2,115
Median (IQR) received	22 (9)	23 (9)	20 (10)	22 (9)	23 (10)	22 (10)	23 (9)	20 (9)	20 (10)
Number and proportion responded, n (%)	5,922 (56)	3,638 (53.6)	2,284 (60.6)	2,378 (60.3)	3,544 (53.6)	1,312 (57.3)	2,326 (51.7)	1,066 (64.5)	1,218 (57.6)
Median (IQR) responded	14 (9)	14 (10)	14 (8)	14 (10)	14 (8)	14 (11)	14 (9.5)	15 (7)	14 (7)
Alerts generated by patients in response to pathways or questionnaires									
Number of yellow alerts generated	3,166	1,712	1,454	1,255	1,911	641	1,071	614	840
Median (IQR) number of yellow alerts per patient	4(7)	4(6)	5(9)	5(8)	4(6)	4(8)	4(6)	6(9)	5(8.5)
Number of red alerts generated	425	222	203	154	271	75	147	79	124
Median (IQR) number of red alerts per patient	1(2)	1.5(2)	1(2.5)	1(2)	2(3)	1.5(2)	1.5(2.5)	1(2)	2(3)

[a]Pathways or questionnaires received by enrolled patients and responded.

© 2026 Yende S et al. *JAMA Network Open*.

eTable 10. Fidelity and Engagement for the 328 Patients Assigned to Enhanced Care Team

Intervention	Description
Virtual visit attempted within 7 days of hospital discharge	234 (71%)
Virtual visit completed[a]	139 (59%)
Medication review completed[a]	100 (72)
Goals of care discussion completed[a]	75 (54)

[a]Proportion calculated for patients in whom a virtual visit was attempted.

© 2026 Yende S et al. *JAMA Network Open*.

eTable 11. Effects of Study Interventions Compared With Usual Care on Secondary Outcomes

Outcome / Model	RPM-low/standard care		RPM high/standard care		RPM low/enhanced care		RPM high/enhanced care	
	Effect[a] (95% CrI)	Prob. >usual care[b]	Effect[a] (95% CrI)	Prob. >usual care[b]	Effect[a] (95% CrI)	Prob. >usual care[b]	Effect[a] (95% CrI)	Prob. >usual care[b]
7-day Readmission (composite with mortality)[c]	0.47 (0.22,0.97)	97.9	0.61 (0.25,1.39)	87.7	0.62 (0.32,1.17)	93.1	0.81 (0.36,1.74)	70.0
7-day Readmission (among survivors)[d]	0.53 (0.24,1.11)	95.4	0.73 (0.3,1.68)	76.8	0.67 (0.34,1.31)	87.7	0.95 (0.42,2.06)	54.8
30-day Readmission (composite with mortality)[c]	0.82 (0.52,1.28)	80.9	1.02 (0.61,1.68)	46.8	0.79 (0.54,1.16)	88.1	1.07 (0.66,1.7)	39.5
30-day Readmission (among survivors)[d]	0.79 (0.49,1.28)	82.9	1.00 (0.57,1.72)	49.5	0.91 (0.6,1.38)	67.0	1.11 (0.66,1.84)	35.2
90-day Readmission (composite with mortality)[c]	1.08 (0.77,1.52)	32.0	1.13 (0.76,1.65)	27.2	0.88 (0.66,1.19)	79.2	0.95 (0.66,1.37)	60.8
90-day Readmission (among survivors)[d]	0.98 (0.67,1.42)	54.2	1.11 (0.72,1.69)	32.3	0.88 (0.64,1.22)	77.6	0.89 (0.59,1.33)	70.9

© 2026 Yende S et al. *JAMA Network Open*.

PROMIS Physical Mobility score (composite with mortality)[e]	0.96 (0.67,1.39)	40.9	1.33 (0.86,1.94)	90.1	0.99 (0.7,1.46)	48	1.07 (0.75,1.48)	63.9
PROMIS Physical Mobility score (among survivors)[f]	-0.03 (-2.2,2.48)	48.9	1.73 (-1.09,3.82)	88.3	-0.18 (-2.24,2.05)	44.8	0.85 (-1.14,2.69)	80.2
Health-related Quality of Life (composite with mortality)[g]	0.94 (0.66,1.29)	34.9	1.13 (0.81,1.61)	75.8	0.96 (0.65,1.3)	41.0	0.95 (0.68,1.36)	39.0
Health-related Quality of Life (among survivors)[h]	0.00 (-0.06,0.05)	44.9	0.03 (-0.03,0.09)	80.6	0.00 (-0.07,0.05)	45.6	-0.01 (-0.07,0.05)	36.4
Number of emergency department visits (composite with mortality)[i]	1.08 (0.78,1.52)	32.0	0.92 (0.6,1.37)	66.3	0.92 (0.69,1.23)	70.6	1.03 (0.71,1.48)	43.9
Number of emergency department visits (among survivors)[j]	0.97 (0.67,1.4)	57.0	0.86 (0.55,1.33)	74.1	0.91 (0.66,1.26)	71.3	0.97 (0.65,1.44)	55.9
Hospice admission[k]	0.75 (0.44,1.24)	12.8	1.00 (0.56,1.77)	49.5	0.96 (0.61,1.51)	42.8	0.96 (0.53,1.66)	44.5
90-day mortality[l]	1.00 (0.74, 1.37)	36.7	0.99 (0.67, 1.53)	19.7	1.05 (0.79, 1.40)	52.2	1.09 (0.79, 1.40)	38.0

[a] Effects are reported as cumulative odds ratios (analyses marked with c, e, g, f, or j), odds ratios (analyses marked with d, k, l), or differences in means (analyses f or h).

© 2026 Yende S et al. *JAMA Network Open*.

[b] Probability of superiority over standard telephone support

[c] Assigned ordinal values as follows: 0=survive without readmission; 1=at least 1 readmission; 2=death. Cumulative odds ratios >1 suggest higher odds of more readmission or death in RPM arm as compared to STS

[d] Assigned values as follows: 0=no readmission; 1=at least 1 readmission. Odds ratios >1 suggest higher odds of readmission in RPM arm as compared to STS.

[e] Composite of death and PROMIS physical mobility score, measured at 90 +/- 7 days after randomization. Lowest value indicates death prior to measurement, and larger scores indicate better mobility status. Cumulative odds ratios >1 suggest higher odds of better mobiliity scores and survival in RPM compared to STS arm. N=411 patients were known to be alive at 90 days but were missing 90-day PROMIS scores. Scores for these patients were multiply imputed prior to estimating effects of RPM interventions. Imputation models were a function of the following covariates: age, CCI, ADI, baseline PROMIS score, whether the patient was living alone, UPMC health plan membership, comfort with technology, discharge to SNF, severity of illness, sex, race and treatment arm.

[f] PROMIS physical mobility score among survivors. Larger scores indicate higher mobility. Missing outcomes among patients known to be alive at 90 days were multiply imputed (see note e above). Mean differences >0 suggest higher mobility scores in RPM compared to STS.

[g] Composite of death and health related quality of life as measured by the Q-LES-SF at 90 +/- 7 days after randomization. Lowest value indicates death prior to measurement, and larger scores indicate better quality of life. Cumulative odds ratios >1 indicate higher odds of better quality of life and survival among RPM patients compared ot STS. The raw total score ranges from 14 to 70, for a total range of 56 points. For interpretation, the raw score is transformed into a percentage of the maximum possible score using the following formula: (raw total score - 14)/56. Missing HRQoL data were multiply imputed prior to modeling and treatment effect estimation. Imputation models were a function of the following covariates: age, CCI, ADI, baseline HRQoL, whether the patient was living alone, health plan membership, comfort with technology, discharge to SNF, severity of illness, sex, race and treatment arm.

[h] Q-LES-SF health-related quality of life score among survivors at 90 days. Larger scores indicate higher quality of life. Missing outcomes among patients known to be alive at 90 days were multiply imputed (see note g above). Mean differences >0 suggest higher quality of life scores among RPM patients compared to STS.

[l] Composite of number of emergency department visits and death by 90 days. Death was given a value higher than the largest number of observed emergency visits. Cumulative odds ratios >1 suggest higher odds of more more emergency department visits and death in RPM patinets compared to STS.

© 2026 Yende S et al. *JAMA Network Open*.

[j] Number of emergency visits among survivors to 90 days. Cumulative odds ratios >1 suggest higher odds of more more emergency department visits in RPM patinets compared to STS.

[k] Admission to hospice by day 90. Occurrence of death prior to admission to hospice was not treated as a competing risk.

[l] 90-day mortality estimated from the partial proportional odds model estimated in sensitivity analysis #1.

© 2026 Yende S et al. *JAMA Network Open*.

eTable 12. Results of Subgroup and Sensitivity Analyses

	Standard Low	Standard High	Enhanced Low	Enhanced High
**Sensitivity #1**				
PDHD in Survivors				
COR (95% CrI)	0.95 (0.71, 1.27)	0.86 (0.61, 1.21)	1.01 (0.77, 1.32)	0.95 (0.68, 1.32)
Pr(COR>1)	0.511	0.475	0.638	0.672
Mortality				
OR (95% CrI)	1.00 (0.74, 1.37)	0.99 (0.67, 1.53)	1.05 (0.79, 1.40)	1.09 (0.79, 1.40)
Pr(OR>1)	0.367	0.197	0.522	0.380
**Sensitivity #2**				
COR (95% CrI)	0.96 (0.70, 1.32)	0.86 (0.60, 1.23)	1.01 (0.77, 1.33)	0.96 (0.69, 1.36)
Pr(COR>1)	0.402	0.199	0.521	0.418
**Sensitivity #3**				
COR (95% CrI)	0.96 (0.70, 1.32)	0.86 (0.60, 1.23)	1.01 (0.77, 1.33)	0.97 (0.69, 1.36)
Pr(COR>1)	0.406	0.204	0.523	0.422
**Sensitivity #4**				
Payer: UPMC				
COR (95% CrI)	1.02 (0.74, 1.42)	0.86 (0.59, 1.24)	1.03 (0.77, 1.37)	0.91 (0.64, 1.30)
Pr(COR>1)	0.550	0.202	0.573	0.292
Payer: MC FFS				
COR (95% CrI)	0.64 (0.26, 1.55)	1.02 (0.32, 3.32)	0.87 (0.36, 2.13)	1.55 (0.54, 4.72)
Pr(COR>1)	0.164	0.513	0.379	0.793
**Sensitivity #5**				
Payer: UPMC				
COR (95% CrI)	1.02 (0.74, 1.43)	0.86 (0.60, 1.24)	1.02 (0.77, 1.36)	0.90 (0.63, 1.29)
Pr(COR>1)	0.558	0.212	0.556	0.282

© 2026 Yende S et al. *JAMA Network Open*.

Payer: MC FFS				
COR (95% CrI)	0.53 (0.20, 1.39)	0.83 (0.23, 2.95)	0.80 (0.31, 2.08)	1.38 (0.44, 4.53)
Pr(COR>1)	0.098	0.383	0.326	0.706
**Sensitivity #6**				
Payer: UPMC				
COR (95% CrI)	1.15 (0.76, 1.73)	0.92 (0.57, 1.51)	0.96 (0.67, 1.36)	0.78 (0.50, 1.25)
Pr(COR>1)	0.744	0.376	0.409	0.150
Payer: MC FFS				
COR (95% CrI)	0.62 (0.25, 1.54)	1.02 (0.32, 3.33)	0.85 (0.35, 2.08)	1.57 (0.54, 4.75)
Pr(COR>1)	0.150	0.514	0.361	0.797
**Sensitivity #7**				
Payer: UPMC				
COR (95% CrI)	1.02 (0.74, 1.43)	0.86 (0.60, 1.24)	1.02 (0.77, 1.36)	0.90 (0.63, 1.29)
Pr(COR>1)	0.558	0.212	0.556	0.282
Payer: MC FFS				
COR (95% CrI)	0.53 (0.20, 1.39)	0.83 (0.23, 2.95)	0.80 (0.31, 2.08)	1.38 (0.44, 4.53)
Pr(COR>1)	0.098	0.383	0.326	0.706
**Sensitivity #8**				
COR (95% CrI)	1.06 (0.75, 1.50)	0.86 (0.59, 1.26)	0.95 (0.71, 1.28)	0.86 (0.60, 1.24)
Pr(COR>1)	0.627	0.222	0.377	0.209

Note: Sensitivity analysis #1 explored the effects of RPM interventions on PDHD among survivors and on mortality, estimated using a single partial proportional odds model. Sensitivity analysis #2 explored the impact of including a term for a proportional shift in PDHD between payer types (UPMC Health Plan vs Medicare Fee-for-Service) into the primary analysis model. Sensitivity analysis #3 explored a non-proportional shift between payer types. Sensitivity analysis #4 added an interaction term for payer type to the primary analysis model to explore differential effects of RPM models on PDHD across different payer types. Sensitivity #5 estimated effects by payer type via two separate models. Sensitivity analyses #6 and #7 estimated effects by payer type among those who enrolled after August 10, 2022 (the date that enrollment of Medicare Fee-for-Service patients began) via interaction and stratified

© 2026 Yende S et al. *JAMA Network Open*.

models, respectively. Sensitivity #8 estimated effects of RPM arms among patients with UPMC Health Plan coverage throughout follow-up.

© 2026 Yende S et al. *JAMA Network Open*.

eTable 13. Probability of Differential Treatment Response Across Prespecified Subgroups

Subgroup Comparison	Standard Team	Enhanced Team
**Prespecified analyses**		
Age <65 vs ≥65	99.9	99.7
Male vs Female	61.9	79.5
White vs Black race	90.6	89.0
CCI<5 vs CCI≥5	85.6	90.9
Comfortable vs not comfortable with technology	78.1	68.1
Health literacy level		
Limited vs Marginal	61.3	66.8
Limited vs Adequate	61.4	80.9
Marginal vs Adequate	70.9	91.2
Area Deprivation Index		
Quartile 1 vs Quartile 2	70.4	79.7
Quartile 1 vs Quartile 3	65.2	64.0
Quartile 1 vs Quartile 4	61.7	77.3
Quartile 2 vs Quartile 3	73.5	62.8
Quartile 2 vs Quartile 4	58.3	91.7
Quartile 3 vs Quartile 4	57.3	62.3
SNF vs Home	97.1	99.9
Severe vs Not Severe	81.4	50.5
Diagnosis at index admission		
COVID vs Sepsis	89.7	72.7
COVID vs Lower respiratory tract infection	50.6	50.6
Sepsis vs Lower respiratory tract infection	71.2	71.2
**Post-hoc analyses**		
Enrollment in Remote Monitoring	90.7	56.3
Enrollment era (COVID era vs Post-COVID)	58.8	90.0

Note: Probability of differential treatment response calculated as the maximum of the posterior probability of the difference between subgroup cumulative log odds ratios being >0 and <0.

© 2026 Yende S et al. *JAMA Network Open*.

eTable 14. Mortality, Hospital Readmission, and Hospice Admission Outcomes Stratified by Age Group

Age group / Outcome	Usual Care	Standard Team	Enhanced Team	All patients
Age < 65				
90-day mortality	14 (6.5)	11 (5.8)	12 (3.9)	37 (5.2)
Emergency department admission	66 (30.4)	54 (28.6)	100 (32.5)	220 (30.8)
Inpatient readmission	80 (36.9)	56 (29.6)	87 (28.2)	223 (31.2)
Other readmission	45 (20.7)	23 (12.2)	47 (15.3)	115 (16.1)
Any readmission	97 (44.7)	69 (36.5)	110 (35.7)	276 (38.7)
Total readmission days	8 (4,18)	4 (2,14)	8 (3,18)	7 (3,17)
Hospice				
Using claims data	40 (19.3)	34 (18.9)	49 (17)	123 (18.2)
Using claims and Medicare carve-out	40 (19.3)	35 (19.4)	50 (17.4)	125 (18.5)
Age ≥ 65				
90-day mortality	12 (6.6)	14 (9.7)	26 (10.6)	52 (9.1)
Emergency department admission	48 (26.4)	37 (25.7)	61 (24.8)	146 (25.5)
Inpatient readmission	48 (26.4)	56 (38.9)	75 (30.5)	179 (31.3)
Other readmission	22 (12.1)	28 (19.4)	41 (16.7)	91 (15.9)
Any readmission	54 (29.7)	69 (47.9)	95 (38.6)	218 (38.1)
Total readmission days	8 (4,24)	6 (3,18)	7 (4,17)	7 (4,19)
Hospice				
Using claims data	5 (3.1)	4 (3.1)	7 (3.2)	16 (3.1)
Using claims and Medicare carve-out	10 (6.2)	7 (5.4)	20 (9.3)	37 (7.3)

Cells chow frequency (%) for all variables; Readmissions variables count death regardless of 90-day mortality status; Total length of readmissions was calculated as the total number of days spent in the hospital, conditional on having at least one readmission, up to day 90. Hospice use was calculated using claims data and additionally with a Medicare carve-out indicator, since hospice services for Medicare Advantage patients are billed through Medicare fee-for-service and were not available.

© 2026 Yende S et al. *JAMA Network Open*.

eTable 15. Mortality, Hospital Readmission, and Hospice Admission Outcomes Stratified by Discharge Location

Discharge location group / Outcome	Usual Care	Standard Team	Enhanced Team	All patients
Discharged home				
90-day mortality	26 (7.2)	23 (7.5)	30 (5.9)	79 (6.7)
Emergency department admission	103 (28.4)	83 (27.2)	151 (29.7)	337 (28.7)
Inpatient readmission	122 (33.6)	104 (34.1)	150 (29.5)	376 (32)
Other readmission	62 (17.1)	44 (14.4)	74 (14.6)	180 (15.3)
Any readmission	142 (39.1)	126 (41.3)	186 (36.6)	454 (38.6)
Total readmission days	8 (4,22)	5 (3,15)	8 (4,17)	7 (3,17)
Hospice				
Using claims data	43 (12.9)	35 (12.4)	51 (11.1)	129 (12)
Using claims and Medicare carve-out	48 (14.5)	39 (13.8)	62(13.6)	149 (13.9)
Discharged to skilled nursing facility				
90-day mortality	0 (0)	2 (7.1)	8 (17.4)	10 (9.1)
Emergency department admission	11 (30.6)	8 (28.6)	10 (21.7)	29 (26.4)
Inpatient readmission	6 (16.7)	8 (28.6)	12 (26.1)	26 (23.6)
Other readmission	5 (13.9)	7 (25)	14 (30.4)	26 (23.6)
Any readmission	9 (25.0)	12 (42.9)	19 (41.3)	40 (36.4)
Total readmission days	3 (2,4)	12 (5,26)	12 (5,28)	6 (3,25)
Hospice				
Using claims data	2 (5.3)	3 (10.7)	5 (10.9)	10 (9.1)
Using claims and Medicare carve-out	2 (5.3)	3 (10.7)	8 (17)	13 (11.5)

Cells chow frequency (%) for all variables; Readmissions variables count death regardless of 90-day mortality status; Total length of readmissions was calculated as the total number of days spent in the hospital, conditional on having at least one readmission, up to day 90. Hospice use was calculated using claims data and additionally with a Medicare carve-out indicator, since hospice services for Medicare Advantage patients are billed through Medicare fee-for-service and were not available.

© 2026 Yende S et al. *JAMA Network Open*.

eTable 16. Characteristics of the Cohort Participating in qualitative Analyses

Patient characteristics	All Patients (N=84)
**Demographics**	
Age (years)	62.5 (53.8, 70.0)
Female sex	46 (55)
Race	
White	63 (80)
Black	16 (20)
Other	5 (6)
Ethnicity	
Hispanic	1 (1)
Non-Hispanic	80 (95)
**Health, education, and socioeconomic status before admission**	
Charlson Comorbidity Index[b]	6.0 (3.0, 8.0)
Prior admissions	
0	63 (76)
1	11 (13)
2	5 (6)
≥3	4 (5)
Area deprivation index[c]	8.0 (5.0, 9.0)
Insurance payer	
UPMC[d] Health Plan	83 (99)
Medicare fee-for-service	1 (1)
Marital status	
Married	34 (40)
Never married	20 (24)
Separated / divorced	15 (18)
Widowed	11 (13)
Unknown	4 (5)
Comfort with technology	
Comfortable	53 (63)
Uncomfortable	26 (31)
Unknown	5 (6)
Health literacy	
Limited	25 (30)
Marginal	15 (18)
Adequate	39 (46)
Unknown	5 (6)

© 2026 Yende S et al. *JAMA Network Open*.

**Index hospitalization**	
Bed size	336.0 (143.5, 1120.0)
Teaching status	84 (100)
Admission diagnosis	
Lower respiratory tract infection	28 (33)
Sepsis	29 (35)
COVID-19	27 (32)
Mechanical ventilation	4 (5)
Vasopressor use	9 (11)
Dialysis	4 (5)
Intensive care unit stay	31 (37)
Length of stay in the hospital	6.5 (4.0, 11.2)
Discharged to skilled nursing facility	2 (2)

© 2026 Yende S et al. *JAMA Network Open*.

eTable 17. Illustrative Quotes from Qualitative Analyses

Theme		Quote(s)
Utilizing Remote Monitoring Technology	Usability	*"It was user friendly. That was pretty easy doing the questions each week, when I could remember to do them. Somebody always sent me a message when they wanted the questions answered." [Enhanced High]*<br><br>*"I mean it was only, what, twice a week. So, it wasn't like it was overwhelming or anything. So no, I think the timing was as good as far as you know, when you had to go and answer the questions and that." [Enhanced High]*<br><br>*"I wasn't overwhelmingly bothersome at all, and with limited questions which made it very easy." [Standard High]*<br><br>*"It was nice, but I guess the frequency, to know that it was only twice a week, so if I would have problem, it was there but it wasn't too much. If I needed it, I could've gotten a hold of someone, just very convenient." [Enhanced Low]*<br><br>*"Because it whenever I would get it I would fill it out. And if I would be unsure about something. I knew that chat button was right there. And all I had to do was message, and somebody would get back to me within... I'd say 20 minutes. If not sooner. And I like that. You know, like if you call your doctor's office, you might get a phone call that day. But most of the time you have to wait. I love the fact that I was answered right away. And that I didn't feel alone in this... I mean it's scary enough, having health issues, especially something you don't know why it's happening and everything... There was a friendly, loving voice at the end of the line that was reassuring me. That meant a lot to me." [Standard Low]*
	Factors limiting engagement	*"At times I wouldn't do it, you know I just wasn't feeling up to filling it out, you know it seemed like an intrusion or something. I just wasn't up for it you know a couple times." [Standard Low]*<br><br>*"And I just keep forgetting Tuesdays and Thursdays, that's what it used to be on. So I don't know, that's the way they did it and I just keep forgetting about it actually, it's not on your part it's mine I just keep forgetting." [Standard Low]*<br><br>*"I got questions all the time. And you know some of them it's just yes and no you know, sometimes the answer is a maybe. You know, or a none of the above. You know it's not just black and white yes or no, you know it's a… I don't know it gets annoying to …because I got a different answer than yes or a no." [Standard Low]*

© 2026 Yende S et al. *JAMA Network Open*.

		"No, they did not call me over the phone no, I only got the text that I answered the questions." [Enhanced Low]
	Lack of interpersonal connection	*I've mixed feelings on that. It was OK. I had no problem with it, but. I missed the one-on-one contact. They could hear my questions but, when you're in a doctor's office and seeing your doctor and he asks you how are you feeling today and you say oh, OK, he can tell by your oh, OK and you're shaking your head a little, but you're not exactly 100%. And so he would follow up on that. Most doctors would. But in an automated thing, they can't see that, you know. [Enhanced High]*<br><br>*"I want to talk to somebody. I don't want to talk to a machine or anything. I don't want to have to put it on a computer. Just I want to talk to somebody. You want to know what I want to feel, call me. I'll talk to you." [Enhanced Low]*
Impact of Remote Monitoring Interventions	Reassurance	*"I would say there's a bit of the fear when you go home that you might have a relapse and that even though you know. I have a good support system in my family. And then I think it was, it gave a feeling of being cared for and some confidence that [hospital system] is monitoring my condition and wants to keep me out of the hospital, doesn't want me to come back. So, it's more like an emotional for me it was more an emotional feeling of support and care. So, I thought it was all good." [Standard High]*<br><br>*"No, just like I said it was just nice to have somebody there that I know I can count on if something come up. That was the best part." [Enhanced High]*

© 2026 Yende S et al. *JAMA Network Open*.

	Alert Response	*"Well the only thing they would do, when I talked to them the only thing they would do is just advise me to go to the ER or call someone. Because there wasn't nothing that they could do you know what I'm saying. So and they would always advise me to go to the hospital if I needed emergency care or if there's uncomfortable is to go to the ER." [Enhanced High]*<br><br>*"I liked that if I did put I was in distress or in any type of pain, [RN] would call me right away and we would talk about like, what my symptoms were at that time. And then at one point, like I said, my breathing since having COVID has been a little bit worse than how my asthma normally is. So he would suggest like hey, call your doctor get, you know, a [Rx] paper or, you know, whatever the case may be. And so I did like that." [Enhanced Low]*<br><br>*"Oh well he would call in case the scale come up like one to, I don't know what it was six seven eight, I don't know. But I always put three that I was in slight distress and then that's why he called to see what was going on and whatever. And I told him what I felt was going on and we chitchatted and that was fine, he brought me down some I wasn't as nervous. I guess I was." [Enhanced High]*<br><br>*"I believe the couple of times she told me to call my doctor or one time, she told me to call the doctor and the other time, she directed me to go to the emergency room." [Enhanced High]*

© 2026 Yende S et al. *JAMA Network Open*.
