# Supplementary material for: Remote Monitoring Approaches to Reduce Readmissions After Infection and Sepsis: A Randomized Clinical Trial
Source: JAMA Netw Open. 2026 Jun 11;9(6):e2616641. doi: 10.1001/jamanetworkopen.2026.16641 (PMC13261490; doi:10.1001/jamanetworkopen.2026.16641)
Supplement: Supplement 3. — Data Sharing Statement [file jamanetwopen-e2616641-s003.pdf]

Data Sharing Statement

Yende. Remote Monitoring Approaches to Reduce Readmissions After Infection and Sepsis. *JAMA Netw Open*. Published June 11, 2026. doi:10.1001/jamanetworkopen.2026.16641

Data
Additional Information: This trial was registered at clinicaltrials.gov (NCT04829188) on 4/1/2021. Trial ethical approval: This trial was approved by the University Institutional Review Board (STUDY20080130).
Data available: Yes
Data types: Deidentified participant data, Data dictionary
How to access data: Data underlying the findings of this study are available upon reasonable request. Requests must align with Institutional Review Board approval and may include deidentified data for replication purposes, as well as the study protocol and statistical analysis plan. Requests should be submitted to the UPMC Center for High-Value Health Care and will require a data use agreement. Data will be available beginning 6 months after publication and for up to 36 months thereafter. Requests may be directed to the corresponding author, Dr. Sachin Yende (yendes@upmc.edumailto:sachin.yende@va.gov).
When available: With publication

Supporting Documents
Document types: Other (please specify)
Additional Information: Statistical analysis plan and trial protocol are included with the submission.
How to access documents: Statistical analysis plan and trial protocol are included with the submission.
When available: With publication

Additional Information
Who can access the data: Anyone
Types of analyses: NA
Mechanisms of data availability: NA
Any additional restrictions: NA
